# Dearomatization of Aromatic Carbonyl Compounds by Photocycloaddition Reactions to 1,1-Dimethylallene

**DOI:** 10.1021/acs.orglett.6c00429

**Published:** 2026-03-02

**Authors:** Luis Oxenfart, Julian Zuber, Nina Strassner, Thorsten Bach

**Affiliations:** Technische Universität München, TUM School of Natural Sciences, Department Chemie and Catalysis Research Center (CRC), 85747 Garching, Germany

## Abstract

The photocycloaddition of 1,1-dimethylallene to various aromatic carbonyl compounds was found to occur exclusively at the benzene core. While the reaction with methyl 2-methoxybenzoate resulted in a mixture of products resulting from *ortho*, *meta*, and *para* photocycloaddition, acetophenone and its 4-substituted derivatives delivered the respective *para* photocycloaddition products in 50–58% yields. For 2-methoxyacetophenone, an *ortho* photocycloaddition initiated a reaction cascade, which led to bicyclic products by the incorporation of a nucleophile in 29–74% yields.

## Graphical abstract

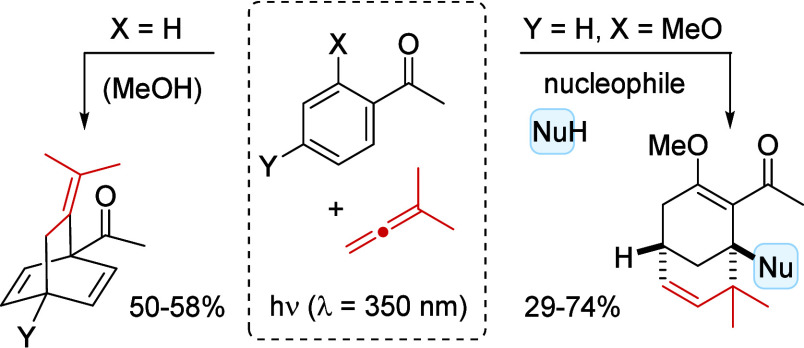

Photocycloaddition reactions belong to the most powerful transformations of photochemistry.^1^ With aromatic carbonyl compounds as substrates, four structurally distinct primary products are conceivable when they are irradiated in the presence of an olefin (Scheme 1). The [2+2] photocycloaddition at the carbonyl group, the Paternò–Büchi reaction,^2^ leads to an oxetane, while the arene core can undergo a photocycloaddition in a 1,2-fashion (*ortho*), a 1,3-fashion (*meta*), and a 1,4-fashion (*para*). Arguably, the latter reactions enable access to more complex structures than the Paternò–Büchi reaction. Furthermore, they offer a carbon–carbon bond-forming reaction at the benzene core and a concomitant dearomatization^3^ as additional features.^4^ Primary products from a *meta* and *para* photocycloaddition can typically be isolated, whereas *ortho* photocycloaddition products frequently encounter ensuing reactions resulting from a thermally allowed disrotatory ring opening of the cyclohexadiene ring. The question of which photocycloaddition pathway is favored depends largely on the nature of the chromophore, but the olefin component can also play a critical role. Since most benzaldehydes and phenyl ketones populate rapidly the lowest-lying triplet state that has nπ* character,^5^ their photochemistry is to a large extent dominated by the carbonyl group, particularly in apolar solvents.^2^ However, exceptions exist, and photocycloaddition pathways beyond the Paternò–Büchi reaction have potential to be utilized in total synthesis campaigns.^6^

**Scheme 1:**
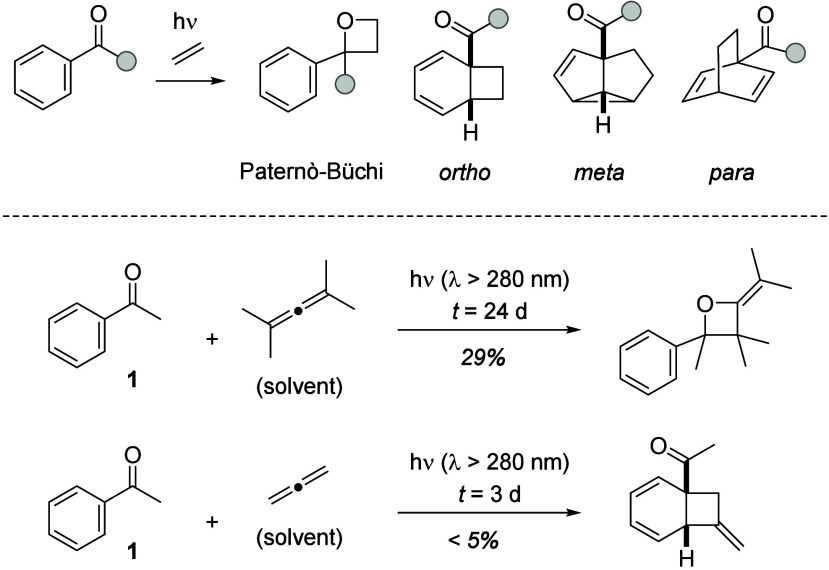
Photocycloaddition Pathways Accessible to Aromatic Carbonyl Compounds and Examples of Allene Photocycloadditions to Acetophenone (**1**)

In the context of our work on the deracemization of acyclic allenes,^7^ we came across the photocycloaddition of acetophenone (**1**) to allenes. Seminal studies by Gotthardt, Steinmetz, and Hammond had revealed that the compound undergoes the expected Paternò–Büchi reaction when irradiated with 1,1,3,3-tetramethylallene (2,4-dimethyl-2,3-pentadiene).^8^ The oxetane product with an exocyclic olefin was isolated in 29% yield (Scheme 1), and the product of a 2-fold acetophenone addition (not shown) was obtained in 38%. Remarkably, when parent allene was employed as the olefin component, Gotthardt and Hammond observed the addition of the compound to the benzene core of acetophenone as a side reaction.^9^ Although the product was isolated in low yield, the result indicated to us that it might be worth exploring the photocycloaddition chemistry of aromatic carbonyl compounds to allenes in more detail. In this work, we now disclose our first preliminary results obtained with 1,1-dimethylallene (3-methyl-1,2-butadiene) as the olefin component.

The choice for this specific allene as a substrate was triggered by the fact that it displays, unlike the previously mentioned allenes, two distinctly different olefinic bonds, allowing us to interrogate a regiochemical preference at the allene. In addition, unlike the parent allene, the compound^10^ is a liquid at room temperature, which is easy to handle and can be readily prepared from propargylic alcohol. In initial experiments performed with **1** at a wavelength λ of 300 and 350 nm in various solvents, it turned out that methanol provided the cleanest conversion. An excess of the allene was required to drive the reaction to completion. The reaction was faster at 300 nm but cleaner at 350 nm. Surprisingly, the only isolable product under these conditions was a symmetric compound with only two distinct olefinic protons in the ^1^H NMR spectrum. The tetrasubstituted olefin resulting from the allene was still present, indicating that the unsubstituted allene double bond had been involved in the photocycloaddition. In agreement with our assignment, single-crystal X-ray analysis confirmed the product to be *para* photocycloaddition product **2a** (Scheme 2; all ORTEP representations are shown with 50% probability displacement ellipsoids).

**Scheme 2:**
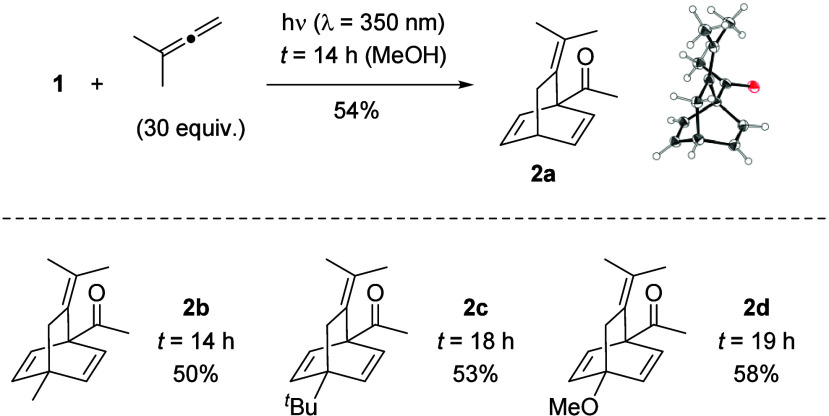
*para* Photocycloaddition Products **2** Obtained from the Reaction of 1,1-Dimethylallene with Acetophenone (**1**) and its 4-Substituted Derivatives

In the product, the terminal 1,1-dimethylsubstituted olefin resides in β-position to the carbonyl group with one methyl group pointing toward it. Without further optimization, other 4-substituted acetophenones were subjected to the reaction in the presence of 1,1-dimethylallene. Irradiation was continued until no aromatic ketone could be detected by TLC. In all reactions, *para* photocycloaddition products **2b**–**2d** were the only compounds that could be isolated and characterized. If benzaldehyde was subjected to the same reaction conditions, the reaction was sluggish, and only products from reactions at the carbonyl group were detectable. The observation of a *para* photocycloaddition contrasts with the reaction of 1,1-dimethylallene with a phenyl alkynyl ketone (λ > 280 nm) in a benzene solution, which resulted exclusively in oxetane formation.^11^ However, there is also precedent for *para* photocycloaddition reactions of allenes to a benzene ring. Mariano and co-workers, for example, reported on the *para* photocycloaddition of 1,1-dimethylallene to a phenyl-substituted iminium ion (λ > 260 nm) in an acetonitrile solution.^12^ An intramolecular *para* photocycloaddition of allenes to benzaldehydes was observed by the Bochet group upon irradiation at 350 nm in a CH_2_Cl_2_ solution.^13^

The reaction course of the photocycloaddition dramatically changed when 2-methoxyacetophenone (**3**) was employed as the carbonyl compound (Scheme 3). Under the conditions applied for the conversion of **1** to **2a**, the major product was bicyclic nonadiene **4a**. The compound was isolated as a single diastereoisomer, and it was immediately evident that the solvent had been added to a photocycloaddition intermediate.

**Scheme 3:**
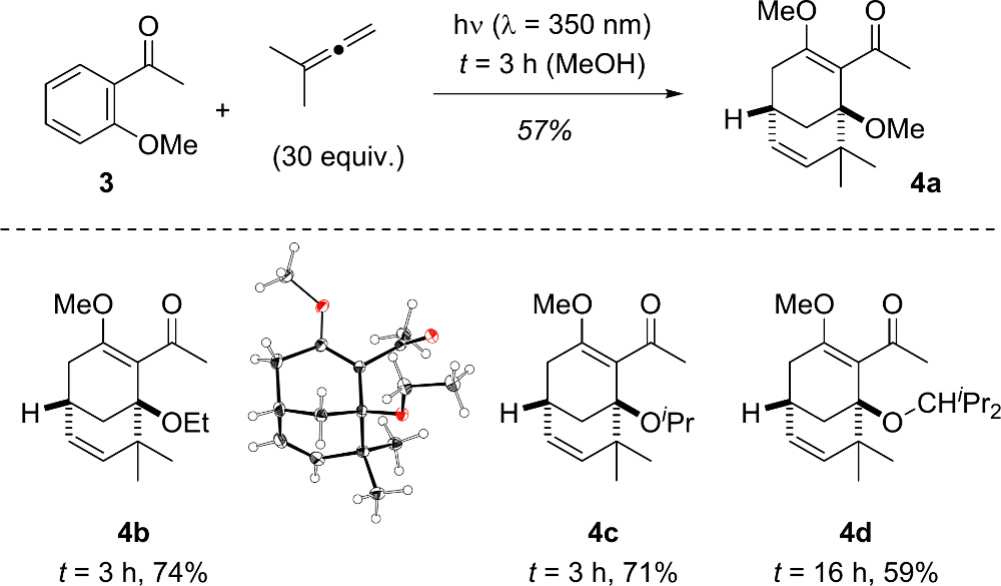
*ortho* Photocycloaddition of 1,1-Dimethylallene to 2-Methoxyacetophenone (**3**) Delivering Bicyclic Solvent Addition Products **4**

The reaction proceeded analogously in ethanol (to **4b**), isopropanol (to **4c**), or 2,4-dimethylpentanol (to **4d**) as the solvent. In all cases, the alcohol was incorporated into the product, and yields varied between 59% and 74%. The product of the reaction in ethanol turned out to be crystalline, and its structure was confirmed by single-crystal X-ray diffraction. To the best of our knowledge, the observed reaction pathway is unprecedented for photocycloaddition reactions of allenes to a benzene core. The increase in structural complexity is remarkable given the planar structure of the substrate.

Mechanistically, we propose that the photocycloaddition of ketones **1** and **3** proceeds via their rapidly populated triplet state^14^ and starts with the addition of 1,1-dimethylallene to the carbon atom adjacent to the carbonyl group (Scheme 4). After intersystem crossing (ISC), resulting diradical intermediate **5** can immediately cyclize to *para* photocycloaddition product **2a** for X = H. This pathway seems to be disfavored for X = MeO, in the case of which addition to the *ortho*-carbon atom is the predominant cyclization mode. In contrast to **2a**, the dimethyl substituents of the allene seem to be easier to accommodate within the newly formed ring than at the exocyclic double bond. Primary *ortho* photocycloaddition product **6** is unstable and undergoes a thermally allowed disrotatory ring opening to yield intermediate cyclooctatriene **7**. At this stage, we postulate the attack of the alcohol (ROH) to occur, resulting in the formation of addition products **4**. Support for the intermediacy of cyclobutane **6** stems from its isolation, when the reaction was performed in dichloromethane at −40 °C, and from compound **8**, which was detected as a byproduct in the reaction performed in methanol. The addition of methanol to the enol ether double bond stalls the reaction sequence, with the disrotatory ring opening pathway being inaccessible for **8**.

**Scheme 4:**
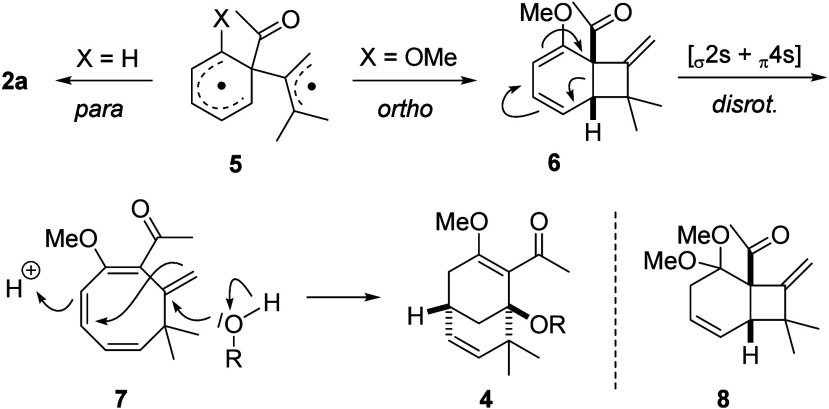
Mechanistic Proposal for the Formation of Products **2** and **4** and Structure of Byproduct **8**

Since we speculated that intermediate **7** could also be trapped by other nucleophiles, which would ideally not be used as the solvent, we changed the solvent to dichloromethane. An initial experiment was performed with 4,4-dimethylcyclohexanol, which confirmed that the alcohol was not required to be the solvent (Scheme 5). Ether **4e** was obtained in 59% yield after an irradiation time of 6 h. When (+)-menthol was used as the nucleophile, a moderate diastereoselectivity (d.r., diastereomeric ratio) was observed in the putative cyclization step from **7** to **4f**. The two diastereoisomers could be separated with the major diastereoisomer being isolated in 36% yield and the minor diastereoisomer in 22% yield. The structure assignment remains tentative due to the flexibility of the O–C linkage, which precludes strong nuclear Overhauser effect (NOE) contacts between the menthyl group and the bicyclononadiene entity (see the Supporting Information for details). Benzoic acid was used successfully to trap putative intermediate **7**, resulting in ester **9** as the product. Given the oxidative power of photoexcited acetophenone,^15^ it came as a pleasant surprise that potential reductants such as 1-adamantanethiol and neopentylamine could also be employed in the photocycloaddition–cyclization sequence. Thioester **10** was isolated in 29% yield, and secondary amine **11** in 58% yield. Even in the less polar solvent dichloromethane, there was no indication of any reaction pathways besides the *ortho* photocycloaddition, previously observed in alcoholic solvents. Attempts to involve 2-methylacetophenone in a related sequence were not successful. Upon irradiation in the presence of 1,1-dimethylallene (λ = 350 nm; MeOH; *t* = 14 h), a slow conversion was observed, and only the product of a *para* photocycloaddition was detected.

**Scheme 5:**
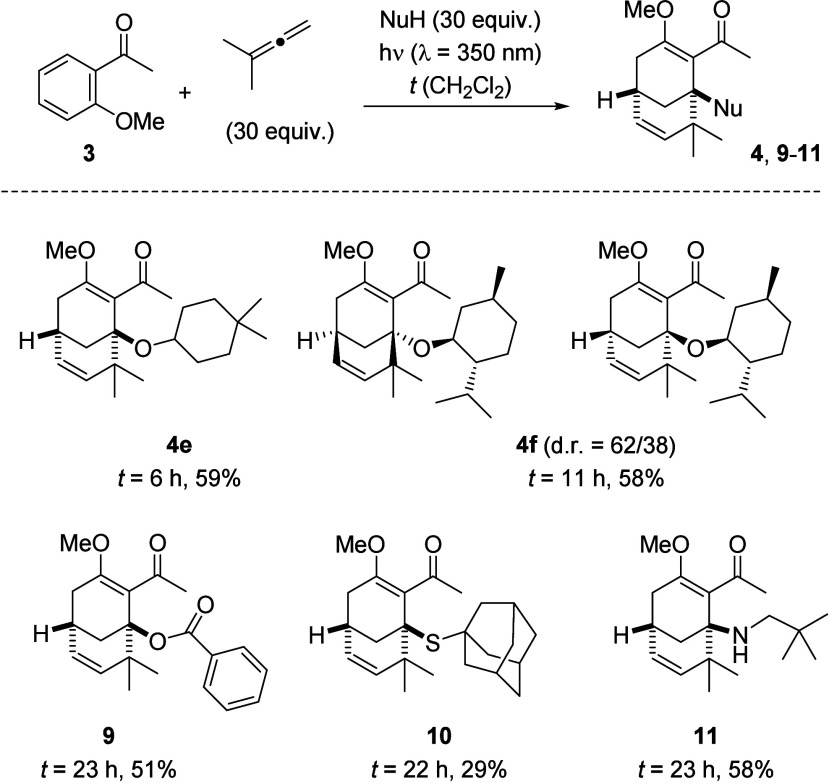
*ortho* Photocycloaddition of 1,1-Dimethylallene to 2-Methoxyacetophenone (**3**) in CH_2_Cl_2_ Delivering Bicyclic Nucleophile Addition Products **4** and **9–11**

Salicylic acid derivative methyl 2-methoxybenzoate **12** was employed as a final arene substrate in the photocycloaddition to 1,1-dimethylallene (Scheme 6). Previous work on the intramolecular photocycloaddition of salicylic acid derivatives had revealed that an appropriately O-tethered alkene at the phenoxy oxygen atom adds *ortho* to the benzene core.^16^ From the structure of consecutive products, it was deduced that the initial attack occurred at the two substituted arene C1 and C2 atoms. In this case, the regioselectivity of the approach is likely dictated by the tether, which enforces the formation of a five- or six-membered ring. We are not aware of studies of the intermolecular photocycloaddition to a salicylate derivative.

**Scheme 6:**
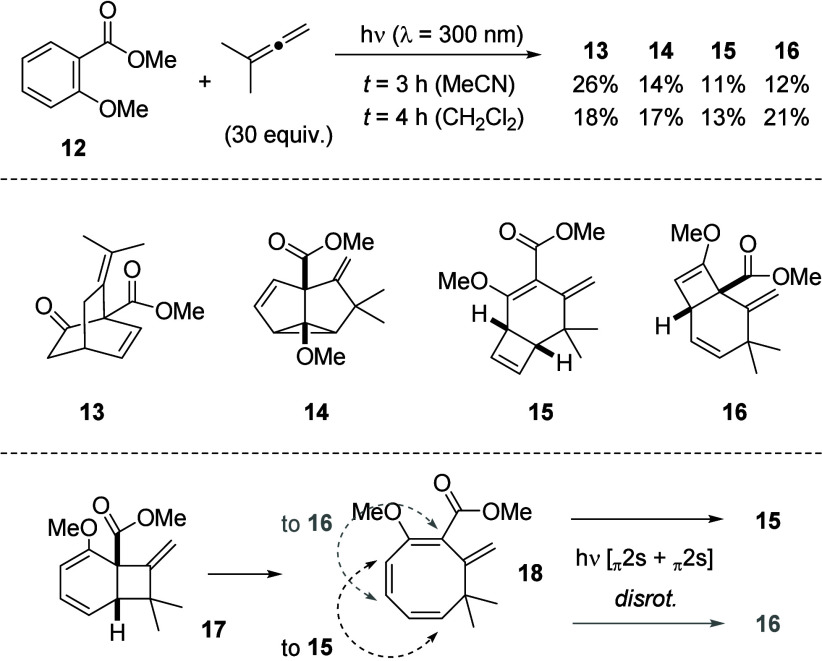
Diverse Photocycloaddition Products **13–16** Obtained from the Reaction of Methyl 2-Methoxybenzoate (**12**) and 1,1-Dimethylallene

Due to the blue-shifted absorption of ester **12** relative to ketone **1** and **3**, irradiation experiments with 1,1-dimethylallene were not successful at 350 nm. At 300 nm, there was no single distinct product to be identified in either of the solvents we studied (MeOH, MeCN, CH_2_Cl_2_, and hexafluoroisopropanol). However, the reactions in acetonitrile and dichloromethane delivered product mixtures from which individual compounds could be identified. Since all products are 1:1 photocycloaddition products and have roughly the same molecular mass, a yield can be given, even though they were not isolated in pure form. Remarkably, products emanating from all three photocycloaddition modes (*ortho*, *meta*, and *para*)^4^ were identified. The primary *para* photocycloaddition product was likely an enol ether, which appears to be hydrolyzed during workup, resulting in the isolation of ketone **13**. For product **14**, no consecutive reactions were observed, and its structure was tentatively assigned based on NMR data, suggesting a *meta* photocycloaddition^17^ (see the Supporting Information for details). The bicyclo[4.2.0]­octa-2,7-diene core observed in methyl esters **15** and **16** is characteristic for consecutive products of an *ortho* photocycloaddition. In analogy to intermediate **7** (cf. Scheme 4), we propose the formation of cyclooctatriene **18** from **17**, which continues to react photochemically in a disrotatory [4π] photocyclization to regioisomeric products **15** and **16**.

In summary, our study provides another example of the structural diversity that can be achieved in photocycloaddition reactions to the arene core. The question of whether the exclusive reaction at the benzene core observed for **1** and **3** is due to the increased ππ* character of their triplet state,^14a,14b^ the choice of the olefin, or both remains open. From a synthetic perspective, the clean *para* photocycloaddition reactions to afford products **2** and the *ortho* photocycloaddition of 2-methoxyacetophenone (**3**) stand out. In the latter context, it is remarkable how the consecutive chemistry of *ortho* photocycloaddition products can lead to structurally complex scaffolds with several exit vectors.

## Supplementary Material





## Data Availability

The data underlying this study are available in the published article and its .

## Notes

FAIR data, including the primary NMR FID files, for compounds **1–16** (ZIP)
Materials and methods, synthetic procedures and full characterization for all products (**2a–d**, **4a–f**, **9–11**, and **13–16**) and the products of the allene synthesis route, spectroscopic data, NMR spectra, including representative 2D spectra, and computational data (PDF)
