# Supplementary material for: Dearomatization of Aromatic Carbonyl Compounds by Photocycloaddition Reactions to 1,1-Dimethylallene
Source: Org Lett. 2026 Mar 2;28(10):3331–5. doi: 10.1021/acs.orglett.6c00429 (PMC12993915; doi:10.1021/acs.orglett.6c00429)
Supplement: Supplementary file 2 [file ol6c00429_si_002.pdf]

Supporting Information

Dearomatization of Aromatic Carbonyl Compounds by Photocycloaddition Reactions to 1,1-Dimethylallene

Luis Oxenfart,[a] Julian Zuber,[a] Nina Strassner,[a] and Thorsten Bach*[a]

[a]*Technische Universität München, School of Natural Sciences, Department of Chemistry and Catalysis Research Center, Lichtenbergstrasse 4, 85747 Garching, Germany.*

*** Corresponding Author**

Email: thorsten.bach@ch.tum.de

Table of Contents

1. General Remarks 3

2. Allene Synthesis 8

3. General Procedure 1 (GP1): Converting *para*-Substituted Acetophenone Derivatives with 1,1-Dimethylallene 9

4. General Procedure 2 (GP2): Synthesis of Bicyclic Nucleophile Addition Products in Alcohols as the Solvent 14

5. General Procedure 3 (GP3): Synthesis of Bicyclic Nucleophile Addition Products in Dichloromethane as Solvent 19

6. Synthesis of Bicyclic Nucleophile Addition Product with Benzoic Acid as Nucleophile 25

7. Synthesis of Bicyclic Nucleophile Addition Product with Neopentylamine as Nucleophile 26

8. Synthesis of Photocycloaddition Products from 2-Methoxybenzoic Acid Methyl Ester 27

9. Crystallographic Data 34

10. NMR Spectra of New Compounds 38

11. References 68

12. XYZ Coordinates 69

1. General Remarks

All air- and water-sensitive reactions were performed in flame-dried glassware under argon atmosphere and under anhydrous conditions using *Schlenk* techniques unless otherwise stated. Heating baths were filled with silicone oil and regulated by electronic thermometers. Cooling baths contained ice and water (0 °C).

Solvents and reagents: Dry acetonitrile (MeCN), isopropanol (^{i}PrOH), and methanol (MeOH) were obtained from Acros in the highest available purity (>99.5%, extra dry over molecular sieves). Dry ethanol (EtOH) was obtained from Acros in the highest available purity (99.5%). These solvents were used without further purification. Technical solvents used for aqueous workup and purification by column chromatography [diethyl ether (Et_2O), dichloromethane (CH_2Cl_2), ethyl acetate (EtOAc), methanol (MeOH), pentane (P), toluene ($PhCH_3$)] were distilled prior to use. Commercially available chemicals were obtained from BLDPharm, Sigma Aldrich and TCI Europe and used as received unless otherwise stated. 2,4-Dimethyl-3-pentanol (DM-3-P), used as solvent for photochemical optimization reactions and as nucleophilic solvent for the photochemical formation of bicyclic solvent addition products, was dried over activated molecular sieve (3 Å) for at least 14 hours.

Chromatography: Thin layer chromatography (TLC) and preparative thin layer chromatography (pTLC) were performed on pre-coated glass-backed Merck Kieselgel 60 F254 plates with visualization effected with ultra-violet irradiation (λ = 254) and/or staining using Seebach's "magic" stain (SM) prepared from phosphomolybdic acid (5.00 g), cerium(IV) sulfate (2.00 g), H_2O (100 mL), and concentrated H_2SO_4 (5.00 mL). The TLC plates were subjected to subsequent heat treatment (ca. 250 °C) in order to complete the staining process. Flash column chromatography was performed on silica 60 (Merck, 230–400 mesh) with the indicated eluent mixtures.

High Resolution Mass Spectroscopy (HRMS): High resolution mass spectrometry was performed on an LTQ FT Ultra (ESI) from Thermo Scientific. Each value obtained was within 4 ppm of the calculated mass.

Melting points (m.p.): Melting points were determined using a *Kofler* heating bar designed by L. Kofler (Reichert) without correction.

Rotational values: Specific optical rotations were determined using a Bellingham & Stanley polarimeter ADP400+ and is reported as follows: $[\alpha]_D^T$ (c in g per 100 mL solvent).

Infrared spectroscopy (IR): IR spectra were recorded on a Bruker Vertex 70 Fourier transform infrared (FT-IR) spectrometer using the attenuated total reflection (ATR) technique. The respective intensities are described by the following abbreviations: w (weak), m (medium), s (strong), and br (broad). C_{alkene} refers to an unspecific alkene vibration.

NMR spectroscopy: ^{1}H NMR spectra were recorded on Bruker AVHD-400, AVHD-500, AV NEO-400, AV NEO-500 or a Bruker AV-II-500 equipped with a cryo probe head. All spectrometers were operating at 298 K at 400 MHz and 500 MHz, respectively. Data is reported in the following manner: chemical shift [in parts per million (ppm) relative to residual $CHCl_3$ (δ_H = 7.26 ppm)], number of protons, multiplicity and coupling constant *J* (measured in Hz to the nearest 0.1 Hz). The multiplicity of a signal is indicated as: s-singlet, bs-broad singlet, d-doublet, t-triplet, q-quartet, hept-heptet, m-multiplet, or combinations of those. Apparent multiplets which occur because of coupling constant equality between magnetically non-equivalent protons are marked as virtual (*virt*.). ^{13}C NMR spectra were recorded on AVHD-400, AVHD-500, AV NEO-400 or AV NEO-500 spectrometers at 298 K operating at 101 MHz and 126 MHz respectively with proton decoupling. The chemical shift [in parts per million (ppm)] is reported relative to residual $CHCl_3$ (δ_C = 77.16 ppm). Spectra are reported based on appearance in combination with theoretical multiplicities derived from structural information. Assignments of ^{1}H and ^{13}C NMR signals were based on heteronuclear single quantum coherence (HSQC), heteronuclear multiple-bond correlation (HMBC), ^{1}H-^{1}H-correlation spectroscopy (COSY), and nuclear Overhauser effect spectroscopy (NOESY) experiments. Diastereomeric ratios were calculated based on the ratio of the integrals of the corresponding best-defined peaks in the respective ^{1}H NMR spectrum.

Density Functional Theory (DFT) calculations: DFT calculations were conducted in order to facilitate the assignment of the diastereomers **4f**. Conformational searches were performed using the Conformer–Rotamer Ensemble Sampling Tool (CREST) with the GFN2-xTB method.[1] Low-energy conformers were optimized in the Gaussian 16 software package[2] using the ωB97xD functional[3] and triple-zeta def2TZVP basis set.[4] To best account for the NMR solvent, implicit solvation with the CPCM solvation model for chloroform[5] was included in the DFT calculations. All geometries were optimized without any restrictions and verified as true minima by the absence of negative eigenvalues in the vibrational frequency analysis. All Gibbs free energies are reported with the enthalpic and entropic quasi-harmonic GoodVibes correction at 298 K.[6] Results were visualized using PyMOL.[7]

Crystal preparation: All crystals were prepared by adding hexanes (previously distilled) dropwise to the sample until all the material was dissolved. The open vial was placed in a freezer until the solvent was evaporated resulting in the formation of crystals at the bottom of the flask.

Irradiation experiments: Photochemical reactions under direct irradiation conditions (λ = 350 nm) were performed in flame-dried Duran tubes (diameter = 1.00 cm) in a positive geometry setup (cylindrical array of 16 UVA lamps, 8 W nominal power) with the sample placed in the center of the illumination chamber (distance to the UVA lamps: 10.5 cm).[8] In the case of λ = 300 nm, a Quartz tube was used instead (diameter = 1.00 cm). No optical filters were used for those irradiation experiments. Dichloromethane was degassed using the freeze-pump-thaw method (three cycles, 2.1×10^{-3} mbar). All other solvents used for photochemical reactions were degassed with argon (15 minutes) prior to use.

Datasheet FLT021 LZC-UVA

Basic Information

Type	Fluorescent light tube
Description	Luzchem LZC-UVA
Manufacturer / Supplier	Hitachi / Luzchem
Order number / Date of purch.	LZC-UVA / 09/2015
Internal lot / serial number	2015-09 / FLT021

Specification Manufacturer

Type / size	T5 tube, G5 socket
Mechanical specification	16 mm diameter, 288 mm length
Electrical specification	8 W
Wavelength (range, typ.)	300 - 400 nm, 350m nm, UV-A
Spectral width (FWHM)	~ 40 nm
Datasheet	

Characterization

Description of measurement	Measured with Ocean-optics USB4000 spectrometer using a calibrated setup (cosine corrector/fibre). The cosine corrector was placed at 20 mm distance from a single fluorescent tube at half height.

Measured dominant wavelength / Int.	350 nm	115 µW/mm²nm
Measured spectral width (FWHM)	40 nm	
Integral Reference intensity / range	5017 µW/cm²	300-425 nm

Spectrum

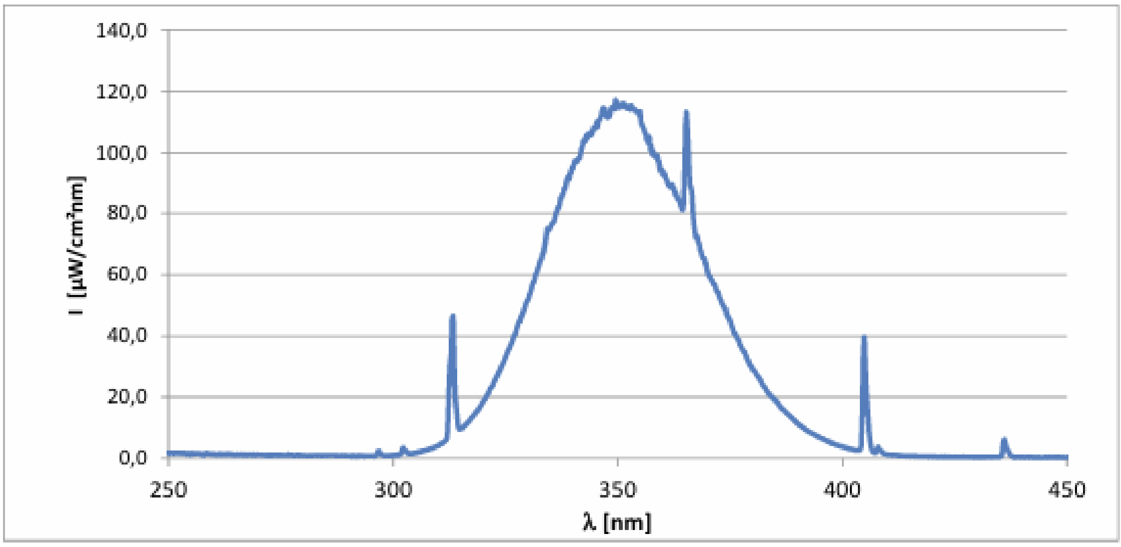

Datasheet FLT025 RPR3000

Basic Information

Type	Fluorescent light tube
Description	Luzchem Sankyo Denki, G8T5E, 300 nm
Manufacturer / Supplier	n/a / Luzchem
Order number / Date of purch.	n/a / 02/2021
Internal lot / serial number	2021-02 / FLT025

Specification Manufacturer

Type / size	T5 tube, G5 socket
Mechanical specification	16 mm diameter, 288 mm length
Electrical specification	8 W
Wavelength (range, typ.)	300 nm
Spectral width (FWHM)	~ 30 nm
Datasheet	01/2024

Characterization

Description of measurement	Measured with Ocean-optics USB4000 spectrometer using a calibrated setup (cosine corrector/fibre). The cosine corrector was placed at 20 mm distance from a single fluorescent tube at half height.	
Measured dominant wavelength / Int.	313 nm	138 µW/mm²nm
Measured spectral width (FWHM)	40 nm	
Integral Reference intensity / range	4404 µW/cm²	260-380 nm

Spectrum

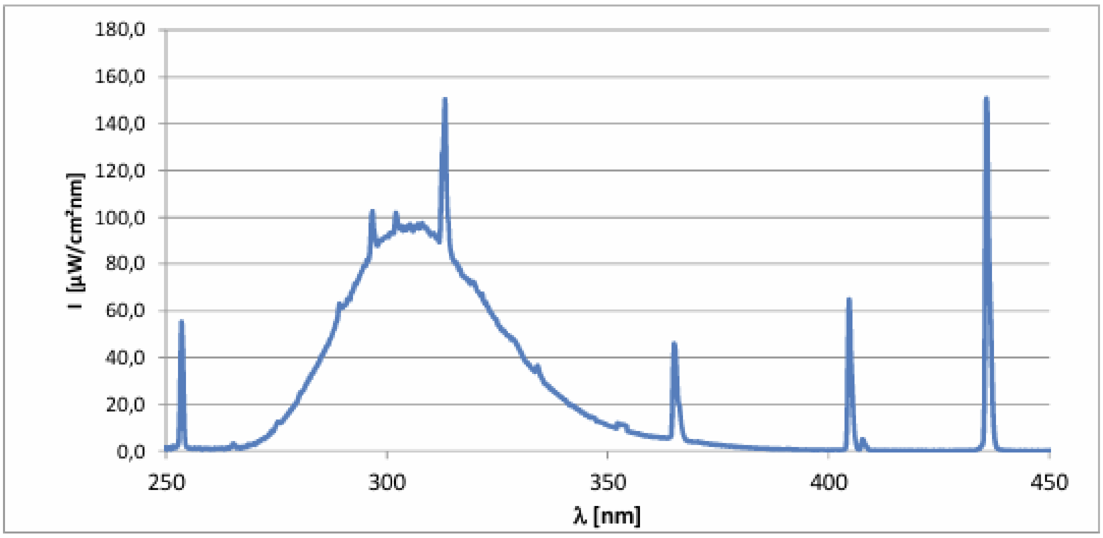

2. Allene Synthesis

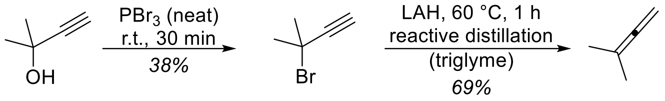

The given yields represent the average of several conducted syntheses.

Bromination:

The experimental procedure was conducted according to a previous literature report.[9] To a solution of 2-methylbut-3-yn-2-ol (23.1 mL, 20.0 g, 237 mmol, 1.00 equiv), phosphorus tribromide (7.44 mL, 21.2 g, 78.2 mmol, 0.330 equiv) was added dropwise at 0 °C over two hours. The reaction was stirred for a further 30 minutes at room temperature. The suspension was vacuum distilled with a 10 cm Vigreux column in a 60 °C oil bath (b.p.: 30°C/80 mbar) to afford 3-bromo-3-methylbut-1-yne (13.6 g, 92.6 mmol, 38%) as a colorless liquid.

Reduction:

The experimental procedure is based on a literature protocol, which was modified for this synthesis.[10] 1-Methoxy-2-[2-(2-methoxyethoxy)ethoxy]ethane (25.7 mL) was added to a two-necked round bottom flask sealed with a septum and connected to a micro distillation apparatus. Lithium aluminum hydride (1.22 g, 32.1 mmol, 0.500 equiv) was added, and the reaction was heated to 60 °C. 3-Bromo-3-methylbut-1-yne (7.45 mL, 9.45 g, 64.2 mmol, 1.00 equiv) was added dropwise. After complete addition of the starting material, the reaction was stirred for an additional 30 minutes, and 1,1-dimethylallene (3.02 g, 44.3 mmol, 69%) was subsequently isolated as a colorless liquid in the corresponding collection flask by distillation at ambient pressure.

2.1. 3-Bromo-3-methylbut-1-yne

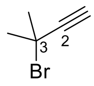

C_5H_7Br
M = 147.02 g/mol

b.p.: 30 °C (80 mbar).

^{1}H-NMR (400 MHz, $CDCl_3$): δ [ppm] = 2.71 (s, 1H, C1-H), 2.03 [s, 6H, C3(CH_3)$_2$].

^{13}C-NMR (101 MHz, $CDCl_3$): δ [ppm] = 86.4 (s, C2), 72.7 (d, C1), 49.3 (s, C3), 30.1 [q, C3(CH$_3$)$_2$].

The analytical data are in accordance with previous literature reports.[9]

2.2. 3-Methylbuta-1,2-diene

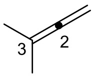

C_5H_8
M = 68.12 g/mol

b.p.: 40 °C (ambient pressure).

^{1}H-NMR (500 MHz, $CDCl_3$): δ [ppm] = 4.52 (hept, ^5J = 3.1 Hz, 2H, C2CH_2), 1.69 [t, ^5J = 3.1 Hz, 6H, C3$(CH_3)_2$].

^{13}C-NMR (101 MHz, $CDCl_3$): δ [ppm] = 206.8 (s, C2), 94.2 (s, C3), 72.7 (t, C1), 20.3 [q, C3($C$$H_3$)$_2$].

The analytical data are in accordance with previous literature reports.[10]

3. General Procedure 1 (GP1): Converting *para*-Substituted Acetophenone Derivatives with 1,1-Dimethylallene

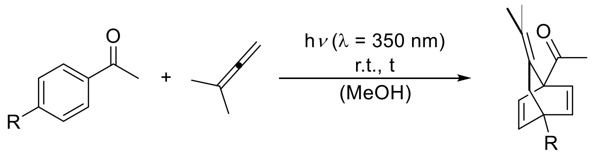

S1 **2**

To a flame-dried Duran phototube, the corresponding *para*-substituted acetophenone derivative **S1** (20.0 mg, 1.00 equiv) and 1,1-dimethylallene (30.0 equiv) were added. Methanol was added (0.01 M) and the solution was irradiated at 350 nm at room temperature until the starting material was fully converted (reaction monitoring by TLC). The solvent and the residual allene were removed under reduced pressure, and the crude product was purified by column chromatography (P/EtOAc) to yield the corresponding photoproduct **2**.

In the course of the optimization of this reaction with acetophenone as model substrate, other solvents (CH_2Cl_2 and MeCN) were tried but resulted either in a slower conversion or lower purity.

3.1. 1-[7-(Propan-2-ylidene)bicyclo[2.2.2]octa-2,5-dien-1-yl]ethan-1-one (2a)

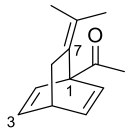

2a

$C_{13}H_{16}O$

M = 188.27 g/mol

Compound **2a** was synthesized according to GP1. As starting material, acetophenone (19.4 µL, 20.0 mg, 166 µmol, 1.00 equiv) was used and the corresponding amount of 1,1-dimethylallene (340 µL, 490 mg, 5.00 mmol, 30.0 equiv) was added. The respective amount of methanol (16.6 mL) was added, and the reaction mixture was irradiated for 14 hours. After purification by column chromatography (P/EtOAc = 30/1), product **2a** was obtained as white crystals (16.8 mg, 89.2 µmol, 54%).

TLC: R_f = 0.46 (P/EtOAc) = 15/1 [SM].

^{1}H-NMR (400 MHz, $CDCl_3$): δ [ppm] = 6.66 (dd, ^3J = 7.2 Hz, ^4J = 1.7 Hz, 2H, C2-H, C6-H), 6.48 (dd, ^3J = 7.2 Hz, ^3J = 6.0 Hz, 2H, C3-H, C5-H), 3.68 (m, 1H, C4-H), 2.52 (s, 3H, $COCH_3$), 2.00 (*virt.* dhept, ^3J = 2.6 Hz, $^5J \approx ^5J$ = 1.4 Hz, 2H, $C7CH_2$), 1.61 [t, ^5J = 1.4 Hz, 3H, $C(C\mathit{H_3}CH_3)$], 1.54 [t, ^5J = 1.4 Hz, 3H, $C(CH_3C\mathit{H_3})$].

Assignment of the doublet of doublets was done with the help of the corresponding HMBC spectrum (see section *NMR Spectra of New Compounds*), in which the protons of the acetyl group show a correlation to one of the magnetically equivalent olefinic carbon atoms at C2 and C6 (see Figure S1). All other proton assignments are based on HSQC and COSY spectra which are exemplarily shown in the spectra section (pages 38-40).

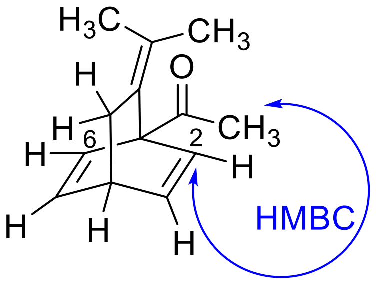

Figure S1: HMBC correlation between the acetyl group and the protons at C2 and C6 enabling the assignment of the doublet of doublets in the ^{1}H NMR spectrum of compound **2a**. HMBC contact displayed by a blue arrow.

^{13}C-NMR (101 MHz, $CDCl_3$): δ [ppm] = 209.1 (s, CO), 135.7 (d, C3, C5), 132.2 (d, C2, C6), 131.1 (s, C7*), 121.6 [s, $C(CH_3)_2$*], 64.3 (s, C1), 38.7 (d, C4), 35.5 (t, C7CH_2), 30.0 (q, OCCH_3), 24.2 [q, C(CH_3CH_3)], 21.6 [q, C(CH_3CH_3)].

*Signals are interchangable.

HRMS (ESI): $C_{13}H_{16}ONa^+$ $[M+Na]^+$ *m/z* calculated = 211.1093; found = 211.1092.

IR (ATR): ṽ [cm^{-1}] = 2932 (m, C−H), 2852 (w, C−H), 1702 (m, C=O), 1347 (m), 1255 (m, C−O), 788 (m), 713 (s, C_{alkene}), 676 (s, =C−H).

m.p.: 55°C.

3.2. 1-[4-Methyl-7-(propan-2-ylidene)bicyclo[2.2.2]octa-2,5-dien-1-yl]ethan-1-one (2b)

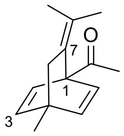

2b
$C_{14}H_{18}O$
M = 202.30 g/mol

Compound **2b** was synthesized according to GP1. As starting material, 4-methylacetophenone (19.9 µL, 20.0 mg, 149 µmol, 1.00 equiv) was used and the corresponding amount of 1,1-dimethylallene (439 µL, 305 mg, 4.47 mmol, 30.0 equiv) was added. The respective amount of methanol (14.9 mL) was added, and the reaction mixture was irradiated for 14 hours. After purification by column chromatography (P/EtOAc = 30/1), product **2b** was obtained as a colorless oil (15.1 mg, 74.4 µmol, 50%).

TLC: R_f = 0.55 (P/EtOAc) = 10/1 [SM].

^{1}H-NMR (500 MHz, $CDCl_3$): δ [ppm] = 6.64 (d, ^3J = 7.2 Hz, 2H, C2-H, C6-H), 6.20 (d, ^3J = 7.2 Hz, 2H, C3-H, C5-H), 2.52 (s, 3H, $COCH_3$), 1.90-1.87 (m, 2H, C7CH_2), 1.60 [t, ^5J = 1.7 Hz, 3H, C(CH_3CH_3)], 1.53 [t, ^5J = 1.1 Hz, 3H, C(CH_3CH_3)], 1.52 (s, 3H, C4CH_3).

^{13}C-NMR (101 MHz, $CDCl_3$): δ [ppm] = 209.8 (s, CO), 141.0 (d, C3, C5), 133.0 (s, C7*), 132.0 (d, C2, C6), 121.1 [s, $C(CH_3)_2$*], 65.0 (s, C1), 43.7 (t, C7CH_2), 43.6 (s, C4), 30.0 (q, OCCH_3), 24.2 [q, C(CH_3CH_3)], 22.1 (q, C4CH_3), 21.5 [q, C(CH_3CH_3)].

*Signals are interchangable.

Assignment of the NMR signals was done as for substrate **2a** and with the aid of the ^{1}H NMR spectrum (see section *NMR Spectra of New Compounds*) of compound **2a**.

HRMS (ESI): $C_{14}H_{19}O^+$ $[M+H]^+$ *m/z* calculated = 203.1431; found = 203.1431.

IR (ATR): ṽ [cm^{-1}] = 2924 (w, C–H), 1708 (s, C=O), 1350 (m, C–H), 1250 (w), 1113 (w), 796 (m), 709 (s, C_{alkene}), 676 (m), 640 (w).

3.3. 1-[4-(*tert*-Butyl)-7-(propan-2-ylidene)bicyclo[2.2.2]octa-2,5-dien-1-yl]ethan-1-one (2c)

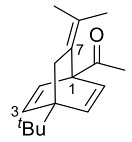

2c
$C_{17}H_{24}O$
M = 244.38 g/mol

Compound **2c** was synthesized according to GP1. As starting material, 4-*tert*-butylacetophenone (20.7 µL, 20.0 mg, 113 µmol, 1.00 equiv) was used and the corresponding amount of 1,1-dimethylallene (334 µL, 231 mg, 3.40 mmol, 30.0 equiv) was added. The respective amount of methanol (11.3 mL) was added, and the reaction mixture was irradiated for 18 hours. After purification by column chromatography (P/EtOAc = 35/1), product **2c** was obtained as yellow crystals (14.7 mg, 60.2 µmol, 53%).

TLC: R_f = 0.22 (P/EtOAc) = 35/1 [SM].

^{1}H-NMR (400 MHz, $CDCl_3$): δ [ppm] = 6.69 (d, ^{3}J = 7.6 Hz, 2H, C2-H, C6-H), 6.47 (d, ^{3}J = 7.6 Hz, 2H, C3-H, C5-H), 2.51 (s, 3H, $COCH_3$), 1.98-1.95 (m, 2H, $C7CH_2$), 1.60 [t, ^{5}J = 1.7 Hz, 3H, $C(CH_3CH_3)$], 1.55 [t, ^{5}J = 1.1 Hz, 3H, $C(CH_3CH_3)$], 1.1 [s, 9H, $C(CH_3)_3$].

^{13}C-NMR (101 MHz, $CDCl_3$): δ [ppm] = 209.8 (s, CO), 137.3 (d, C3, C5), 133.5 (s, C7*), 132.4 (d, C2, C6), 120.7 [s, $C(CH_3)_2$*], 64.1 (s, C1), 54.8 (s, C4), 36.5 (t, $C7CH_2$), 32.2 [s, $C(CH_3)_3$], 30.0 (q, $OCCH_3$), 26.5 [q, $C(CH_3)_3$], 24.1 [q, $C(CH_3CH_3)$], 21.6 [q, $C(CH_3CH_3)$].

*Signals are interchangable.

Assignment of the NMR signals was done as for substrate **2a** and with the aid of the ^{1}H NMR spectrum (see section *NMR Spectra of New Compounds*) of compound **2a**.

HRMS (ESI): $C_{17}H_{24}ONa^{+}$ $[M+Na]^{+}$ *m/z* calculated = 267.1719; found = 267.1719.

IR (ATR): ṽ [cm^{-1}] = 2957 (w, C−H), 1710 (s, C=O), 1349 (m), 1253 (m), 1076 (m), 791 (m), 705 (s, C_{alkene}), 680 (m).

m.p.: 66 °C - 69 °C.

3.4. 1-[4-Methoxy-7-(propan-2-ylidene)bicyclo[2.2.2]octa-2,5-dien-1-yl]ethan-1-one (2d)

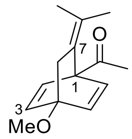

2d
$C_{14}H_{18}O_2$
M = 218.30 g/mol

Compound **2d** was synthesized according to GP1. As starting material, 4-methoxyacetophenone (20.0 mg, 133 µmol, 1.00 equiv) was used and the corresponding amount of 1,1-dimethylallene (392 µL, 272 mg, 4.00 mmol, 30.0 equiv) was added. The respective amount of methanol (13.3 mL) was added, and the reaction mixture was irradiated for 19 hours. After purification by column chromatography (P/EtOAc = 30/1 → 25/1), product **2d** was obtained as orange crystals (17.0 mg, 77.9 µmol, 58%).

TLC: R_f = 0.45 (P/EtOAc) = 10/1 [SM].

^{1}H-NMR (400 MHz, $CDCl_3$): *δ* [ppm] = 6.62 (d, ^{3}J = 7.9 Hz, 2H, C2-H, C6-H), 6.56 (d, ^{3}J = 7.9 Hz, 2H, C3-H, C5-H), 3.57 (s, 3H, OCH_3), 2.51 (s, 3H, $COCH_3$), 2.19-2.17 (m, 2H, C7CH_2), 1.59 [t, ^{5}J = 1.7 Hz, 3H, C(C$H_3$$CH_3$)], 1.54 [t, ^{5}J = 1.1 Hz, 3H, C(CH_3CH_3)].

^{13}C-NMR (101 MHz, $CDCl_3$): *δ* [ppm] = 208.8 (s, CO), 136.5 (d, C3, C5), 130.4 (d, C2, C6), 129.4 (s, C7*), 122.4 [s, *C*(CH_3)$_2$*], 85.0 (s, C4), 63.9 (s, C1), 53.3 (q, OCH_3), 40.5 (t, C7*C*H_2), 29.9 (q, OC*C*H_3), 24.2 [q, C(*C*$H_3$$CH_3$)], 21.3 [q, C($CH_3$*C*$H_3$)].

*Signals are interchangable.

Assignment of the NMR signals was done as for substrate **2a** and with the aid of the ^{1}H NMR spectrum (see section *NMR Spectra of New Compounds*) of compound **2a**.

HRMS (ESI): $C_{14}H_{19}O_2^+$ [M+H]$^+$ *m/z* calculated = 219.1380; found = 219.1380.

IR (ATR): ṽ [cm^{-1}] = 2917 (w, C–H), 1706 (s, C=O), 1348 (m), 1174 (m), 1111 (s, C–O), 1089 (s, C–O), 712 (s, C_{alkene}), 677 (m).

m.p.: 42 °C.

4. General Procedure 2 (GP2): Synthesis of Bicyclic Nucleophile Addition Products in Alcohols as the Solvent

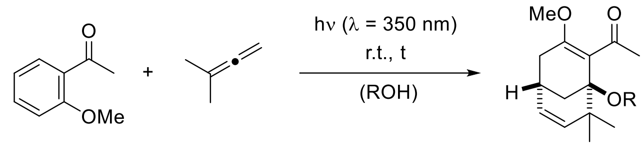

3 4

To a flame-dried Duran phototube, 2-methoxyacetophenone (**3**, 18.4 µL, 20.0 mg, 133 µmol, 1.00 equiv) and the alcoholic solvent (13.3 mL) were added. 1,1-Dimethylallene (392 µL, 272 mg, 4.00 mmol, 30.0 equiv) was added and the solution was irradiated at 350 nm at room temperature until the starting material was fully converted (monitored by TLC). The solvent and the residual allene were removed under reduced pressure, and the crude product was purified by column chromatography (P/EtOAc) to yield the respective bicyclic photoproduct **4**.

4.1. 1-[(1*S*,5*S*)-1,3-Dimethoxy-8,8-dimethylbicyclo[3.3.1]nona-2,6-dien-2-yl]ethan-1-one (4a)

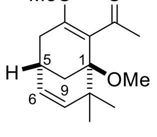

4a
$C_{15}H_{22}O_3$
M = 250.34 g/mol

Compound **4a** was synthesized according to GP2. As solvent, methanol was used, and the solution was irradiated for three hours. After purification by column chromatography (P/EtOAc = 25/1 → 8/1), product **4a** was obtained as a white powder (19.1 mg, 76.2 µmol, 57%).

TLC: R_f = 0.29 (P/EtOAc) = 10/1 [SM].

^{1}H-NMR (400 MHz, $CDCl_3$): δ [ppm] = 5.50 (dd, ^3J = 9.7 Hz, ^3J = 4.9 Hz, 1H, C6-H), 5.34 (dd, ^3J = 9.7 Hz, ^4J = 0.6 Hz, 1H, C7-H), 3.60 (s, 3H, $C3OCH_3$), 3.25 (s, 3H, $C1OCH_3$), 2.71 (m, 1H, C5-H), 2.46 (dd, ^2J = 17.3 Hz, ^3J = 5.8 Hz, 1H, C4-*H*H), 2.30 (s, 3H, $COCH_3$), 2.22 (dd, ^2J = 17.3 Hz, ^3J = 1.0 Hz, 1H, C4-H*H*), 1.83-1.80 (m, 2H, $C1CH_2$), 1.04 [s, 3H, $C8(CH_3CH_3)$], 0.94 [s, 3H, $C8(CH_3CH_3)$].

^{13}C-NMR (101 MHz, $CDCl_3$): δ [ppm] = 202.4 (s, CO), 159.6 (s, C3), 139.2 (d, C7), 125.3 (d, C6), 121.6 (s, C2), 81.1 (s, C1), 55.3 (q, $C3OCH_3$), 51.3 (q, $C1OCH_3$), 42.9 (s, C8), 33.5 (q, $OCCH_3$), 31.5 (t, C4), 30.0 (d, C5), 28.9 (t, C9), 25.9 [q, $C8(CH_3CH_3)$], 25.3 [q, $C8(CH_3CH_3)$].

Assignment of the NMR signals was done as for substrate **4b** and with the aid of the ^{1}H NMR spectrum (see section *NMR Spectra of New Compounds*) of compound **4b**.

HRMS (ESI): $C_{15}H_{23}O_3^+$ [M+H]$^+$ *m/z* calculated = 251.1641; found = 251.1641.

IR (ATR): ṽ [cm^{-1}] = 2923 (w, C–H), 1672 (s, C=O), 1610 (s, C=O), 1363 (m, C–H), 1363 (m, C–H), 1283 (m), 1198 (m), 1161 (m), 1093 (s, C–O), 1069 (s, C–O), 772 (m, C_{alkene}), 752 (m, C_{alkene}).

m.p.: 90 °C.

4.2. 1-[(1*S*,5*S*)-1-Ethoxy-3-methoxy-8,8-dimethylbicyclo[3.3.1]nona-2,6-dien-2-yl]ethan-1-one (4b)

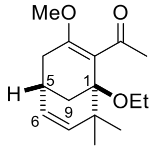

4b
$C_{16}H_{24}O_3$
M = 264.37 g/mol

Compound **4b** was synthesized according to GP2. As solvent, ethanol was used and the solution was irradiated for three hours. After purification by column chromatography (P/EtOAc = 30/1), product **4b** was obtained as white crystals (26.1 mg, 98.7 µmol, 74%).

TLC: R_f = 0.30 (P/EtOAc) = 10/1 [SM].

^{1}**H-NMR** (400 MHz, $CDCl_3$): *δ* [ppm] = 5.49 (ddd, ^{3}J = 9.6 Hz, ^{3}J = 5.3 Hz, ^{4}J = 1.4 Hz, 1H, C6-H), 5.35 (dd, ^{3}J = 9.6 Hz, ^{4}J = 0.7 Hz, 1H, C7-H), 3.58 (s, 3H, OCH_3), 3.53-3.39 (m, 2H, C*H*$_2$CH$_3$), 2.72-2.67 (m, 1H, C5-H), 2.44 (dd, ^{2}J = 17.3 Hz, ^{3}J = 5.9 Hz, 1H, C4-*H*H), 2.29 (s, 3H, $COCH_3$), 2.19 (*virt.* dt, ^{2}J = 17.3 Hz, $^{3}J \approx {}^{4}J$ = 1.5 Hz, 1H, C4-H*H*), 1.88 (ddd, ^{2}J = 11.9 Hz, ^{3}J = 4.4 Hz, ^{4}J = 1.4 Hz, 1H, C9-*H*H), 1.79 (ddd, ^{2}J = 11.9 Hz, ^{3}J = 2.4 Hz, ^{4}J = 1.5 Hz, 1H, C9-H*H*), 1.18 (t, ^{3}J = 6.9 Hz, 3H, CH$_2$C*H*$_3$), 1.12 [s, 3H, C8(C*H*$_3$CH$_3$)], 1.04 [s, 3H, C8(CH$_3$C*H*$_3$)].

Assignment of the olefinic protons was done with the help of the corresponding COSY spectrum (see section *NMR Spectra of New Compounds*), in which only one of the olefinic protons shows a coupling to a proton at carbon atom C9 (see Figure S2). The coupling allowed to assign the proton at C6. This also helps to assign the methylene protons of the bicyclic core of compound **4b** to the corresponding multiplets between 2.44 ppm and 1.79 ppm. All assignments were confirmed by the corresponding HMBC signals.

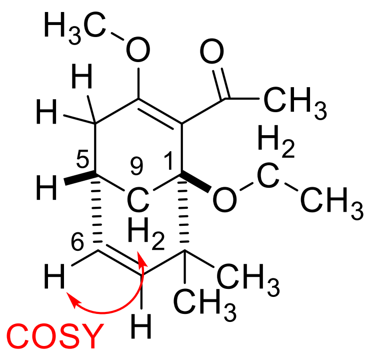

Figure S2: Coupling between the olefinic proton at C6 and a proton at carbon atom C9 in the COSY spectrum of compound **4b**. This coupling enables the assignment of the olefinic protons and thus, the assignment of all the multiplets between 2.44 ppm and 1.79 ppm. COSY contact displayed by a red arrow.

^{13}**C-NMR** (126 MHz, $CDCl_3$): *δ* [ppm] = 202.8 (s, CO), 158.1 (s, C3), 139.2 (d, C7), 125.3 (d, C6), 122.8 (s, C2), 80.4 (s, C1), 58.4 (t, *C*H$_2$CH$_3$), 55.4 (q, O*C*H$_3$), 42.8 (s, C8), 33.5 (q, OC*C*H$_3$), 31.4 (t, C4), 30.1 (d, C5), 29.7 (t, C9), 26.1 [q, C8(*C*H$_3$CH$_3$)], 25.2 [q, C8(CH$_3$*C*H$_3$)].

HRMS (ESI): $C_{16}H_{25}O_3^+$ [M+H]$^+$ *m/z* calculated = 265.1798; found = 265.1798.

IR (ATR): $\tilde{\nu}$ [cm^{-1}] = 2969 (w, C−H), 2926 (w, C−H), 2905 (w, C−H), 1675 (s, C=O), 1361 (m, C−H), 1147 (s, C−O), 1091 (s, C−O), 1031 (s, C−O), 771 (m, C_{alkene}).

m.p.: 45 °C.

4.3. 1-[(1*S*,5*S*)-1-Isopropoxy-3-methoxy-8,8-dimethylbicyclo[3.3.1]nona-2,6-dien-2-yl]ethan-1-one (4c)

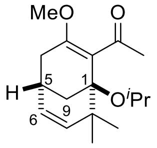

4c
$C_{17}H_{26}O_3$
M = 278.39 g/mol

Compound **4c** was synthesized according to GP2. As solvent, isopropanol was used and the solution was irradiated for three hours. After purification by column chromatography (P/EtOAc = 25/1), product **4c** was obtained as a colorless oil (26.2 mg, 94.2 µmol, 71%).

TLC: R_f = 0.30 (P/EtOAc) = 10/1 [SM].

^{1}H-NMR (400 MHz, $CDCl_3$): δ [ppm] = 5.45 (ddd, ^3J = 9.7 Hz, ^3J = 4.8 Hz, ^4J = 1.4 Hz, 1H, C6-H), 5.33 (d, ^3J = 9.7 Hz, 1H, C7-H), 3.84 [hept, ^3J = 5.9 Hz, 1H, *H*C(CH_3CH_3)], 3.55 (s, 3H, OCH_3), 2.71 (*virt.* dtt, ^3J = 6.3 Hz, $^3J \approx {}^3J$ = 4.8 Hz, $^3J \approx {}^3J$ = 2.4 Hz, 1H, C5-H), 2.45 (dd, ^2J = 17.1 Hz, ^3J = 6.3 Hz, 1H, C4-*H*H), 2.31 (s, 3H, $COCH_3$), 2.16-2.07 (m, 2H, C4-H*H*, C9-*H*H), 1.86 (ddd, ^2J = 11.6 Hz, ^3J = 2.4 Hz, ^4J = 1.4 Hz, 1H, C9-H*H*), 1.16 [d, ^3J = 5.9 Hz, 3H, HC(C*H*$_3$CH$_3$)], 1.12 [d, ^3J = 5.9 Hz, 3H, HC(CH$_3$C*H*$_3$)], 1.05 [s, 3H, C8(C*H*$_3$CH$_3$)], 1.04 [s, 3H, C8(CH$_3$C*H*$_3$)].

^{13}C-NMR (101 MHz, $CDCl_3$): δ [ppm] = 203.0 (s, CO), 156.6 (s, C3), 139.3 (d, C7), 125.4 (d, C6), 125.0 (s, C2), 80.8 (s, C1), 64.7 [d, H*C*(CH_3CH_3)], 55.2 (q, O*C*H$_3$), 43.1 (s, C8), 33.9 (q, OC*C*H$_3$), 31.8 (t, C9), 31.0 (t, C4), 30.4 (d, C5), 26.3 [q, C8(*C*H$_3$CH$_3$)], 26.1 [q, C8(CH$_3$*C*H$_3$)], 25.5 [q, HC(*C*H$_3$CH$_3$)], 24.9 [q, HC(CH$_3$*C*H$_3$)].

Assignment of the NMR signals was done as for substrate **4b** and with the aid of the ^{1}H NMR spectrum (see section *NMR Spectra of New Compounds*) of compound **4b**.

HRMS (ESI): $C_{17}H_{27}O_3^+$ $[M+H]^+$ *m/z* calculated = 279.1955; found = 279.1954.

IR (ATR): $\tilde{\nu}$ [cm^{-1}] = 2964 (w, C−H), 2923 (w, C−H), 1687 (m, C=O), 1364 (m, C−H), 1085 (s, C−O), 1069 (s, C−O), 1039 (s, C−O), 770 (m, C_{alkene}), 746 (m, C_{alkene}).

4.4. 1-{(1*S*,5*S*)-1-[(2,4-Dimethylpentan-3-yl)oxy]-3-methoxy-8,8-dimethylbicyclo[3.3.1]-nona-2,6-dien-2-yl}ethan-1-one (4d)

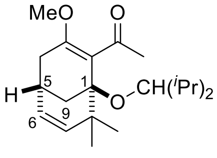

4d
$C_{21}H_{34}O_3$
M = 334.50 g/mol

Compound **4d** was synthesized according to GP2. As solvent, DM-3-P was used and the solution was irradiated for 16 hours. After purification by column chromatography (P/EtOAc = 20/1), product **4d** was obtained as a yellow oil (26.2 mg, 78.3 µmol, 59%).

TLC: R_f = 0.51 (P/EtOAc) = 5/1 [SM].

^{1}H-NMR (400 MHz, $CDCl_3$): δ [ppm] = 5.43 (ddd, ^3J = 9.9 Hz, ^3J = 5.2 Hz, ^4J = 1.5 Hz, 1H, C6-H), 5.31 (d, ^3J = 9.9 Hz, 1H, C7-H), 3.58 (s, 3H, OCH_3), 3.31 [*virt.* t, $^3J \approx {}^3J$ = 2.8 Hz, 1H, *H*C[[HC(CH_3CH_3)][HC(CH_3CH_3)]]], 2.75-2.70 (m, 1H, C5-H), 2.15 (dd, ^2J = 17.1 Hz, ^3J = 6.1 Hz, 1H, C4-*H*H), 2.31 (s, 3H, $COCH_3$), 2.17-2.10 (m, 2H, C4-H*H*, C9-*H*H), 1.97-1.82 ((m, 2H, HC{[*H*C(CH_3CH_3)][*H*C(CH_3CH_3)]})), 1.76 (ddd, ^2J = 11.2 Hz, ^3J = 2.3 Hz, ^4J = 1.5 Hz, 1H, C9-H*H*), 1.12 [s, 3H, C8(C$H_3$$CH_3$)], 1.10 [s, 3H, C8($CH_3CH_3$)], 0.97-0.93 ((m, 6H, HC{[HC(C$H_3$$CH_3$)][HC(C$H_3$$CH_3$)]})), 0.91 ((d, ^3J = 7.1 Hz, 3H, HC{[HC(CH_3CH_3)][HC(CH_3CH_3)]})), 0.87 ((d, ^3J = 7.1 Hz, 3H, HC{[HC(CH_3CH_3)][HC(CH_3CH_3)]})).

^{13}C-NMR (101 MHz, $CDCl_3$): δ [ppm] = 201.5 (s, CO), 158.9 (s, C3), 139.9 (d, C7), 125.3 (d, C6), 124.6 (s, C2), 80.6 (s, C1), 80.4 [d, H*C*[[HC(CH_3CH_3)][HC(CH_3CH_3)]]], 55.2 (q, O*C*H_3), 44.4 (s, C8), 34.5 (q, OC*C*H_3), 32.5 (t, C9), 31.9 (t, C4), 31.7 [d, HC[[H*C*(CH_3CH_3)][HC(CH_3CH_3)]]], 30.8 (d, C5), 30.4 [d, HC[[HC(CH_3CH_3)][H*C*(CH_3CH_3)]]], 27.6 [q, C8(*C*$H_3$$CH_3$)], 27.0 [q, C8($CH_3$*C*$H_3$)], 21.2 [q, HC[[HC(*C*$H_3$$CH_3$)][HC($CH_3CH_3$)]]], 19.9 [q, HC[[HC($CH_3$*C*$H_3$)][HC($CH_3CH_3$)]]], 19.3 [q, HC[[HC($CH_3CH_3$)][HC(*C*$H_3$$CH_3$)]]], 18.5 [q, HC[[HC($CH_3CH_3$)][HC($CH_3$*C*$H_3$)]]].

Assignment of the NMR signals was done as for substrate **4b** and with the aid of the ^{1}H NMR spectrum (see section *NMR Spectra of New Compounds*) of compound **4b**.

HRMS (ESI): $C_{21}H_{35}O_3^+$ $[M+H]^+$ *m/z* calculated = 335.2581; found = 335.2580.

IR (ATR): $\tilde{\nu}$ [cm^{-1}] = 2925 (m, C–H), 1682 (m, C=O), 1463 (m), 1367 (m, C–H), 1070 (s, C–O), 1038 (s, C–O), 751 (m, C_{alkene}).

5. General Procedure 3 (GP3): Synthesis of Bicyclic Nucleophile Addition Products in Dichloromethane as Solvent

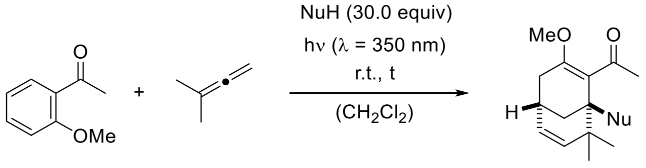

3 4, 10

To a flame-dried Duran phototube, the respective nucleophile (4.00 mmol, 30.0 equiv) was added, and the phototube was evacuated and flushed with argon for three times. 2-Methoxyacetophenone (**3**, 18.4 µL, 20.0 mg, 133 µmol, 1.00 equiv), 1,1-dimethylallene (392 µL, 272 mg, 4.00 mmol, 30.0 equiv), and dichloromethane (13.3 mL) were added. The solution was irradiated until the starting material was fully converted (monitored by TLC), and the solvent and the residual allene were removed under reduced pressure. The crude product was purified by column chromatography to yield the products.

5.1. 1-{(1*S*,5*S*)-1-[(4',4'-Dimethylcyclohexyl)oxy]-3-methoxy-8,8-dimethylbicyclo[3.3.1]nona-2,6-dien-2-yl}ethan-1-one (4e)

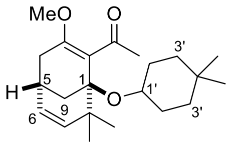

4e
$C_{22}H_{34}O_3$
M = 346.51 g/mol

Compound **4e** was synthesized according to GP3. As nucleophile, 4,4-dimethylcyclohexanol (438 mg, 4.00 mmol, 30.0 equiv) was used and the solution was irradiated for six hours. After purification by column chromatography (P/EtOAc = 20/1) product **4e** was obtained as white crystals (23.3 mg, 67.2 µmol, 59%).

TLC: R_f = 0.44 (P/EtOAc) = 10/1 [SM].

^{1}H-NMR (400 MHz, $CDCl_3$): δ [ppm] = 5.45 (ddd, ^3J = 9.7 Hz, ^3J = 5.3 Hz, ^4J = 1.6 Hz, 1H, C6-H), 5.32 (d, ^3J = 9.7 Hz, 1H, C7-H), 3.56 (s, 3H, OCH_3), 3.45 (tt, ^3J = 8.5 Hz, ^3J = 3.7 Hz, 1H, C1'-H), 2.74-2.67 (m, 1H, C5-H), 2.46 (dd, ^2J = 17.0 Hz, ^3J = 6.0 Hz, 1H, C4-*H*H), 2.30 (s, 3H, $COCH_3$), 2.13 (*virt.* dt, ^2J = 17.0 Hz, ^3J = 1.4 Hz ≈ ^4J = 1.4 Hz, 1H, C4-H*H*), 2.07 (dd, ^2J = 11.6 Hz, ^3J = 4.5 Hz, 1H, C9-*H*H), 1.79 (*virt.* dt, ^2J = 11.6 Hz, ^3J ≈ ^4J = 1.6 Hz, 1H, C9-H*H*), 1.75-1.35 [m, 6H, C1'$(CH_2)_2$, C3'-*H*H], 1.15 (ddd, ^2J = 14.4 Hz, ^3J = 10.9 Hz, ^3J = 4.0 Hz, 2H, C3'-H*H*), 1.07 [s, 3H, C8($C\mathit{H_3}CH_3$)], 1.05 [s, 3H, C8($CH_3C\mathit{H_3}$)], 0.90 [s, 3H, C4'($C\mathit{H_3}CH_3$)], 0.87 [s, 3H, C4'($CH_3C\mathit{H_3}$)].

^{13}C-NMR (101 MHz, $CDCl_3$): δ [ppm] = 202.6 (s, CO), 157.3 (s, C3), 139.4 (d, C7), 125.3 (s, C2), 125.0 (d, C6), 81.1 (s, C1), 70.9 (d, C1'), 55.3 (q, OCH_3), 43.3 (s, C8), 37.3 (t, C3'), 37.3 (t, C3'), 34.0 (q, OC*C*H_3), 32.3 (t, C9), 31.3 (t, C4), 31.3 (t, C2'), 31.9 [s, *C*$(CH_3)_3$], 30.5 (d, C5), 30.1 [q, C4'(*C*H_3CH_3)], 29.9 [q, *C4'*(CH_3CH_3)], 29.7 (t, C2'), 26.8 [q, C4'(CH_3*C*H_3), C8(*C*H_3CH_3)], 26.4 [q, C8(CH_3*C*H_3)].

Assignment of the NMR signals was done as for substrate **4b** and with the aid of the ^{1}H NMR spectrum (see section *NMR Spectra of New Compounds*) of compound **4b**.

HRMS (ESI): $C_{22}H_{35}O_3^+$ $[M+H]^+$ *m/z* calculated = 347.2581; found = 347.2582.

IR (ATR): $\tilde{\nu}$ [cm^{-1}] = 2926 (m, C−H), 1679 (m, C=O), 1606 (w), 1461 (w, C−H), 1367 (m, C−H), 1147 (m), 1069 (s, C−O), 1037 (s, C−O), 761 (s, C_{alkene}).

m.p.: 76 °C - 79 °C.

5.2. (+)-Menthol Addition Products

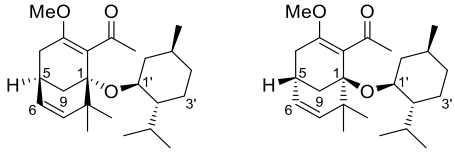

4f
$C_{26}H_{42}O_3$
M = 402.62 g/mol
d.r. = 62/38

Compounds **4f** were synthesized according to GP3. As nucleophile, (+)-menthol (624 mg, 4.00 mmol, 30.0 equiv) was used and the solution was irradiated for eleven hours. After purification by column chromatography ($PhCH_3$/EtOAc = 80/1) products **4f** (d.r. = 62/38) were obtained as a colorless oil (17.7 mg, 47.3 µmol, 36% and 10.8 mg, 29.3 µmol, 22%).

To help with the assignment of the diastereomers to the respective set of spectra, conformational searches using the CREST tool were performed for both diastereomers. Of the structures obtained within a 4 kcal/mol window, the NOE contact observed in NMR spectra of the major diastereomer was visible in 20 out of 23 structures of **4f’** (see below) and only in 23 out of 56 for **4f’’**. After re-optimization at the triple-zeta level of theory including an implicit solvent model for chloroform, both of the lowest energy structures (ΔG < 1 kcal/mol) for **4f’** showed a close proximity of the protons on the methyl group of the bicyclic core and the isopropyl residue on the (+)-menthol ring (Figure S3). While this was also the case in the lowest energy structure of **4f’’**, the three other structures within 1 kcal/mol showed no possibility for the observation of an NOE contact. Therefore, diastereomer **4f’** was assigned to the isolated fraction in which the NOE contact was observed, tentatively assigning it as the major diastereomer.

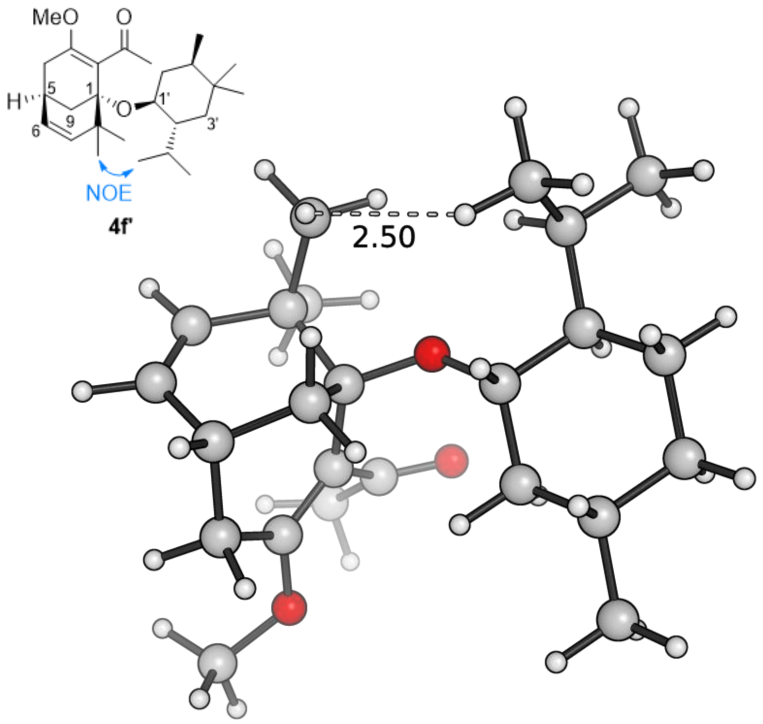

Figure S3: DFT-calculated structure of the major diastereomer **4f’** in accordance with the NOE contact only observed in **4f’**. The atomic distance is given in Å.

*5.2.1. 1-(((1S,5S)-1-{[(1S,2S,5S)-2-Isopropyl-5-methylcyclohexyl]oxy}-3-methoxy-8,8-dimethylbicyclo[3.3.1]nona-2,6-dien-2-yl))ethan-1-one (**4f'**)*

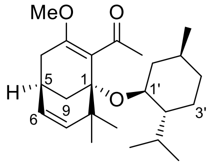

4f'
$C_{24}H_{38}O_3$
M = 374.57 g/mol

TLC: R_f = 0.51 (P/EtOAc) = 5/1 [SM].

^{1}H-NMR (500 MHz, $CDCl_3$): δ [ppm] = 5.46 (ddd, ^3J = 9.8 Hz, ^3J = 5.2 Hz, ^4J = 1.4 Hz, 1H, C6-H), 5.32 (d, ^3J = 9.8 Hz, 1H, C7-H), 3.57 (s, 3H, OCH_3), 3.44 (*virt.* td, $^3J \approx {}^3J$ = 10.1 Hz, ^3J = 3.4 Hz, 1H, C1'-H), 2.74-2.69 (m, 1H, C5-H), 2.49-2.38 [m, 2H, C4-*H*H, C2'C*H*(CH_3CH_3)], 2.29 (s, 3H, $COCH_3$), 2.19 (*virt.* dt, ^2J = 17.4 Hz, $^3J \approx {}^4J$ = 1.6 Hz, 1H, C4-H*H*), 2.03 (ddd, ^2J = 11.9 Hz, ^3J = 4.5 Hz, ^4J = 1.4 Hz, 1H, C9-*H*H), 1.99-1.91 (m, 2H, C9-H*H*, C6'-*H*H), 1.68-1.56 (m, 2H, C3'-*H*H, C4'-*H*H), 1.44 (*virt.* ddt, ^3J = 15.4 Hz, ^3J = 9.8 Hz, $^3J \approx {}^3J$ = 3.4 Hz, 1H, C2'-H), 1.35-1.25 (m, 2H, C5'-H, C6'-H*H*), 1.04 [s, 3H, C8(C*H*$_3$$CH_3$)], 0.99-0.95 [m, 4H, C8(CH_3C*H*$_3$), C3'-H*H*], 0.88-0.83 [m, 7H, C2'CH(C*H*$_3$$CH_3$), C5'$CH_3$, C4'-H*H*], 0.69 [d, ^3J = 6.9 Hz, 3H, C2'CH(CH_3C*H*$_3$)].

^{13}C-NMR (101 MHz, $CDCl_3$): δ [ppm] = 201.4 (s, CO), 158.1 (s, C3), 139.7 (d, C7), 125.9 (s, C2), 125.0 (d, C6), 78.6 (s, C1), 73.0 (d, C1'), 55.5 (q, OCH_3), 48.5 (d, C2'), 44.4 (s, C8), 41.8 (t, C6'), 43.8 (t, C4'), 34.5 (q, OC*C*H$_3$), 32.0 (t, C4), 31.6 (d, C5'), 30.7 (d, C5), 29.5 (t, C9), 26.7 [q, C8(*C*H$_3$$CH_3$)], 25.8 [q, C8($CH_3$*C*H$_3$), 24.9 [d, C2'*C*H($CH_3CH_3$)], 22.8 (t, C3'), 22.7 (q, C5'*C*H$_3$), 21.3 [q, C2'CH(*C*H$_3$$CH_3$)], 15.5 [q, C2'CH($CH_3$*C*H$_3$)].

Assignment of the NMR signals was done as for substrate **4b** and with the aid of the ^{1}H NMR spectrum (see section *NMR Spectra of New Compounds*) of compound **4b**.

HRMS (ESI): $C_{24}H_{39}O_3^+$ $[M+H]^+$ *m/z* calculated = 375.2894; found = 375.2893.

IR (ATR): $\tilde{\nu}$ [cm^{-1}] = 2924 (m, C−H), 1691 (w, C=O), 1366 (m, C−H), 1215 (m), 1154 (m), 1068 (s, C−O), 1043 (s, C−O), 737 (s, C_{alkene}).

Optical rotation: $[\alpha]_D^{25}$ = +5.0 (*c* = 1.00, CH_2Cl_2).

*5.2.2. 1-(((1R,5R)-1-{[(1S,2S,5S)-2-Isopropyl-5-methylcyclohexyl]oxy}-3-methoxy-8,8-dimethylbicyclo[3.3.1]nona-2,6-dien-2-yl))ethan-1-one (**4f''**)*

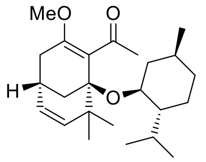

4f''
$C_{24}H_{38}O_3$
M = 374.57 g/mol

TLC: R_f = 0.54 (P/EtOAc) = 5/1 [SM].

^{1}H-NMR (500 MHz, $CDCl_3$): δ [ppm] = 5.40 (ddd, ^3J = 9.7 Hz, ^3J = 5.1 Hz, ^4J = 1.8 Hz, 1H, C6-H), 5.33 (d, ^3J = 9.7 Hz, 1H, C7-H), 3.55-3.49 (m, 4H, OCH_3, C1'-H), 2.73-2.65 (m, 1H, C5-H), 2.44 [dd, ^2J = 17.0 Hz, ^3J = 5.9 Hz, 1H, C4-*H*H], 2.33-2.27 [m, 4H, $COCH_3$, C2'C*H*(CH_3CH_3)], 2.19-2.10 (m, 3H, C4-H*H*, C9-*H*H, C6'-*H*H), 1.80 (*virt.* dt, ^2J = 11.8 Hz, $^3J \approx {}^4J$ = 1,8 Hz, 1H, C9-H*H*), 1.65-1.58 (m, 2H, C3'-*H*H, C4'-*H*H), 1.37-1.27 (m, 1H, C5'-H), 1.23-1.17 (m, 1H, C2'-H), 1.09 [s, 3H, C8(C$H_3$$CH_3$)], 1.06 [s, 3H, C8($CH_3CH_3$)], 1.04-1.01 [m, 1H, C6'-H*H*), 0.99-0.92 [m, 1H, C3'-H*H*), 0.90-0.88 [m, 6H, C5'CH_3, C2'CH(C$H_3$$CH_3$)], 0.84-0.80 [m, 1H, C4'-H*H*), 0.78 [d, ^3J = 6.9 Hz, 3H, C2'CH(CH_3CH_3)].

^{13}C-NMR (101 MHz, $CDCl_3$): δ [ppm] = 203.4 (s, CO), 156.3 (s, C3), 140.1 (d, C7), 126.1 (s, C2), 124.5 (d, C6), 81.6 (s, C1), 74.2 (d, C1'), 55.8 (q, OCH_3), 50.4 (d, C2'), 44.6 (t, C6'), 43.8 (t, C4'), 34.5 (q, OC*C*H_3), 34.3 (s, C8), 34.3 (s, C4'), 32.8 (t, C9), 32.1 (d, C5'), 31.6 (t, C4), 31.4 (d, C5), 27.6 [q, C8(*C*$H_3$$CH_3$)], 26.1 [q, C8($CH_3$*C*$H_3$), 25.3 [d, C2'*C*H($CH_3CH_3$)], 23.2 (t, C3'), 22.7 (q, C5'*C*H_3), 21.9 [q, C2'CH(*C*$H_3$$CH_3$)], 17.0 [q, C2'CH($CH_3$*C*$H_3$)].

Assignment of the NMR signals was done as for substrate **4b** and with the aid of the ^{1}H NMR spectrum (see section *NMR Spectra of New Compounds*) of compound **4b** and **4f'**.

HRMS (ESI): $C_{24}H_{38}O_3Na^+$ $[M+Na]^+$ *m/z* calculated = 397.2713; found = 397.2712.

IR (ATR): ṽ [cm^{-1}] = 2959 (m, C–H), 1681 (s, C=O), 1364 (m, C–H), 1282 (m), 1149 (m), 1078 (s, C–O), 1041 (s, C–O), 991 (m), 770 (s, C_{alkene}).

Optical rotation: $[\alpha]_D^{25}$ = +18.0 (*c* = 1.00, CH_2Cl_2).

5.3. 1-{(1*S*,5*S*)-1-[(Adamantan-1'-yl)thio]-3-methoxy-8,8-dimethylbicyclo[3.3.1]nona-2,6-dien-2-yl}ethan-1-one (10)

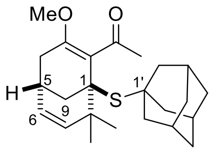

10
$C_{24}H_{34}O_2S$
M = 386.59 g/mol

Compound **10** was synthesized according to GP3. As nucleophile, 1-adamantanethiol (672 mg, 4.00 mmol, 30.0 equiv) was used and the solution was irradiated for 22 hours. After purification by column chromatography (P/EtOAc = 15/1) product **10** was obtained as a yellow powder (14.7 mg, 38.2 µmol, 29%).

TLC: R_f = 0.59 (P/EtOAc) = 5/1 [SM].

^{1}H-NMR (400 MHz, $CDCl_3$): δ [ppm] = 5.47 (ddd, ^3J = 9.8 Hz, ^3J = 4.8 Hz, ^4J = 1.5 Hz, 1H, C6-H), 5.33 (dd, ^3J = 9.8 Hz, ^4J = 0.8 Hz, 1H, C7-H), 3.54 (s, 3H, OCH_3), 2.56 (*virt.* tdt, $^3J \approx {}^3J$ = 7.7 Hz, ^3J = 4.8 Hz, $^3J \approx {}^3J$ = 2.6 Hz, 1H, C5-H), 2.47-2.37 (m, 5H, $COCH_3$, C9-*H*H, C4-*H*H), 2.19-1.96 (m, 11H, C9-H*H*, C4-H*H*, C1'C*H₂*C*H*CH₂), 1.73-1.62 (m, 6H, C1'CH₂CHC*H₂*), 1.21 [s, 3H, C8(C*H₃*CH₃)], 1.02 [s, 3H, C8(CH₃C*H₃*)].

^{13}C-NMR (101 MHz, $CDCl_3$): δ [ppm] = 203.5 (s, O*C*CH₃), 154.7 (s, C3), 139.1 (d, C7), 126.9 (s, C2), 124.8 (d, C9), 57.1 (s, C1), 55.5 (q, OCH_3), 49.3 (s, C1'), 45.3 (t, C1'*C*H₂CHCH₂), 42.9 (s, C8), 36.6 (t, C1'CH₂CH*C*H₂), 35.2 (t, C9), 34.9 (q, OC*C*H₃), 31.1 (t, C4), 30.4 (d, C1'CH₂*C*HCH₂), 29.2 (d, C5), 28.2 [q, C8(*C*H₃CH₃)], 26.5 [q, C8(CH₃*C*H₃).

Assignment of the NMR signals was done as for substrate **4b** and with the aid of the ^{1}H NMR spectrum (see section *NMR Spectra of New Compounds*) of compound **4b**.

HRMS (ESI): $C_{24}H_{35}O_2S^+$ $[M+H]^+$ *m/z* calculated = 387.2353; found = 387.2351.

IR (ATR): $\tilde{\nu}$ [cm^{-1}] = 2893 (m, C−H), 1694 (s, C=O), 1627 (m), 1421 (m), 1271 (m), 1212 (m), 1150 (m, C−O), 1067 (m, C−S), 1036 (m), 1007 (m), 775 (m, C_{alkene}), 726 (m, C_{alkene}).

m.p.: 176 °C - 180 °C.

6. Synthesis of Bicyclic Nucleophile Addition Product with Benzoic Acid as Nucleophile

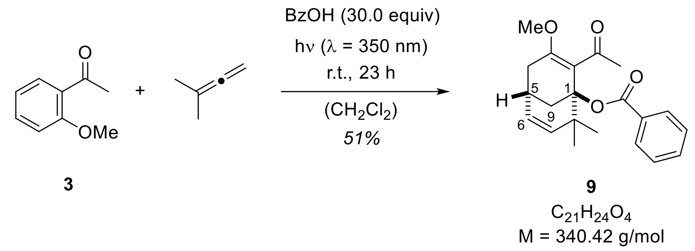

To a flame-dried Duran phototube, benzoic acid (487 mg, 4.00 mmol, 30.0 equiv) was added. Subsequently, the tube was evacuated and flushed with argon for three times. 2-Methoxyacetophenone (**3**, 18.4 µL, 20.0 mg, 133 µmol, 1.00 equiv), 1,1-dimethylallene (392 µL, 272 mg, 4.00 mmol, 30.0 equiv), and degassed dichloromethane (13.3 mL) were added. The solution was irradiated for 23 hours. Subsequently, the solvent and the residual allene were removed under reduced pressure. The crude mixture was dissolved in ethyl acetate (20 mL) and washed with 1 N NaOH solution (3 × 10 mL). The organic layer was washed with brine (1 × 10 mL), dried over Na_2SO_4, and filtered. The solvent was removed under reduced pressure. The crude product was purified by column chromatography (P/EtOAc = 4/1) to yield the product **9** (23.2 mg, 68.1 µmol, 51%) as a yellow powder.

TLC: R_f = 0.29 (P/EtOAc) = 3/1 [SM].

^{1}H-NMR (400 MHz, $CDCl_3$): *δ* [ppm] = 8.02-7.98 (m, 2H, C2'-H), 7.53-7.48 (m, 1H, C4'-H), 7.41 (dd, ^3J = 8.2 Hz, ^3J = 6.7 Hz, 2H, C3'-H), 5.61 (ddd, ^3J = 9.7 Hz, ^3J = 5.0 Hz, ^4J = 1.7 Hz, 1H, C6-H), 5.37 (d, ^3J = 9.7 Hz, 1H, C7-H), 3.70 (s, 3H, OCH_3), 2.83 (*virt.* dt, ^2J = 11.5 Hz, $^3J \approx {}^4J$ = 1.7 Hz, 1H, C4-*H*H), 2.77-2.67 (m, 2H, C5-H, C9-*H*H), 2.35-2.28 (m, 4H, $COCH_3$, C9-H*H*), 2.11 (ddd, ^2J = 11.5 Hz, ^3J = 3.7 Hz, ^4J = 1.1 Hz, 1H, C4-H*H*), 1.29 [s, 3H, C8(C*H₃*CH₃)], 1.07 [s, 3H, C8(CH₃C*H₃*)].

^{13}C-NMR (101 MHz, $CDCl_3$): *δ* [ppm] = 200.7 (s, O*C*CH*₃*), 166.3 (s, OCO), 162.2 (s, C3), 137.9 (d, C7), 132.8 (s, C1'), 132.3 (d, C4'), 129.6 (d, C2'), 128.3 (d, C3'), 126.6 (d, C6), 118.3 (s, C2), 84.3 (s, C1), 55.1 (q, OCH_3), 43.3 (s, C8), 33.4 (q, OC*C*H₃), 31.9 (t, C9), 30.2 (d, C5), 30.1 (t, C4), 26.3 [q, C8(*C*H₃CH₃)], 25.8 [q, C8(CH₃*C*H₃).

Assignment of the NMR signals was done as for substrate **4b** and with the aid of the ^{1}H NMR spectrum (see section *NMR Spectra of New Compounds*) of compound **4b**.

HRMS (ESI): $C_{21}H_{24}O_4Na^+$ [M+Na]$^+$ *m/z* calculated = 363.1567; found = 363.1566.

IR (ATR): ṽ [cm^{-1}] = 2924 (w, C–H), 1719 (m, C=O), 1666 (m, C=O), 1269 (m, C–O), 1111 (m), 1041 (m, C–O), 1027 (m, C–O), 770 (m, C_{alkene}), 703 (s, C–H_{Ar}).

m.p.: 165 °C - 166 °C.

7. Synthesis of Bicyclic Nucleophile Addition Product with Neopentylamine as Nucleophile

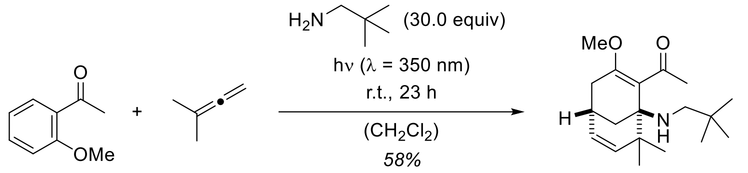

3

11
$C_{19}H_{31}NO_2$
M = 305.46 g/mol

To a flame-dried Duran phototube, 2-methoxyacetophenone (**3**, 18.4 µL, 20.0 mg, 133 µmol, 1.00 equiv), 1,1-dimethylallene (392 µL, 272 mg, 4.00 mmol, 30.0 equiv), and neopentylamine (471 µL, 348 mg, 4.00 mmol, 30.0 equiv) were added. Degassed dichloromethane (13.3 mL) was added, and the reaction solution was irradiated for 23 hours. The solvent, the residual allene, and the nucleophile were removed under reduced pressure, and the crude product was purified by column chromatography [CH_2Cl_2/MeOH + vol% (sat. NH_3-solution) = 97/3 + 0.1 → 96/4 + 0.175 → 95/5 + 0.25]. Product **11** (23.5 mg, 76.9 µmol, 58%) was obtained as a yellow oil.

TLC: R_f = 0.47 [CH_2Cl_2/MeOH + vol% (sat. NH_3-solution)] = 19/1 + 0.25 [SM].

^{1}H-NMR (400 MHz, $CDCl_3$): δ [ppm] = 5.51 (ddd, ^3J = 9.8 Hz, ^3J = 5.2 Hz, ^4J = 1.7 Hz, 1H, C6-H), 5.31 (d, ^3J = 9.8 Hz, 1H, C7-H), 3.64 (s, 3H, OCH_3), 2.61-2.55 (m, 1H, C5-H), 2.49 (dd, ^2J = 17.4 Hz, ^3J = 6.0 Hz, 1H, C4-*H*H), 2.30 (s, 3H, $COCH_3$), 2.26 (*virt.* dt, ^2J = 17.4 Hz, $^3J \approx {^4J}$ = 1.6 Hz, 1H, C4-H*H*), 2.11 (s, 2H, HNC*H_2*), 1.71 (ddd, ^2J = 12.6 Hz, ^3J = 4.3 Hz, ^4J = 1.6 Hz, 1H, C9-*H*H), 1.63 (*virt.* dt, ^2J = 12.6 Hz, $^3J \approx {^4J}$ = 1.7 Hz, 1H, C9-H*H*), 1.06 [s, 3H, C8(C*H_3*CH_3)], 0.87 [s, 9H, $C(CH_3)_3$], 0.84 [s, 3H, C8(CH_3C*H_3*)].

^{13}C-NMR (101 MHz, $CDCl_3$): δ [ppm] = 202.8 (s, CO), 164.1 (s, C3), 139.7 (d, C7), 125.7 (d, C6), 121.7 (s, C2), 61.2 (s, C1), 55.0 (q, OCH_3), 53.5 (t, $HNCH_2$), 42.8 (s, C8), 33.8 (q, OC*CH_3*), 32.6 (t, C4), 31.9 [s, *C*$(CH_3)_3$], 30.0 (d, C9), 28.8 (d, C5), 28.1 [q, C(*CH_3*)$_3$], 26.5 [q, C8(*CH_3*)$_2$].

Assignment of the NMR signals was done as for substrate **4b** and with the aid of the ^{1}H NMR spectrum (see section *NMR Spectra of New Compounds*) of compound **4b**.

HRMS (ESI): $C_{19}H_{32}NO_2^+$ $[M+H]^+$ *m/z* calculated = 306.2428; found = 306.2426.

IR (ATR): ṽ [cm^{-1}] = 2952 (m, C−H), 1650 (m, C=O), 1593 (m), 1366 (m), 1204 (m), 1150 (m), 1121 (m), 1064 (m), 732 (s, C_{alkene}).

8. Synthesis of Photocycloaddition Products from 2-Methoxybenzoic Acid Methyl Ester

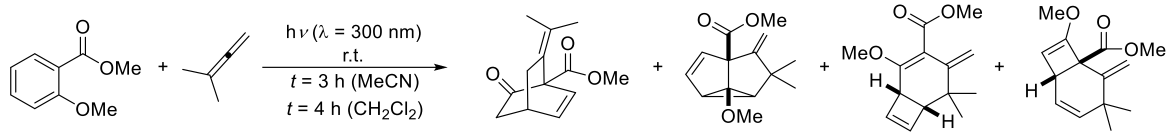

12 13 14 15 16

To a flame-dried quartz phototube, 2-methoxybenzoic acid methyl ester (**12**, 17.3 µL, 20.0 mg, 120 µmol, 1.00 equiv), 1,1-dimethylallene (354 µL, 245 mg, 3.61 mmol, 30.0 equiv), and the degassed solvent (12.0 mL) were added. The solution was irradiated at 300 nm at room temperature for the given reaction time. The solvent was removed under reduced pressure, and the crude product was purified by column chromatography (P/EtOAc = 12/1 → 6/1 → 4/1) yielding the respective photoproducts as a colorless oil.

8.1. Methyl (1*S*,4*S*)-6-oxo-7-(propan-2-ylidene)bicyclo[2.2.2]oct-2-ene-1-carboxylate (13)

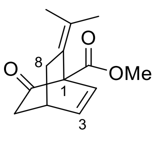

13
$C_{13}H_{16}O_3$
M = 220.27 g/mol

Yields: 4.71 mg, 21.4 µmol, 18%(in CH_2Cl_2), 6.91 mg, 31.3 µmol, 26% (in MeCN)

TLC: R_f = 0.46 (P/EtOAc) = 2/1 [SM].

^{1}H-NMR (400 MHz, $CDCl_3$): δ [ppm] = 6.68 (dd, ^3J = 8.1 Hz, ^4J = 2.2 Hz, 1H, C2-H*), 6.43 (dd, ^3J = 8.1 Hz, ^3J = 6.4 Hz, 1H, C3-H*), 3.85 (s, 3H, $OCOCH_3$), 3.04 (m, 1H, C4-H), 2.37-2.40 (m, 2H, $C7CH_2$), 2.30 (*virt.* ddt, ^2J = 18.0 Hz, ^3J = 3.6 Hz, $^4J \approx {}^4J$ = 1.9 Hz, 1H, C5-*H*H), 2.17 (dd, ^2J = 18.0 Hz, ^3J = 2.4 Hz, 1H, C5-H*H*), 1.67 [t, ^5J = 1.3 Hz, 3H, C7C($CH_3$$CH_3$)], 1.58 [t, ^5J = 1.8 Hz, 3H, C7C($CH_3$$CH_3$)].

*Signals are interchangable.

^{13}C-NMR (101 MHz, $CDCl_3$): δ [ppm] = 203.7 (s, C6), 170.8 (s, O*C*OCH_3), 135.5 (d, C3*), 129.7 (d, C2*), 128.2 [s, C7*C*(CH_3CH_3)**], 124.6 (s, C7**), 66.6 (s, C1), 52.3 (q, OCO*C*H_3), 40.7 (t, C5), 34.9 (t, C8), 31.7 (d, C4), 24.2 [q, C7C(*C*H_3CH_3)], 21.3 [q, C5(CH_3*C*H_3)].

*Signals are interchangable.

**Signals are interchangable.

While an alternative structure was possible with the observed HMBC signal pattern, the structure proposal for compound **13** seems more conclusive since the same, normally rare, ^5J COSY coupling between the vinylic methyl groups and the protons at carbon atom C8 could be observed, as for the compounds **2** resulting from the *para*-photocycloaddition reactions described in this report (see Figure S4). Additionally, this structure proposal is more likely from a mechanistical point of view since the formation of product **13** can be photochemically explained whereas we could not explain the formation of the other conceivable structure proposal.

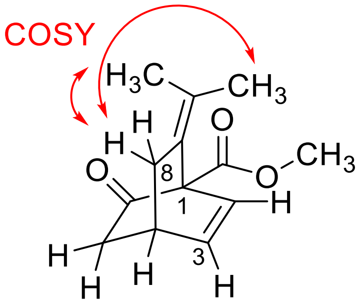

Figure S4: Characteristic ^5J COSY coupling between the vinylic methyl groups and the protons at carbon atom C8 of compound **13** as observed for structurally similar compounds **2**. This coupling implies that the structure of compound **13** might also originate from a *para*-photocycloaddition reaction pathway. COSY couplings are displayed with red arrows.

HRMS (ESI): $C_{13}H_{17}O_3^+$ $[M+H]^+$ *m/z* calculated = 221.1172; found = 221.1168.

IR (ATR): ṽ [cm^{-1}] = 2945 (w, C−H), 2922 (w, C−H), 1726 (s, C=O), 1436 (m), 1274 (m, C−O), 1244 (m, C−O), 1068 (m), 727 (m, C_{alkene}).

8.2. Methyl (2a^1*R*,4a*S*)-2a^1-methoxy-2,2-dimethyl-1-methylene-2,2a,2a^1,2b-tetrahydrocyclopropa[cd]pentalene-4a(1*H*)-carboxylate (14)

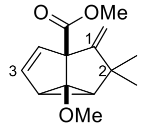

14

$C_{14}H_{18}O_3$

M = 234.30 g/mol

Yields: 4.72 mg, 20.2 µmol, 17% (in CH_2Cl_2), 3.83 mg, 16.3 µmol, 14% (in MeCN)

TLC: R_f = 0.47 (P/EtOAc) = 6/1 [SM].

^{1}H-NMR (400 MHz, $CDCl_3$): δ [ppm] = 6.32 (dd, ^3J = 2.7 Hz, ^4J = 0.7 Hz, 1H, C4-H), 6.30 (dd, ^3J = 2.7 Hz, ^3J = 1.4 Hz, 1H, C3-H), 5.10 (d, ^2J = 0.9 Hz, 1H, C1C-*H*H), 5.07 (d, ^2J = 0.9 Hz, 1H, C1C-H*H*), 3.70 (s, 3H, $OCOCH_3$), 3.46-3.45 (m, 1H, C2b-H), 3.39 (s, 3H, C2a^{1}OCH3), 2.46 (s, 1H, C2a-H), 1.29 [s, 3H, C2(C*H*$_3$CH$_3$)], 1.15 [s, 3H, C2(CH$_3$C*H*$_3$)].

^{13}C-NMR (101 MHz, $CDCl_3$): δ [ppm] = 171.9 (s, CO), 152.7 (s, C1), 140.5 (d, C4*), 139.5 (d, C3*), 108.4 (t, C1*C*H$_2$), 86.9 (s, C2a^1), 61.4 (s, C4a), 53.9 (q, C2a^{1}O*C*H$_3$), 51.9 (q, OCO*C*H$_3$), 49.7 (d, C2a), 48.7 (d, C2b), 41.1 (s, C2), 29.7 [q, C2(*C*H$_3$CH$_3$)], 21.8 [q, C2(CH$_3$*C*H$_3$)].

From the HMBC and COSY spectra of compound **14** (see section *NMR Spectra of New Compounds*), two molecule fragments can be determined (see Figure S5). The left one originates from the COSY coupling of the proton at C2b and the olefinic proton at carbon atom C3 and the one in the middle from the HMBC correlations of the terminal olefinic protons to the surrounding carbon atoms. If the HMBC correlations of the proton at C2a are considered, the only possible connection of these two fragments is the one described for compound **14**. The assignment remains tentative as the expected COSY coupling of the proton at carbon atom C2a to the proton at carbon atom C2b was not observed.

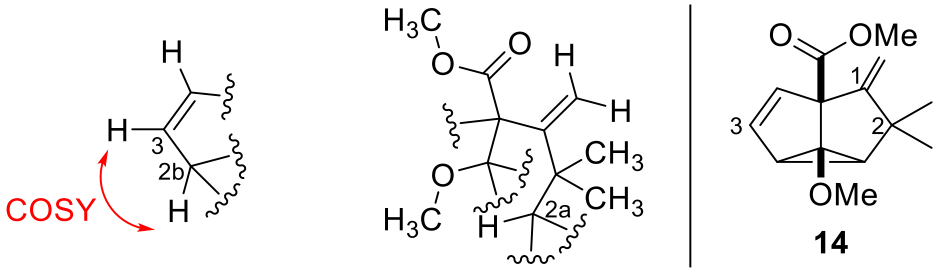

Figure S5: Two fragments resulting from the COSY and HMBC spectra analysis of compound **14** on the left-hand side. Structural proposal of compound **14** after analysis of the HMBC correlations of the proton at C2a on the right. COSY coupling marked with red arrows.

HRMS (ESI): $C_{14}H_{19}O_3^+$ $[M+H]^+$ *m/z* calculated = 235.1329; found = 235.1329.

IR (ATR): ṽ [cm^{-1}] = 2952 (w, C–H), 2928 (w, C–H), 1726 (s, C=O), 1434 (w), 1283 (m, C–O), 1244 (m, C–O), 1198 (m, C–O), 1061 (m), 887 (m), 749 (m, C_{alkene}).

8.3. Methyl (1*R*,6*S*)-2-methoxy-5,5-dimethyl-4-methylenebicyclo[4.2.0]octa-2,7-diene-3-carboxylate (15)

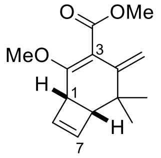

15
$C_{14}H_{18}O_3$
M = 234.30 g/mol

Yields: 3.53 mg, 15.1 µmol, 13% (in CH_2Cl_2), 3.10 mg, 13.2 µmol, 11% (in MeCN)

TLC: R_f = 0.49 ($PhCH_3$/EtOAc) = 8/1 [SM].

^{1}H-NMR (500 MHz, $CDCl_3$): δ [ppm] = 6.22 (dd, ^3J = 2.9 Hz, ^3J = 1.1 Hz, 1H, C7-H), 6.14 (*virt.* dt, ^3J = 2.9 Hz, $^3J \approx {}^4J$ = 1.1 Hz, 1H, C8-H), 4.88 (s, 1H, C4C-*H*H), 4.79 (s, 1H, C4C-H*H*), 3.77 (s, 3H, $OCOCH_3$), 3.73 (s, 3H, $C2OCH_3$), 3.59 (d, ^3J = 4.1 Hz, 1H, C1-H), 2.96 (*virt.* dt, ^3J = 4.1 Hz, $^3J \approx {}^4J$ = 1.1 Hz, 1H, C6-H), 1.21 [s, 3H, C5(CH_3CH_3)], 1.04 [s, 3H, C5(CH_3CH_3)].

^{13}C-NMR (101 MHz, $CDCl_3$): δ [ppm] = 169.3 (s, CO), 157.2 (s, C2), 146.6 (s, C4), 140.0 (d, C7), 136.7 (d, C8), 111.1 (s, C3), 106.9 (t, $C2CH_2$), 56.1 (q, $OCOCH_3$), 53.8 (d, C1), 52.0 (d, C6), 40.9 (q, $C2OCH_3$), 38.1 (s, C5), 28.1 [q, C5(CH_3CH_3)], 23.4 [q, C5(CH_3CH_3)].

The COSY couplings of the olefinic protons at C7 and C8 to the protons at carbon atoms C1 and C6, respectively (see section *NMR Spectra of New Compounds*), as well as the coupling of the protons at these tertiary carbon centers with each other propose a cyclobutene ring as part of the structure of compound **15** (see Figure S6). Under consideration of the HMBC correlations of the dimethyl group and the terminal olefin protons and the characteristic olefinic chemical shift of C3, the scaffold shown on the left hand-side was determined to be part of the proposed structure of compound **15**. Further analysis of the HMBC data completed the deduction of the bicyclic carbon skeleton of compound **15**.

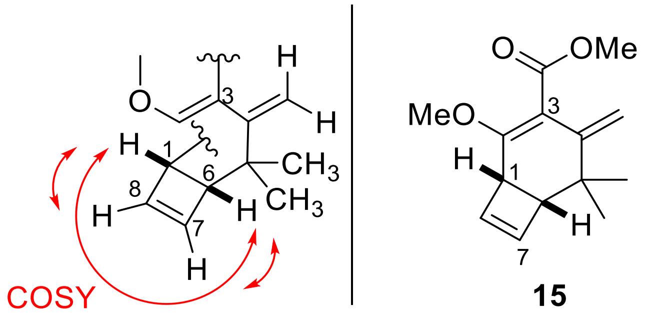

Figure S6: Molecule fragment deduced from the COSY couplings of compound **15** and the HMBC correlations of the corresponding dimethyl group and terminal olefin protons (left side). Deduced structure of compound **15** after further HMBC analysis (right side).

HRMS (ESI): $C_{14}H_{19}O_3^+$ $[M+H]^+$ *m/z* calculated = 235.1329; found = 235.1329.

IR (ATR): ṽ [cm^{-1}] = 2955 (w, C–H), 2925 (w, C–H), 1777 (m), 1727 (s, C=O), 1665 (m, C=C), 1435 (m), 1242 (s, C–O), 1055 (m), 1061 (m), 757 (m, C_{alkene}).

8.4. Methyl (1*S*,6*S*)-8-methoxy-3,3-dimethyl-2-methylenebicyclo[4.2.0]octa-4,7-diene-1-carboxylate (16)

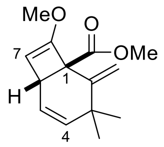

16

$C_{14}H_{18}O_3$

M = 234.30 g/mol

Yields: 5.80 mg, 24.8 µmol, 21% (in CH_2Cl_2), 3.26 mg, 13.9 µmol, 12% (in MeCN)

TLC: R_f = 0.53 (P/EtOAc) = 6/1 [SM].

^{1}H-NMR (400 MHz, $CDCl_3$): δ [ppm] = 5.68 (dd, ^3J = 10.0 Hz, ^3J = 3.0 Hz, 1H, C5-H), 5.58 (dd, ^3J = 10.0 Hz, ^4J = 1.5 Hz, 1H, C4-H), 5.35 (s, 1H, C2C-*H*H), 5.14 (s, 1H, C2C-H*H*), 4.77 (d, ^3J = 1.5 Hz, 1H, C7-H), 3.74 (s, 3H, $OCOCH_3$), 3.63 (s, 3H, $C8OCH_3$), 3.46 (*virt.* dt, ^3J = 3.0 Hz, $^3J \approx {}^4J$ = 1.5 Hz, 1H, C6-H), 1.23 [s, 3H, C3($CH_3$$CH_3$)], 1.12 [s, 3H, C3($CH_3$$CH_3$)].

^{13}C-NMR (101 MHz, $CDCl_3$): δ [ppm] = 173.0 (s, CO), 155.5 (s, C8), 149.6 (s, C2), 137.5 (d, C4), 126.8 (d, C5), 111.0 (t, C2CH_2), 99.4 (d, C7), 59.8 (s, C1), 56.0 (q, C8OCH_3), 52.6 (q, OCOCH_3), 39.4 (d, C6), 37.3 (s, C3), 30.3 [q, C3($CH_3$$CH_3$)], 28.5 [q, C3($CH_3$$CH_3$)].

Major parts of the structure of compound **16** can be derived from the COSY spectrum and the HMBC spectrum (see Figure S7 and section *NMR Spectra of New Compounds*). Only the bonds between carbon atoms C6 and C1 and C1 and C8 were yet to explain. The coupling of the proton at C6 to both, the olefinic protons at carbon atom C5 and C7 justify the proposal of the carbon skeleton from C4 to C8. Additional analysis of the correlations of the dimethyl group and the terminal olefin protons in the HMBC spectrum additionally deliver the framework from carbon atom C1 to C3. With this framework in hand, the only possible completion of the structure can be done in such a way that compound **16** is formed.

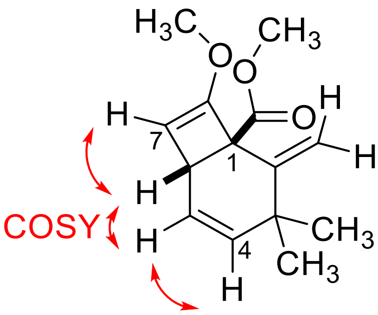

Figure S7: Analysis of the COSY spectrum and the HMBC correlations of the dimethyl group and the terminal olefin protons of compound **16** deliver the whole carbon skeleton of compound **16** except for the connection between carbon atom C6 and C1 and C1 and C8, respectively. However, there is only one possible way of completing this carbon framework leading to structure proposal **16**.

HRMS (ESI): $C_{14}H_{19}O_3^+$ $[M+H]^+$ *m/z* calculated = 235.1329; found = 235.1329.

IR (ATR): ṽ [cm^{-1}] = 2926 (w, C–H), 1727 (s, C=O), 1640 (m, C=C), 1434 (w), 1240 (s, C–O), 1045 (m), 753 (m, C_{alkene}).

9. Crystallographic Data

SC-XRD structure report for compound 2525384.

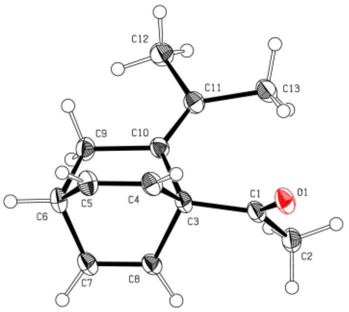

Figure S8: ORTEP representation of the solid-state structure of compound 2525384 (C = black, O = red) shown with 50 % probability displacement ellipsoids.

A colourless, block-shaped crystal of $C_{13}H_{16}O$ coated with perfluorinated ether and fixed on top of a Kapton micro sampler was used for X-ray crystallographic analysis. The crystals were prepared as described in the section *Materials and Methods.* The X-ray intensity data were collected at 100(2) K on a Bruker D8 VENTURE Duo three-angle diffractometer with an IMS microsource with MoK_{α} radiation (λ=0.71073 Å) using APEX4.[11] The diffractometer was equipped with a Helios optic monochromator, a Bruker PHOTON II detector, and an Oxford Cryostreamlow temperature device. A matrix scan was used to determine the initial lattice parameters. All data were integrated with the Bruker SAINT V8.41 software package using a narrow-frame algorithm and the reflections were corrected for Lorentz and polarisation effects, scan speed, and background.[12] The integration of the data using a monoclinic unit cell yielded a total of 22843 reflections within a 2θ range [°] of 4.40 to 50.83 (0.83 Å), of which 1923 were independent. Data were corrected for absorption effects including odd and even ordered spherical harmonics by the multi-scan method (SADABS 2016/2).[13] Space group assignment was based upon systematic absences, E statistics, and successful refinement of the structure.

The structure was solved by dual space methods using SHELXT and refined by full-matrix least-squares methods against F^2 by minimizing $\Sigma w(F_o^2 - F_c^2)^2$ using SHELXL in conjunction with SHELXLE.[14-16] All non-hydrogen atoms were refined with anisotropic displacement parameters. Hydrogen atoms were refined isotropically on calculated positions using a riding model with their U_{iso} values constrained to 1.5 times the U_{eq} of their pivot atoms for terminal sp^3 carbon atoms and a C–H distance of 0.98 Å. Non-methyl hydrogen atoms were refined using a riding model with methylene, aromatic, and other C–H distances of 0.99 Å, 0.95 Å, and 1.00 Å, respectively, and U_{iso} values constrained to 1.2 times the U_{eq} of their pivot atoms.

Neutral atom scattering factors for all atoms and anomalous dispersion corrections for the non-hydrogen atoms were taken from International Tables for Crystallography.[17] Supplementary crystallographic data reported in this paper have been deposited with the Cambridge Crystallographic Data Centre (CCDC) and can be obtained free of charge from The Cambridge Crystallographic Data Centre via www.ccdc.cam.ac.uk/structures.[18] This report and the CIF file were generated using FinalCif.[19]

Table 1: Crystal data and structure refinement for compound 2525384.

CCDC number	2525384
Empirical formula	$C_{13}H_{16}O$
Formula weight	188.26
Temperature [K]	100(2)
Crystal system	monoclinic
Space group (number)	$P2_1/c$ (14)
a [Å]	8.1274(14)
b [Å]	18.506(4)
c [Å]	7.7516(14)
α [°]	90
β [°]	116.043(6)
γ [°]	90
Volume [Å^3]	1047.5(3)
Z	4
ρ_{calc} [gcm^{-3}]	1.194
μ [mm^{-1}]	0.073
F(000)	408
Crystal size [mm^3]	0.178×0.205×0.230
Crystal colour	colourless
Crystal shape	block
Radiation	MoK_α (λ=0.71073 Å)
2θ range [°]	4.40 to 50.83 (0.83 Å)
Index ranges	$-9 \leq h \leq 9$<br>$-22 \leq k \leq 22$<br>$-9 \leq l \leq 9$
Reflections collected	22843
Independent reflections	1923<br>R_{int} = 0.0664<br>R_{sigma} = 0.0264
Completeness to θ = 25.242°	100.0
Data / Restraints / Parameters	1923 / 0 / 130
Goodness-of-fit on F^2	1.099
Final R indexes [$I \geq 2\sigma(I)$]	R_1 = 0.0477<br>wR_2 = 0.1066
Final R indexes [all data]	R_1 = 0.0674<br>wR_2 = 0.1237
Largest peak/hole [eÅ^{-3}]	0.20/−0.27

SC-XRD structure report for compound 2525385.

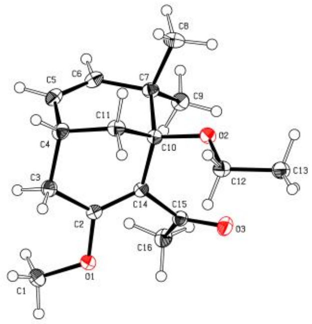

Figure S9: ORTEP representation of the solid-state structure of compound 2525385 (C = black and O = red) shown with 50 % probability displacement ellipsoids.

A colourless, plate-shaped crystal of $C_{16}H_{24}O_3$ coated with perfluorinated ether and fixed on top of a Kapton micro sampler was used for X-ray crystallographic analysis. The crystals were prepared as described in the section *Materials and Methods.* The X-ray intensity data were collected at 100(2) K on a Bruker D8 VENTURE Duo three-angle diffractometer with an IMS microsource with MoK_α radiation (λ=0.71073 Å) using APEX4.[11] The diffractometer was equipped with a Helios optic monochromator, a Bruker PHOTON II detector, and an Oxford Cryostreamlow temperature device.

A matrix scan was used to determine the initial lattice parameters. All data were integrated with the Bruker SAINT V8.41 software package using a narrow-frame algorithm and the reflections were corrected for Lorentz and polarisation effects, scan speed, and background.[12] The integration of the data using a triclinic unit cell yielded a total of 85121 reflections within a 2θ range [°] of 5.42 to 56.65 (0.75 Å), of which 7193 were independent. Data were corrected for absorption effects including odd and even ordered spherical harmonics by the multi-scan method (SADABS 2016/2).[13] Space group assignment was based upon systematic absences, E statistics, and successful refinement of the structure.

The structure was solved by dual space methods using SHELXT and refined by full-matrix least-squares methods against F^2 by minimizing $\Sigma w(F_o^2 - F_c^2)^2$ using SHELXL in conjunction with SHELXLE.[14-16] All non-hydrogen atoms were refined with anisotropic displacement parameters. Hydrogen atoms were refined isotropically on calculated positions using a riding model with their U_{iso} values constrained to 1.5 times the U_{eq} of their pivot atoms for terminal sp^3 carbon atoms and a C–H distance of 0.98 Å. Non-methyl hydrogen atoms were refined using a riding model with methylene, aromatic, and other C–H distances of 0.99 Å, 0.95 Å, and 1.00 Å, respectively, and U_{iso} values constrained to 1.2 times the U_{eq} of their pivot atoms.

Neutral atom scattering factors for all atoms and anomalous dispersion corrections for the non-hydrogen atoms were taken from International Tables for Crystallography.[17] Supplementary crystallographic data reported in this paper have been deposited with the Cambridge Crystallographic Data Centre (CCDC) and can be obtained free of charge from The Cambridge Crystallographic Data Centre via www.ccdc.cam.ac.uk/structures.[18] This report and the CIF file were generated using FinalCif.[19]

Table 2: Crystal data and structure refinement for compound 2525385.

CCDC number	2525385
Empirical formula	$C_{16}H_{24}O_3$
Formula weight	264.35
Temperature [K]	100(2)
Crystal system	triclinic
Space group (number)	$P\overline{1}$ (2)
a [Å]	7.7280(6)
b [Å]	14.6984(11)
c [Å]	14.9349(11)
α [°]	114.420(3)
β [°]	102.964(3)
γ [°]	98.954(3)
Volume [Å^3]	1445.12(19)
Z	4
ρ_{calc} [gcm^{-3}]	1.215
μ [mm^{-1}]	0.082
F(000)	576
Crystal size [mm^3]	0.158×0.356×0.569
Crystal colour	colourless
Crystal shape	plate
Radiation	MoK_α (λ=0.71073 Å)
2θ range [°]	5.42 to 56.65 (0.75 Å)
Index ranges	−10 ≤ h ≤ 10<br>−19 ≤ k ≤ 19<br>−19 ≤ l ≤ 19
Reflections collected	85121
Independent reflections	7193<br>R_{int} = 0.0382<br>R_{sigma} = 0.0158
Completeness to θ = 25.242°	99.8
Data / Restraints / Parameters	7193 / 45 / 363
Goodness-of-fit on F^2	1.091
Final R indexes [I≥2σ(I)]	R_1 = 0.0459<br>wR_2 = 0.1029
Final R indexes [all data]	R_1 = 0.0592<br>wR_2 = 0.1166
Largest peak/hole [eÅ^{-3}]	0.32/−0.25

10. NMR Spectra of New Compounds

10.1. Products Resulting from Irradiation of *para*-Substituted Acetophenone Derivatives

*10.1.1. 1-[7-(Propan-2-ylidene)bicyclo[2.2.2]octa-2,5-dien-1-yl]ethan-1-one (**2a**)*

^{1}H NMR (500 MHz, $CDCl_3$):

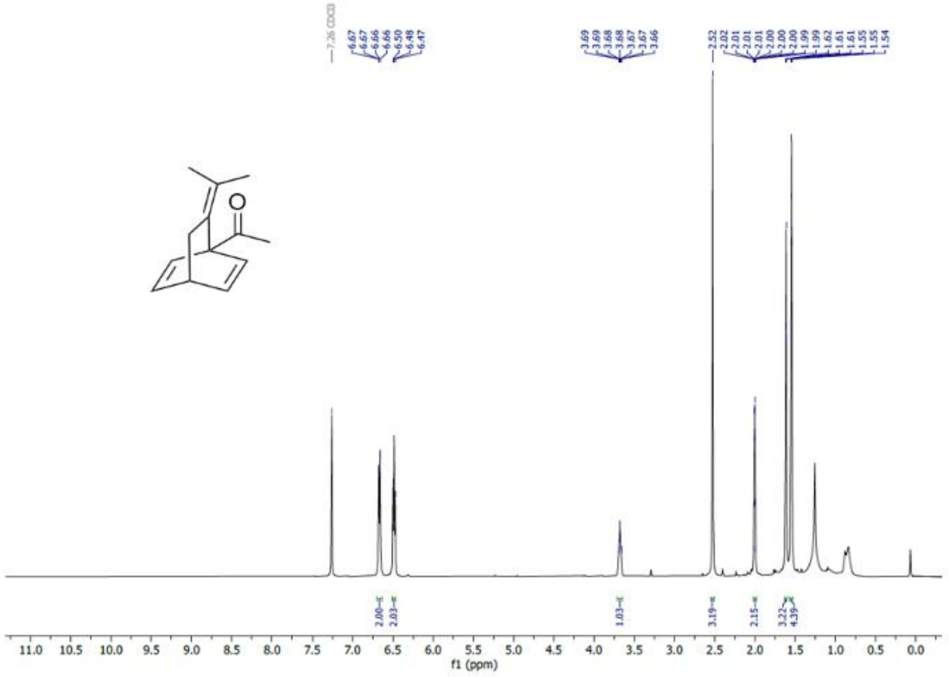

^{13}C NMR (101 MHz, $CDCl_3$):

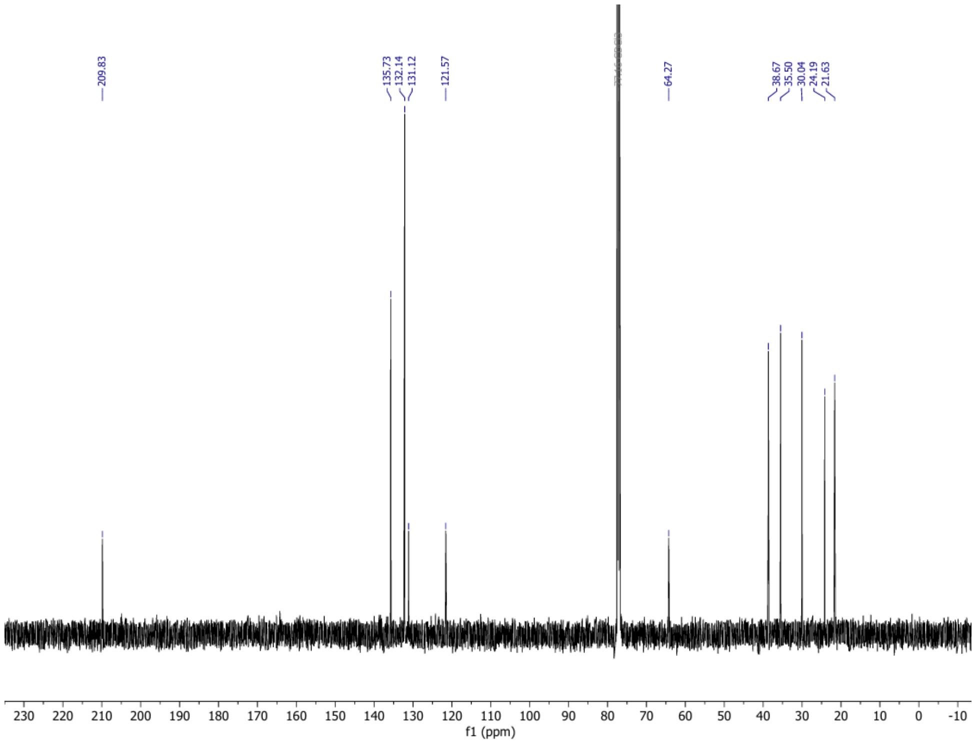

COSY spectrum:

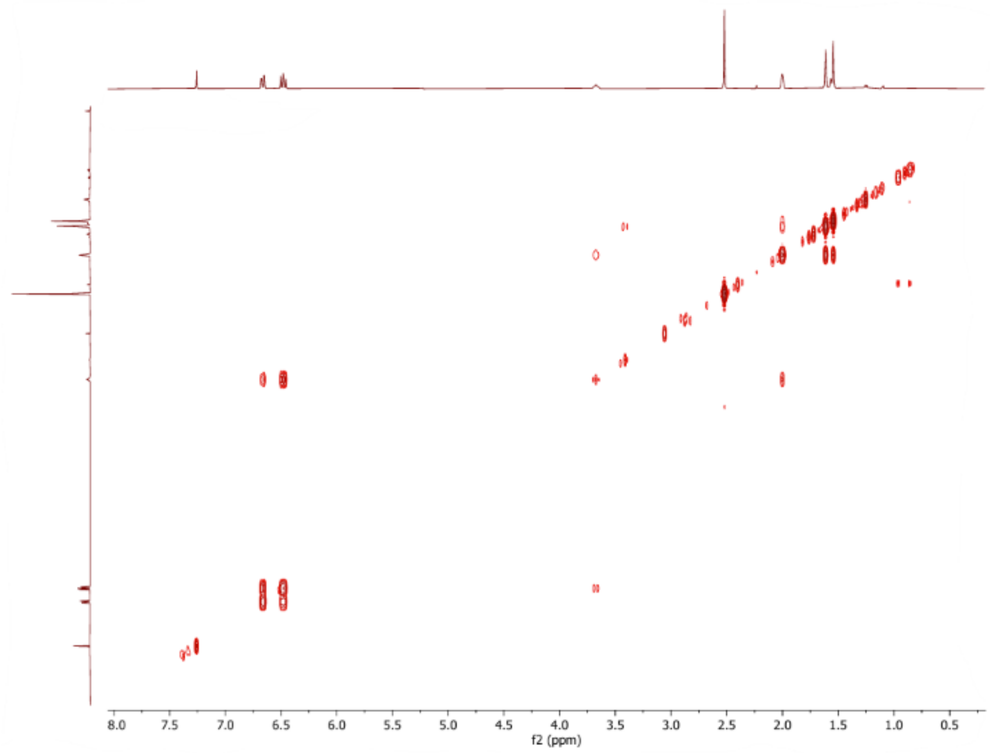

HSQC spectrum:

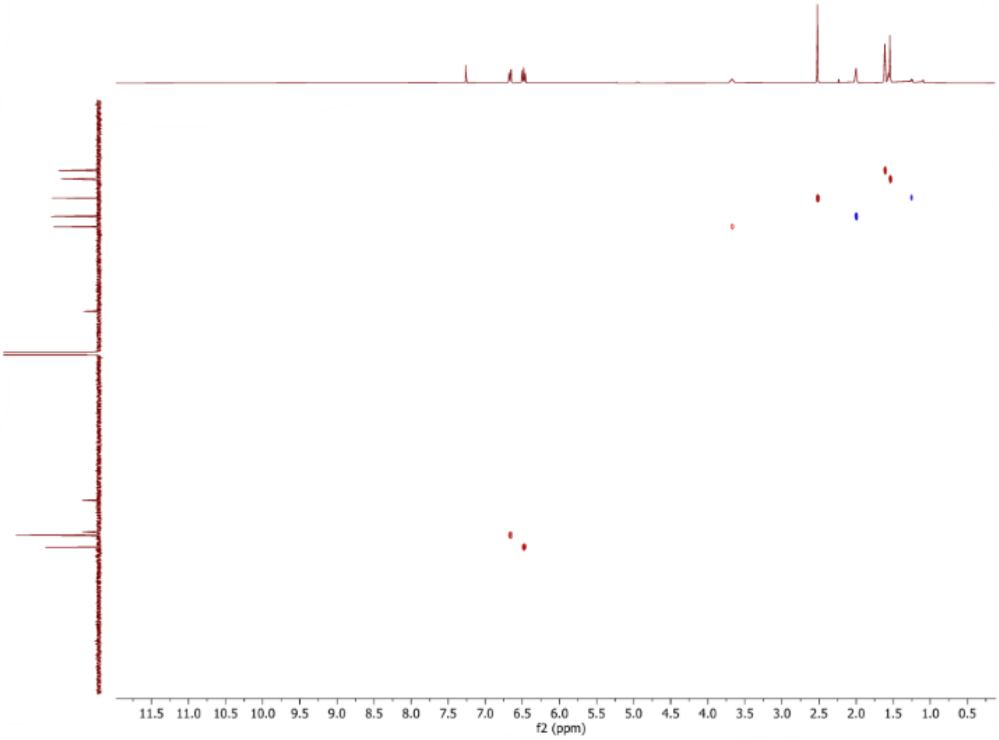

HMBC spectrum:

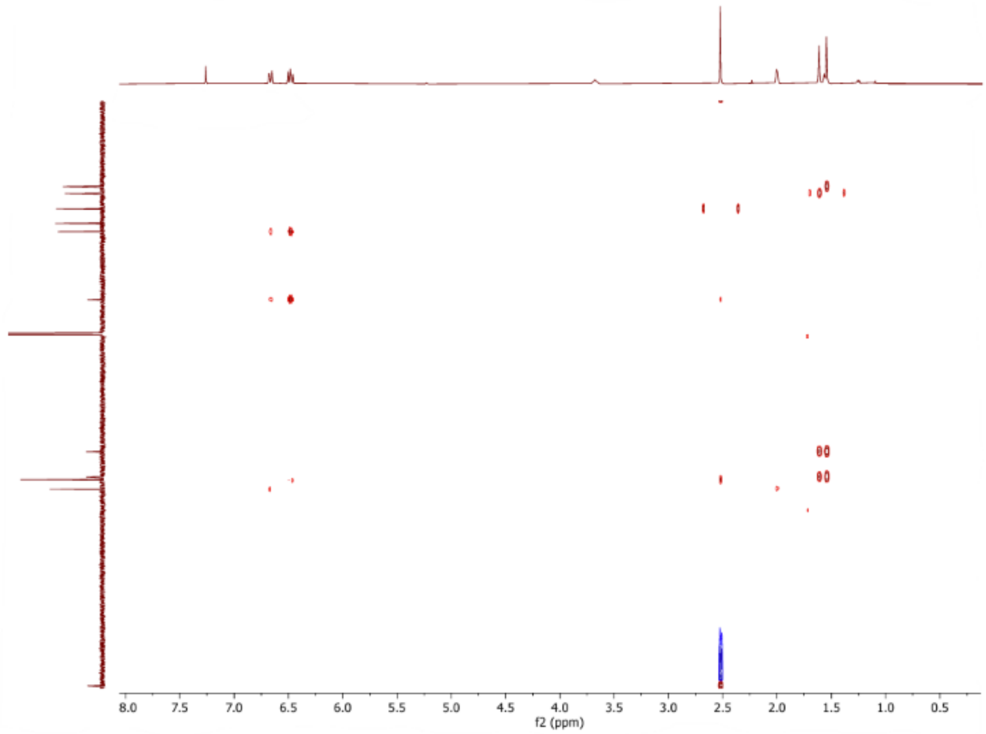

*10.1.2. 1-[4-Methyl-7-(propan-2-ylidene)bicyclo[2.2.2]octa-2,5-dien-1-yl]ethan-1-one (**2b**)*

^1H NMR (500 MHz, $CDCl_3$):

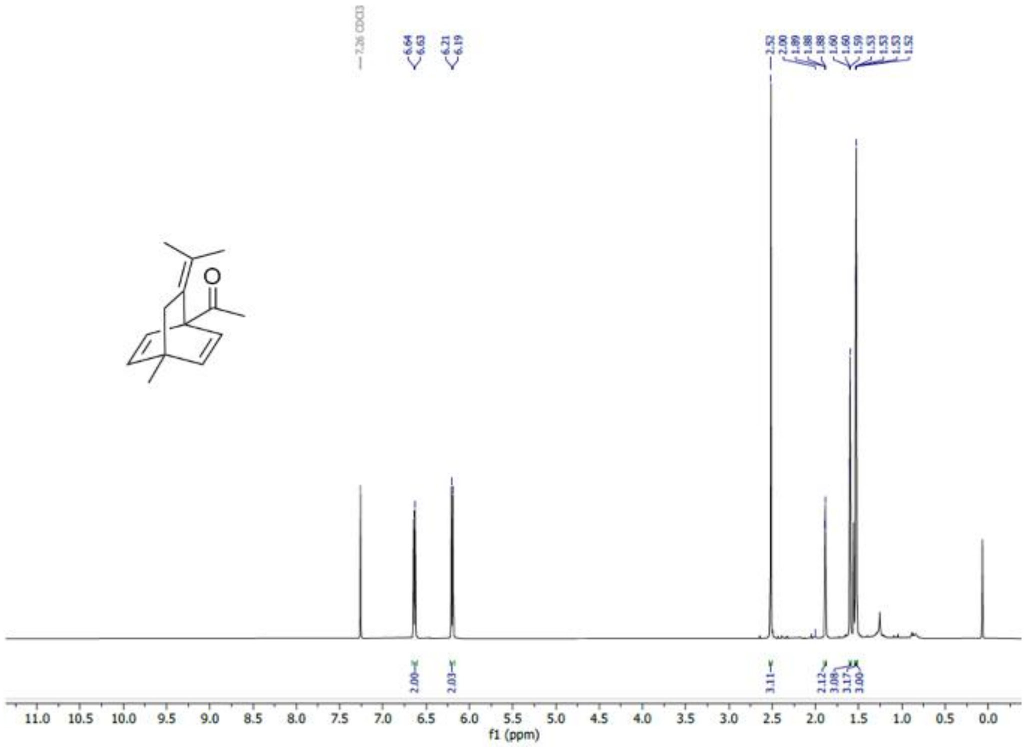

^{13}C NMR (101 MHz, $CDCl_3$):

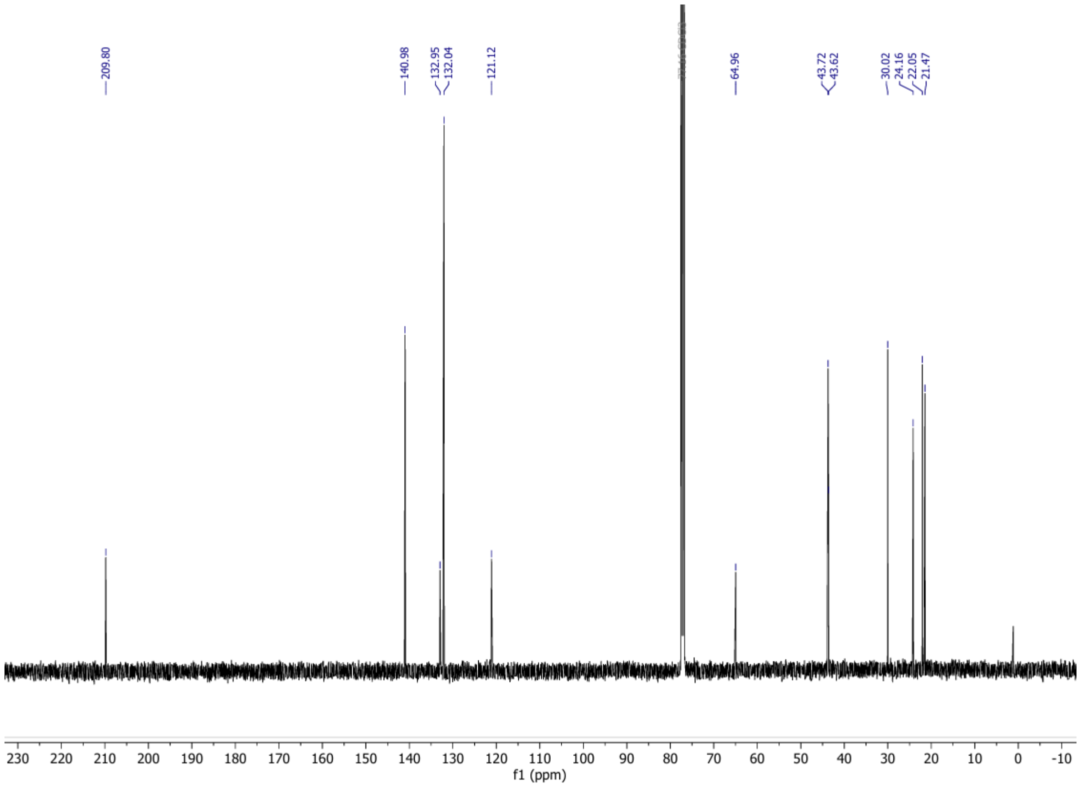

*10.1.3. 1-[4-(tert-Butyl)-7-(propan-2-ylidene)bicyclo[2.2.2]octa-2,5-dien-1-yl]ethan-1-one (**2c**)*

^{1}H NMR (500 MHz, $CDCl_3$):

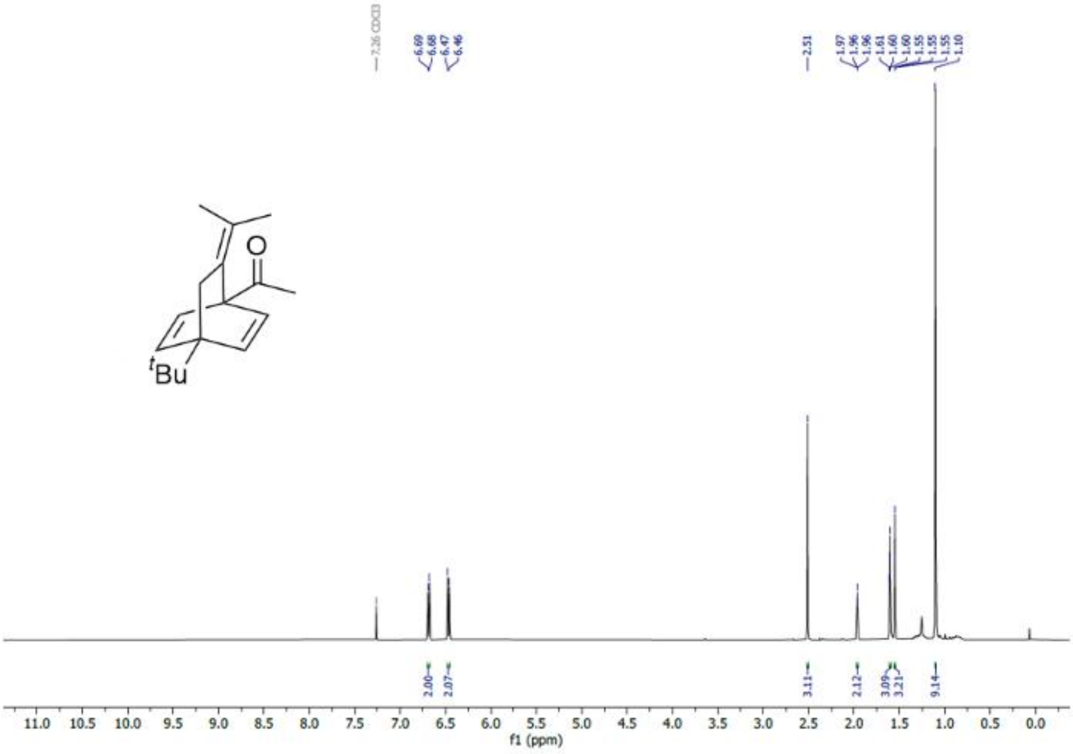

^{13}C NMR (101 MHz, $CDCl_3$):

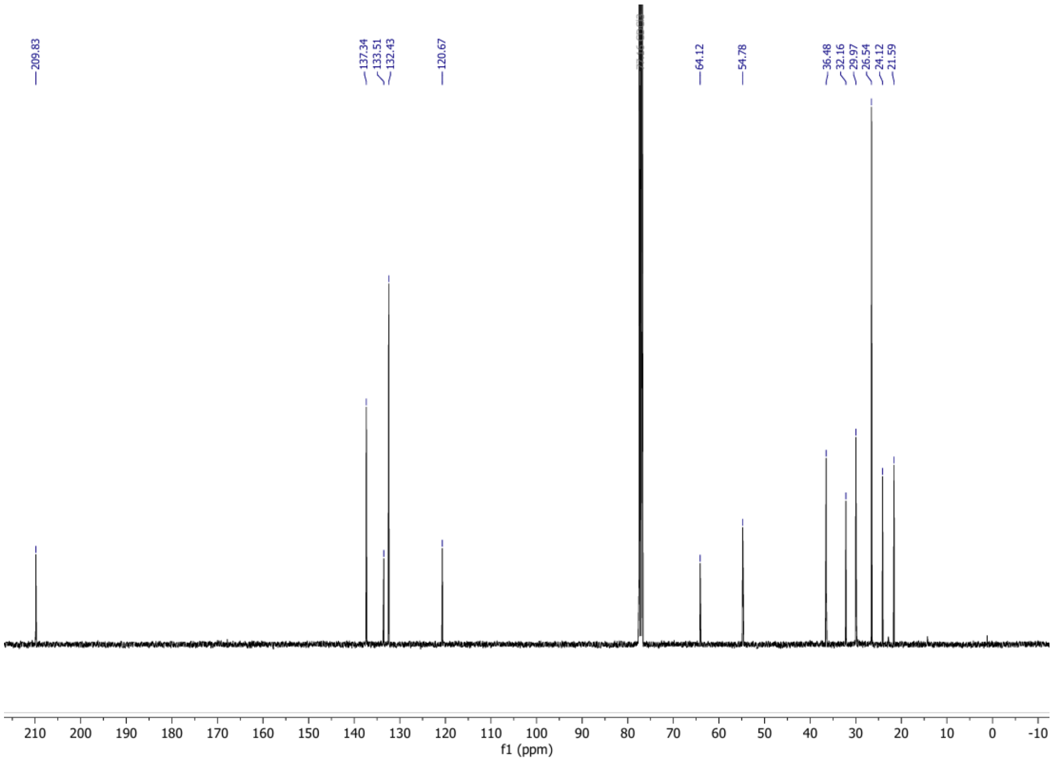

*10.1.4. 1-[4-Methoxy-7-(propan-2-ylidene)bicyclo[2.2.2]octa-2,5-dien-1-yl]ethan-1-one (**2d**)*

^{1}H NMR (400 MHz, $CDCl_3$):

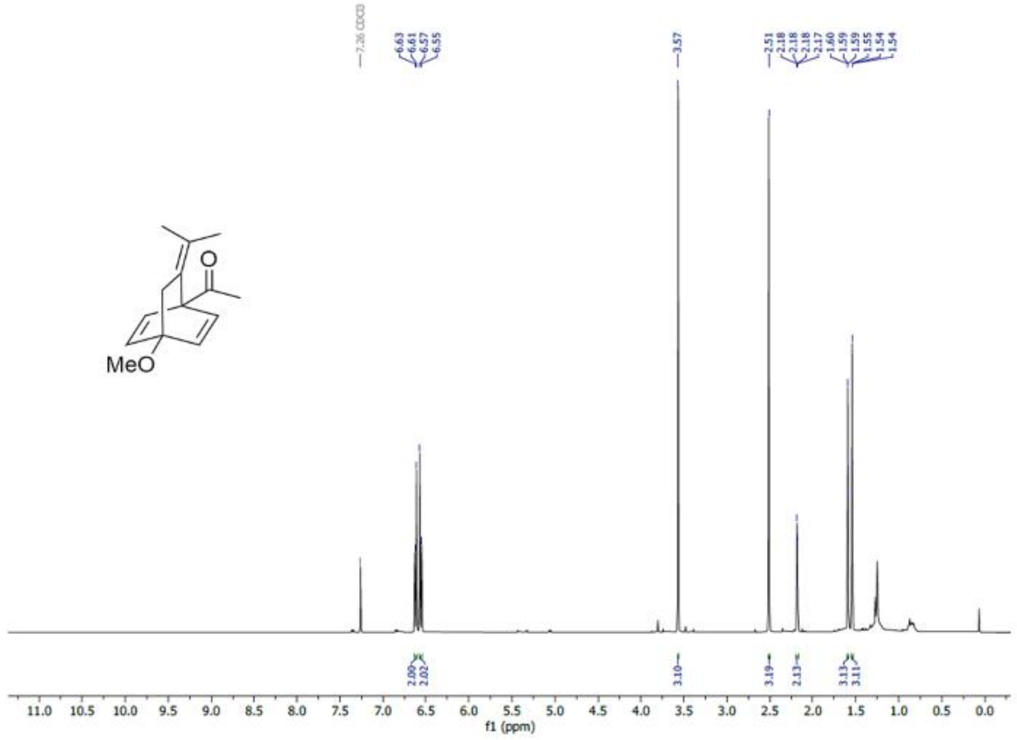

^{13}C NMR (101 MHz, $CDCl_3$):

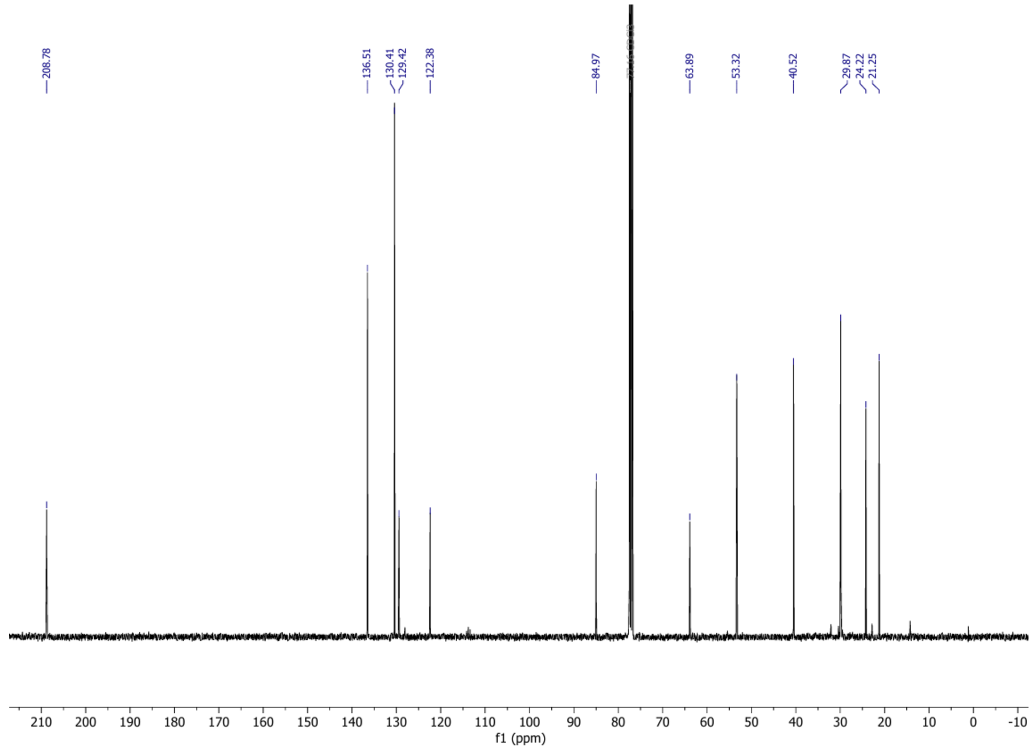

10.2. Nucleophile Addition Products

*10.2.1. 1-[(1S,5S)-1,3-Dimethoxy-8,8-dimethylbicyclo[3.3.1]nona-2,6-dien-2-yl]ethan-1-one (**4a**)*

^{1}H NMR (400 MHz, $CDCl_3$):

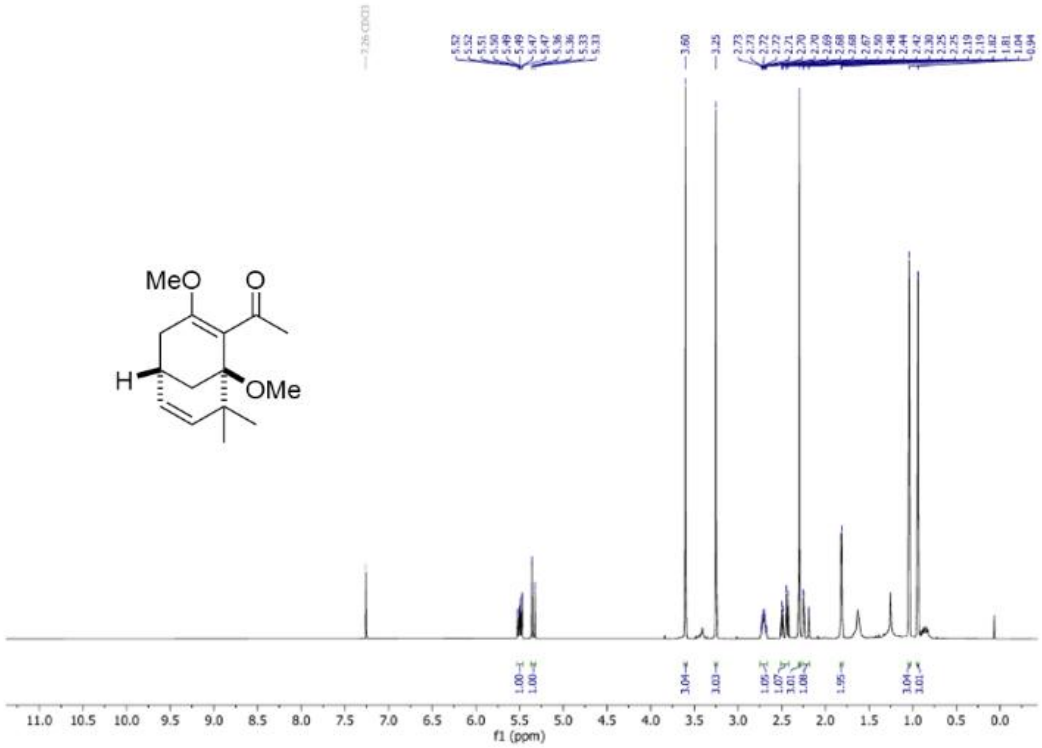

^{13}C NMR (101 MHz, $CDCl_3$):

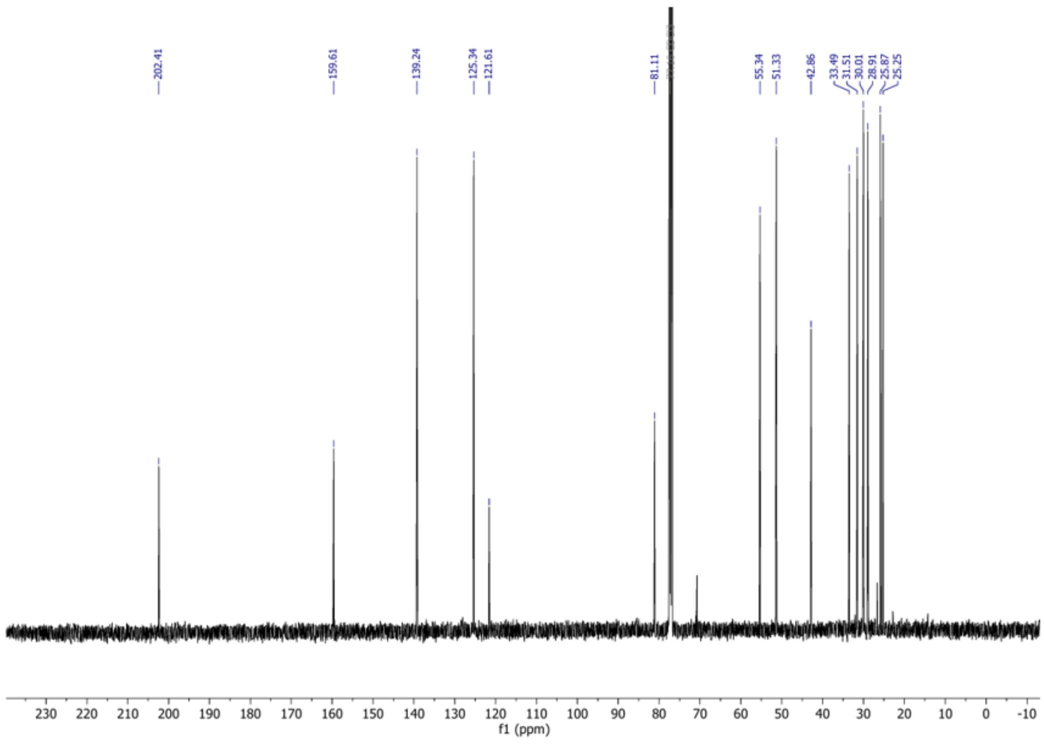

*10.2.2. 1-[(1S,5S)-1-Ethoxy-3-methoxy-8,8-dimethylbicyclo[3.3.1]nona-2,6-dien-2-yl]ethan-1-one (**4b**)*

^{1}H NMR (400 MHz, $CDCl_3$):

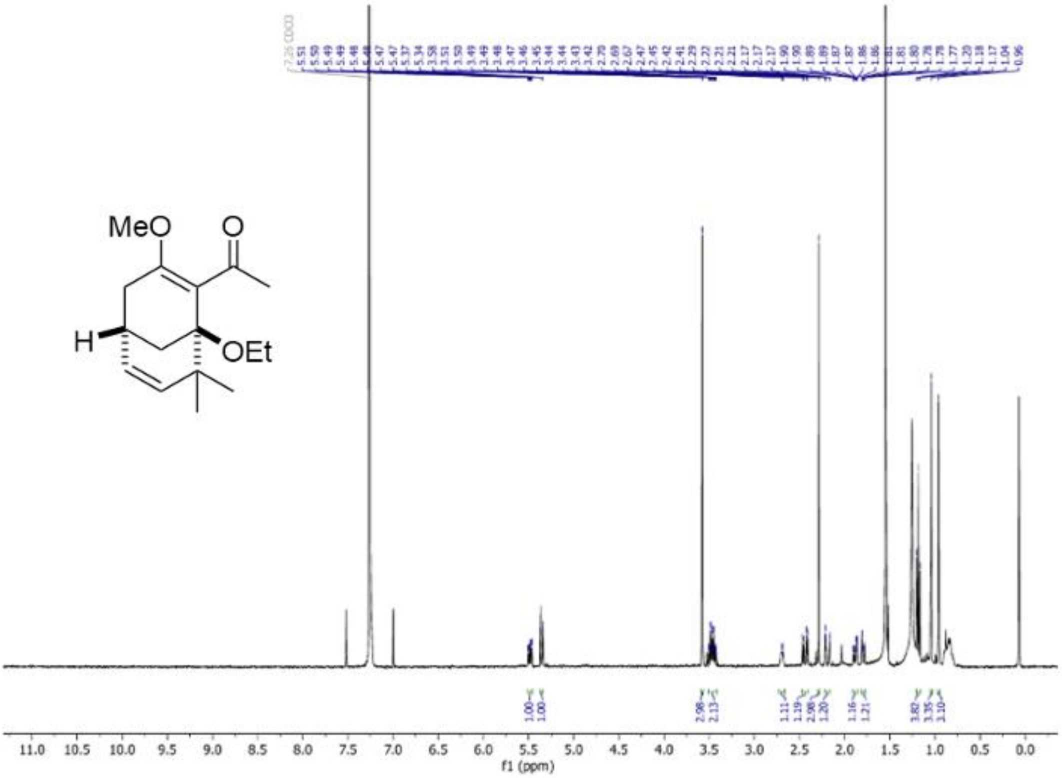

^{13}C NMR (126 MHz, $CDCl_3$):

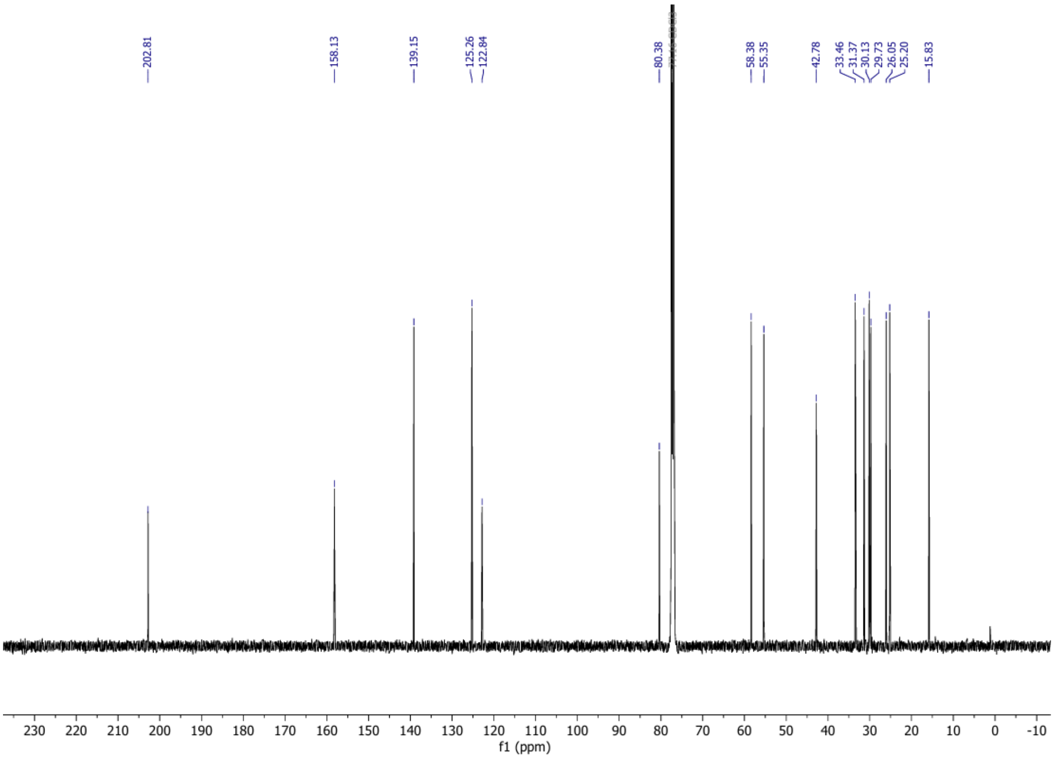

COSY spectrum:

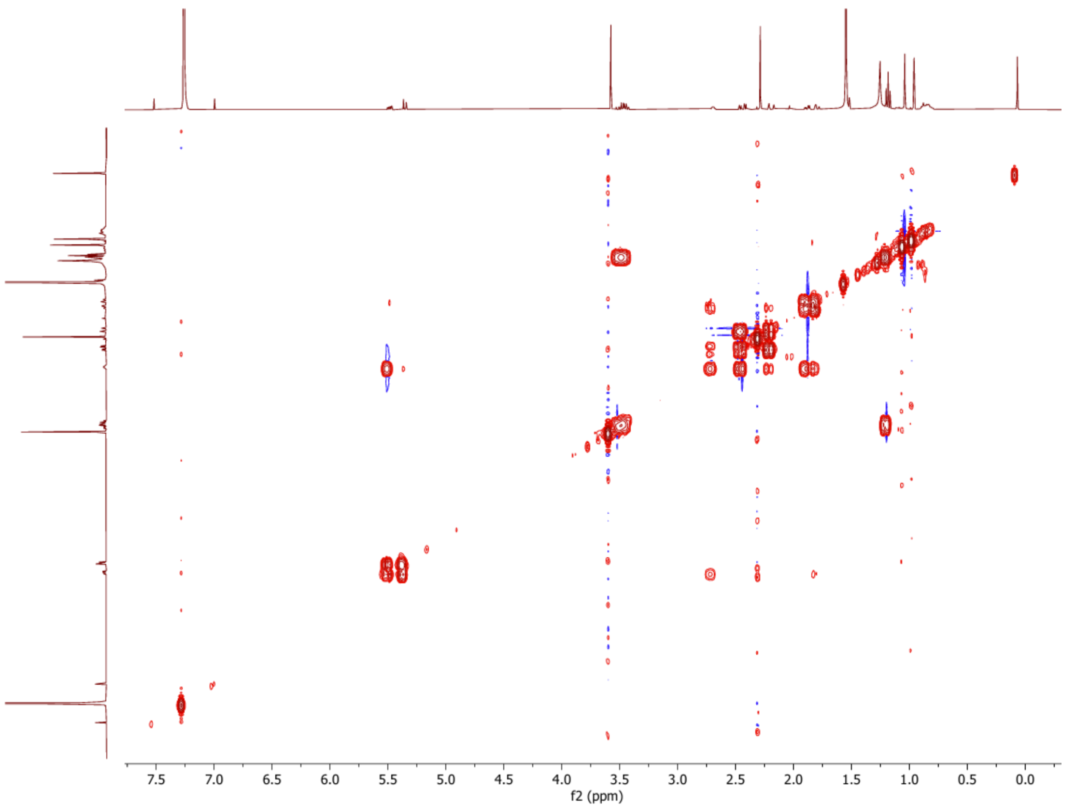

HSQC spectrum:

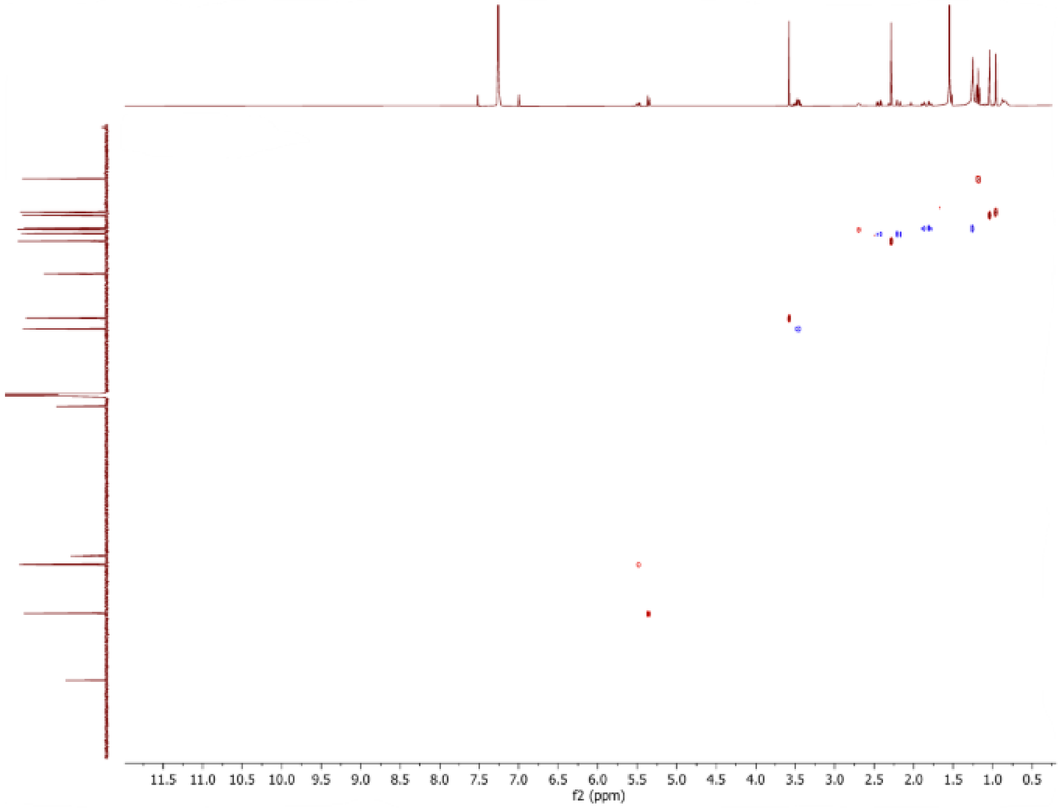

HMBC spectrum:

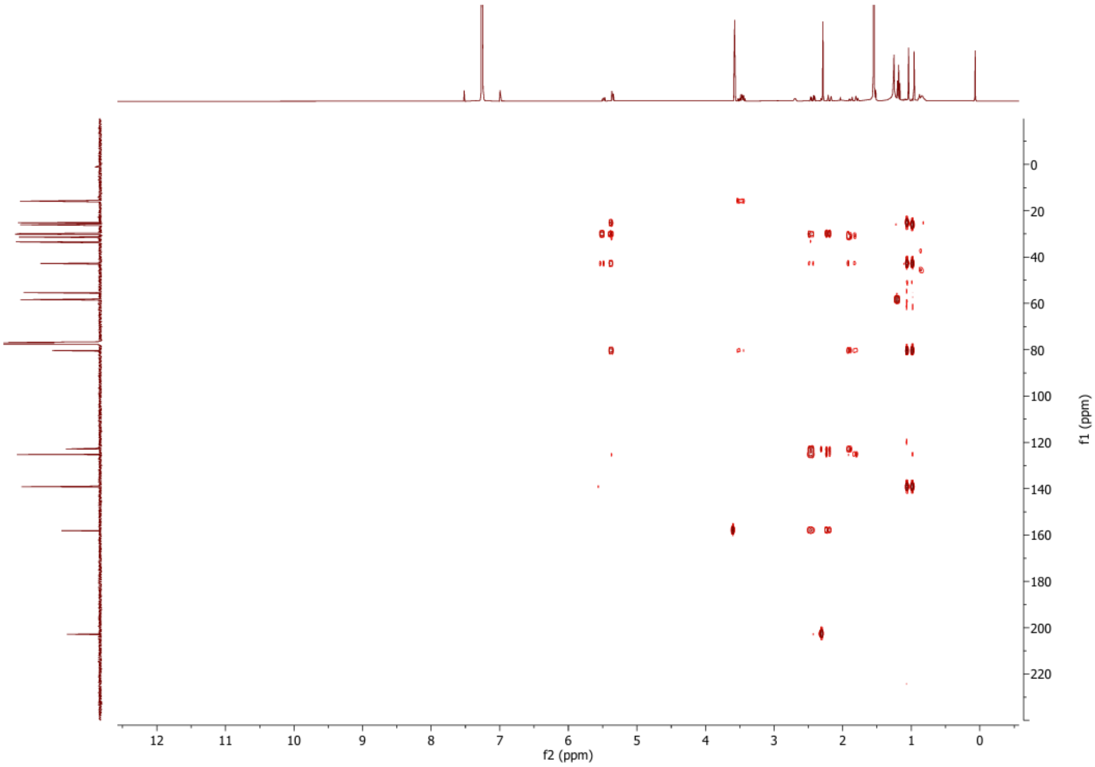

*10.2.3. 1-[(1S,5S)-1-Isopropoxy-3-methoxy-8,8-dimethylbicyclo[3.3.1]nona-2,6-dien-2-yl]ethan-1-one (**4c**)*

^{1}H NMR (400 MHz, $CDCl_3$):

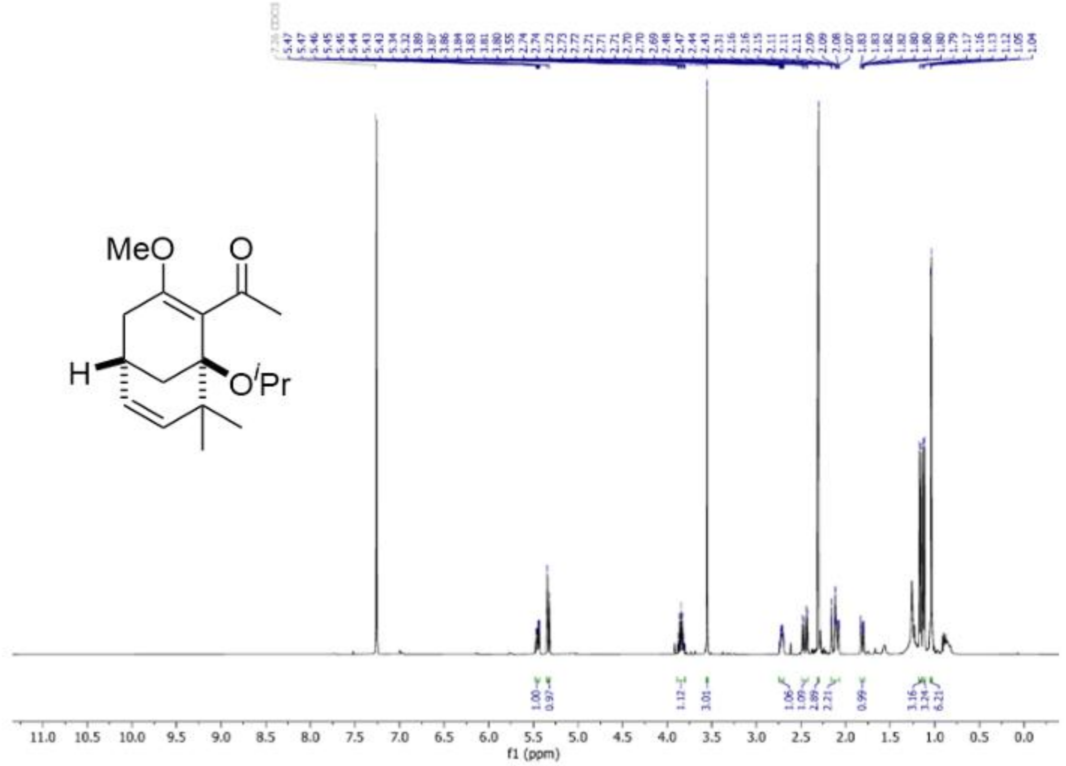

^{13}C NMR (101 MHz, $CDCl_3$):

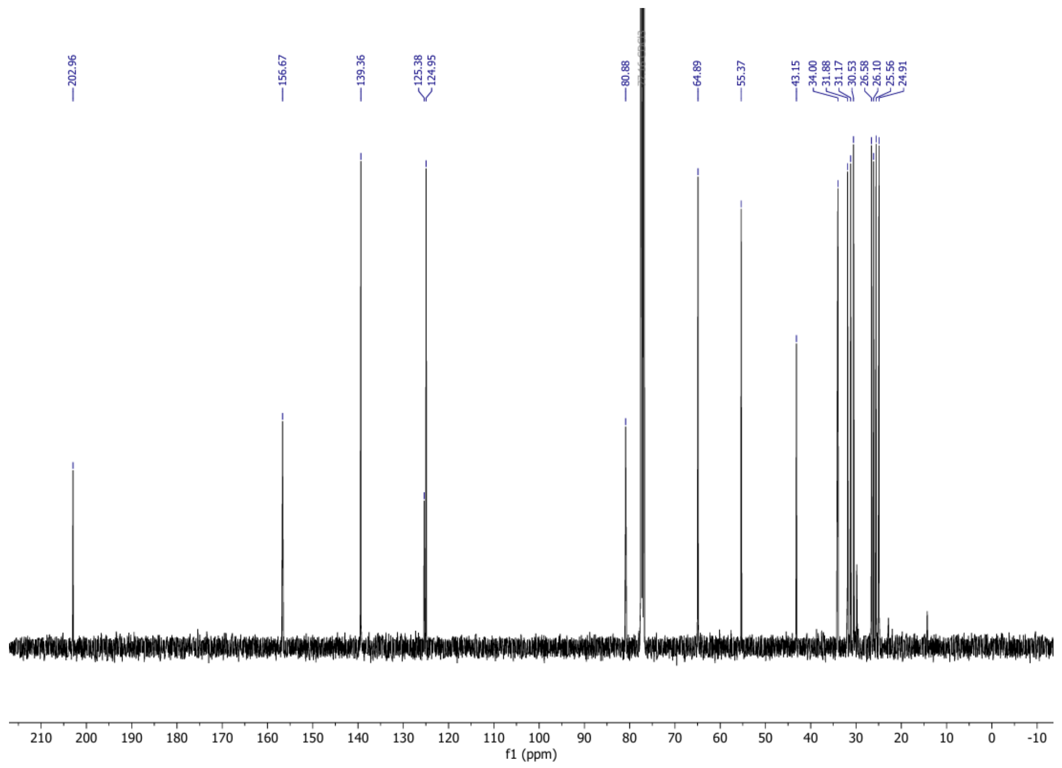

10.2.4. *1-{(1S,5S)-1-[(2,4-Dimethylpentan-3-yl)oxy]-3-methoxy-8,8-dimethylbicyclo[3.3.1]-nona-2,6-dien-2-yl}ethan-1-one (**4d**)*

^{1}H NMR (400 MHz, $CDCl_3$):

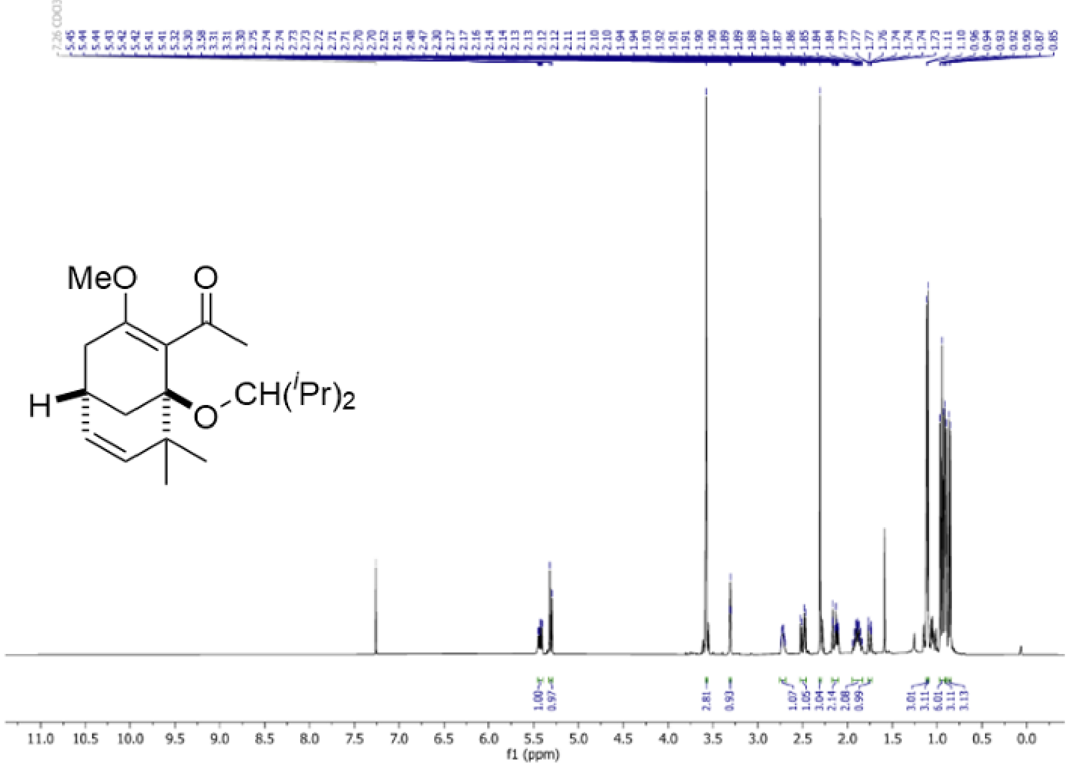

^{13}C NMR (101 MHz, $CDCl_3$):

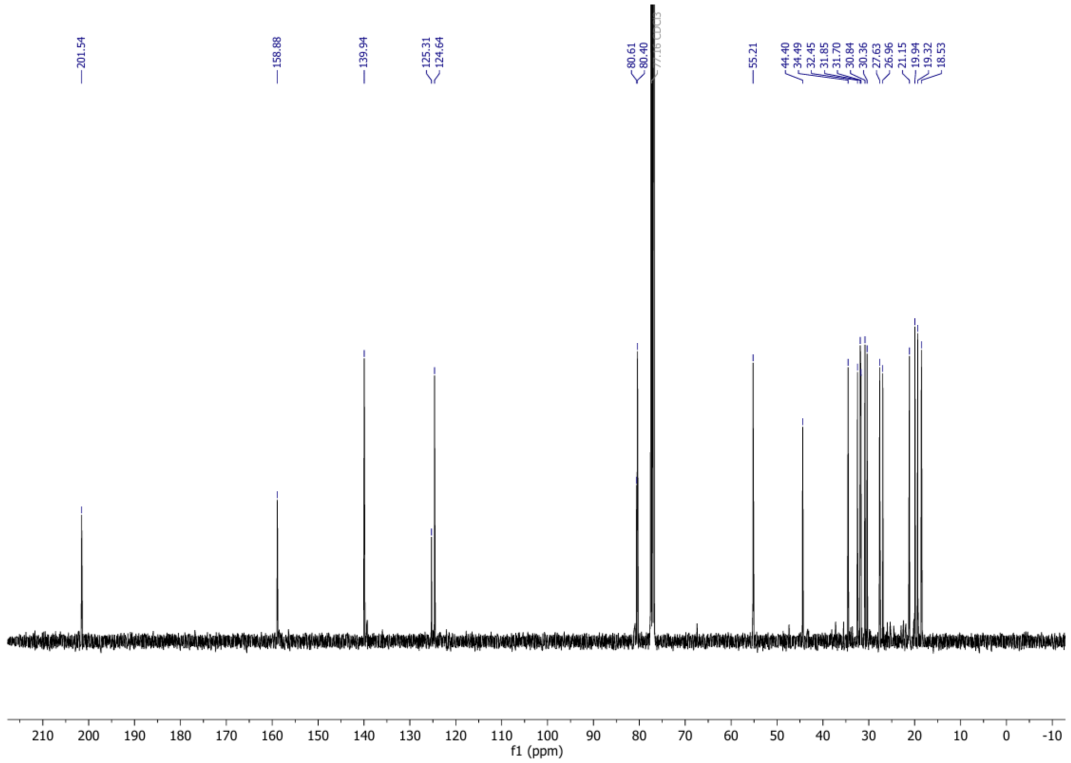

*10.2.5. 1-{(1S,5S)-1-[(4',4'-Dimethylcyclohexyl)oxy]-3-methoxy-8,8-dimethylbicyclo[3.3.1]nona-2,6-dien-2-yl}ethan-1-one (**4e**)*

^{1}H NMR (400 MHz, $CDCl_3$):

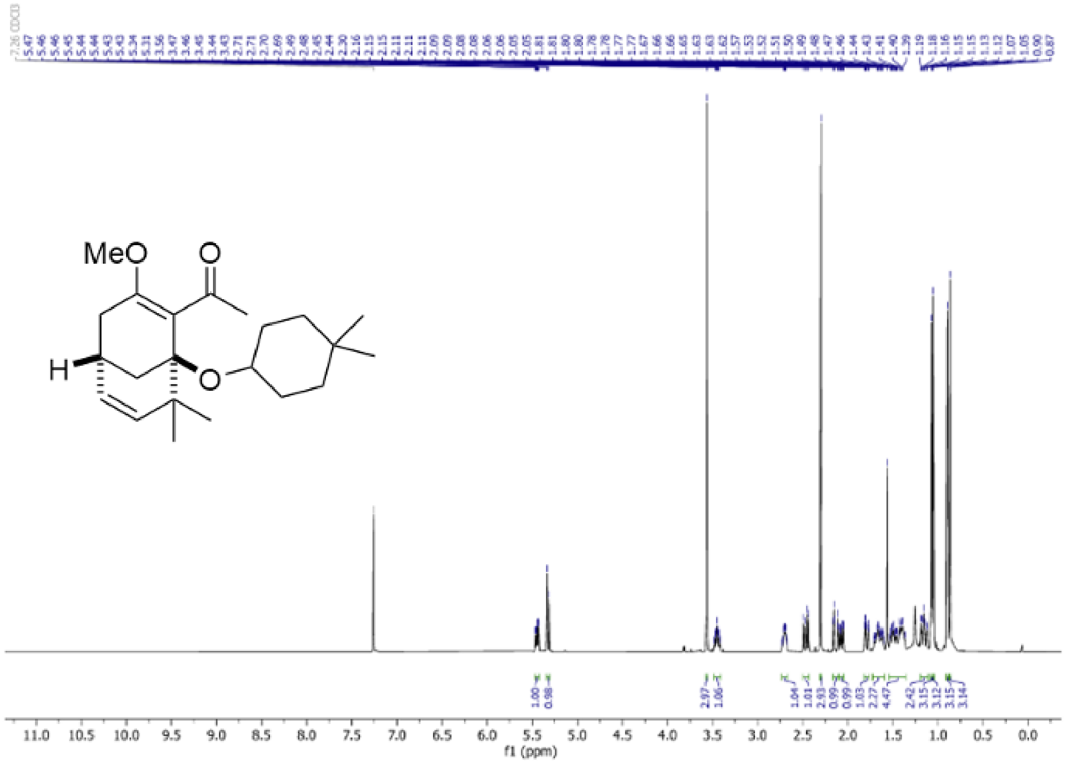

^{13}C NMR (101 MHz, $CDCl_3$):

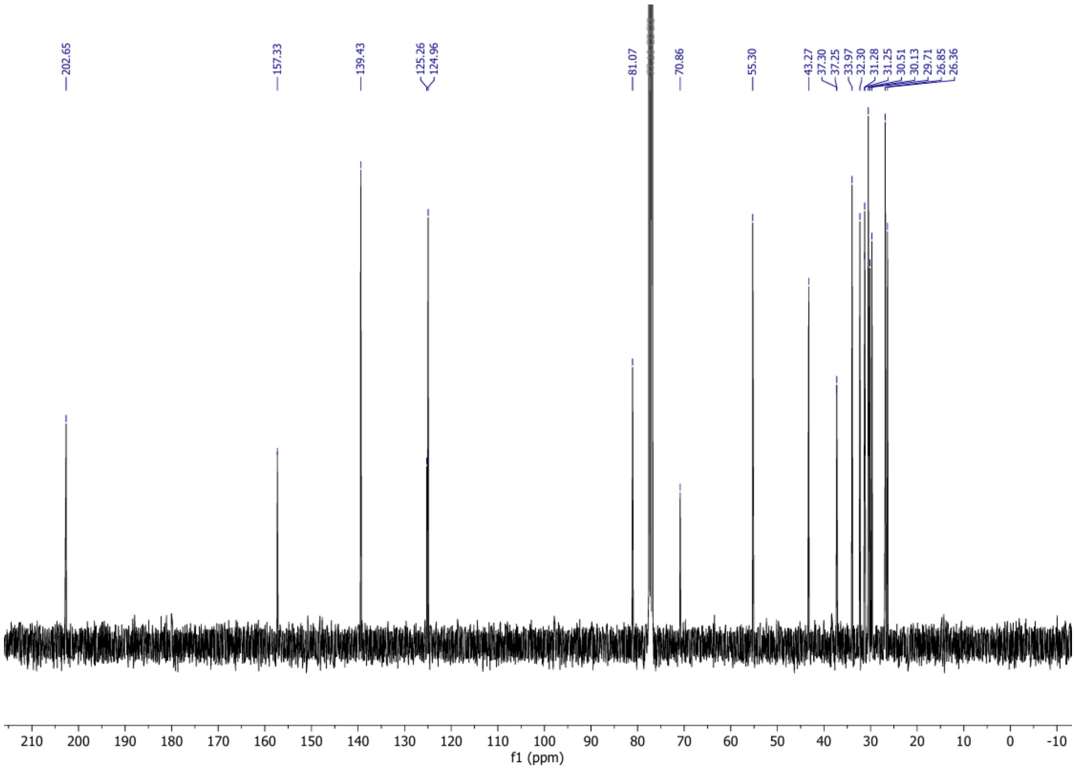

10.2.6. *1-(((1S,5S)-1-{[(1S,2S,5S)-2-Isopropyl-5-methylcyclohexyl]oxy}-3-methoxy-8,8-dimethylbicyclo[3.3.1]nona-2,6-dien-2-yl))ethan-1-one (**4f’**)*

^{1}H NMR (500 MHz, $CDCl_3$):

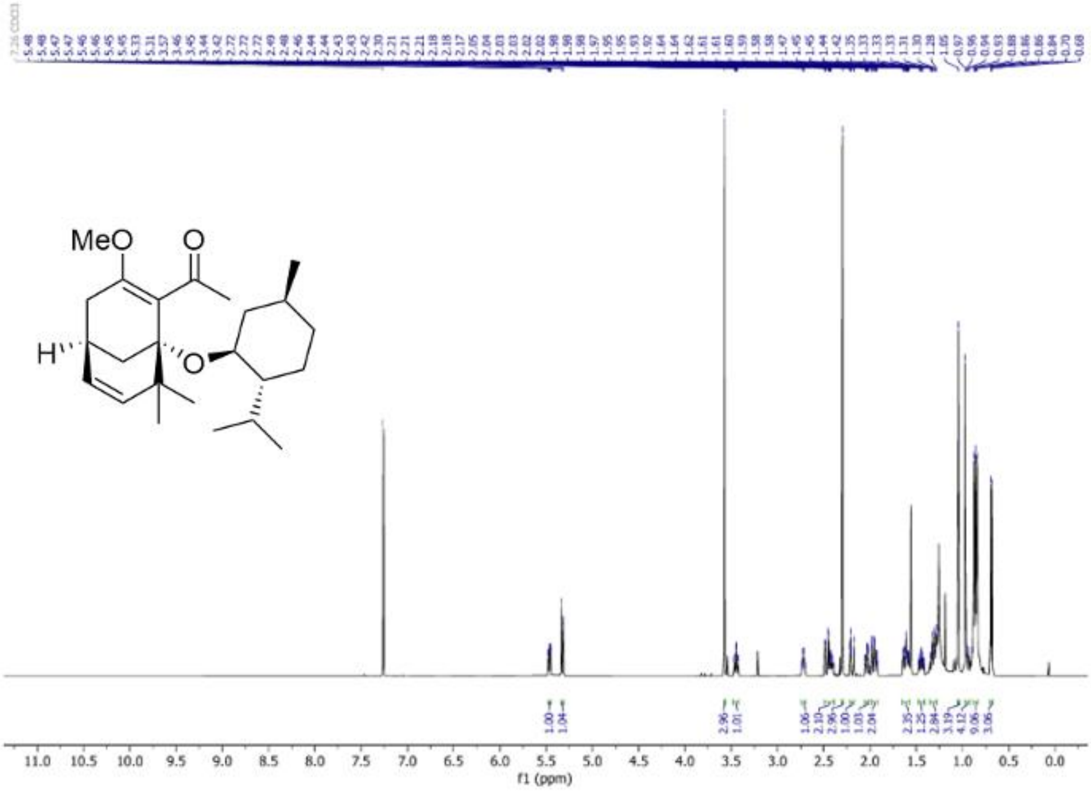

^{13}C NMR (101 MHz, $CDCl_3$):

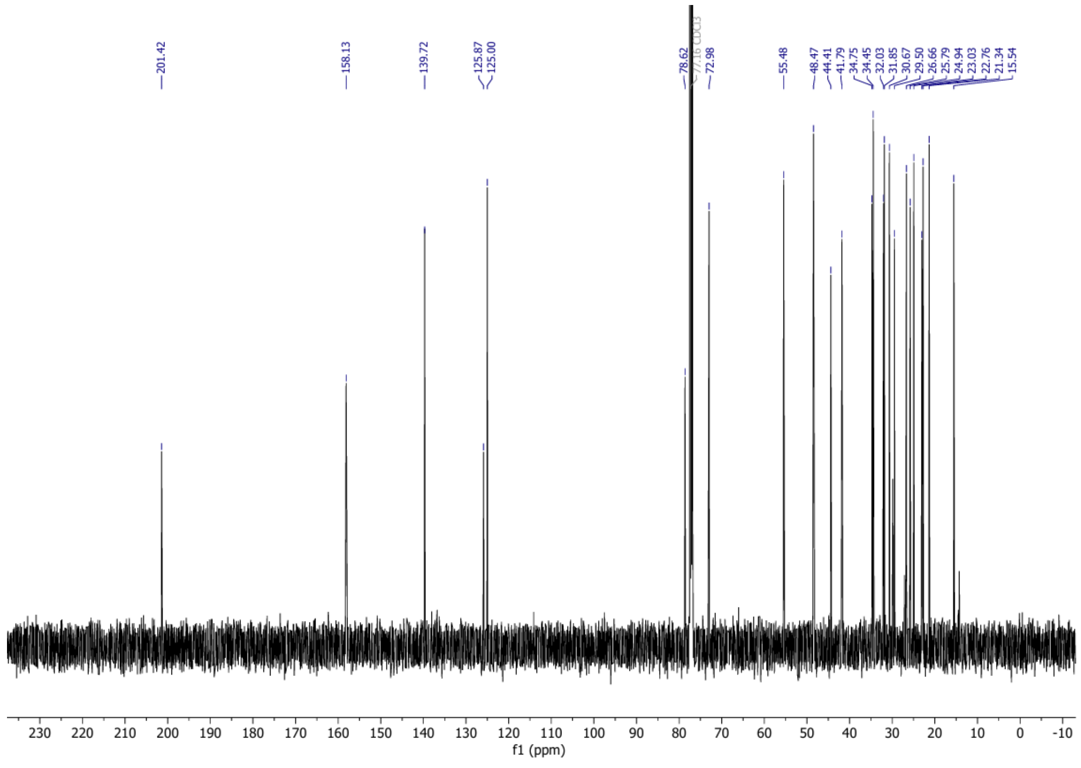

*10.2.7. 1-(((1R,5R)-1-{[(1S,2S,5S)-2-Isopropyl-5-methylcyclohexyl]oxy}-3-methoxy-8,8-dimethylbicyclo[3.3.1]nona-2,6-dien-2-yl))ethan-1-one (**4f’’**)*

^{1}H NMR (500 MHz, $CDCl_3$):

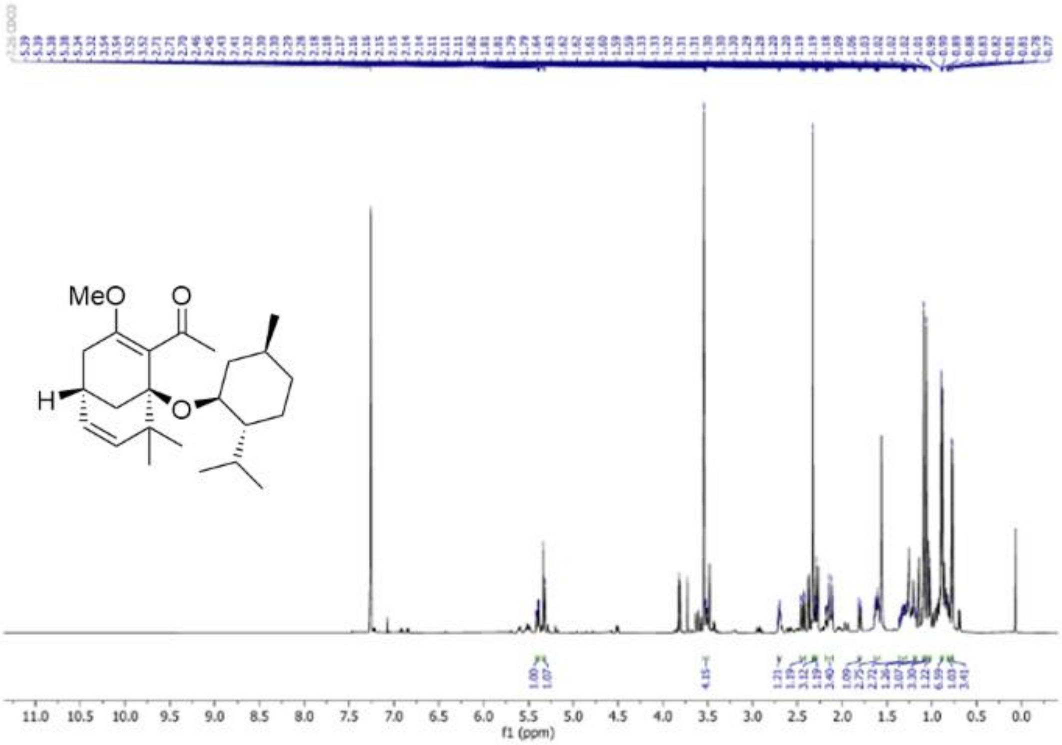

^{13}C NMR (101 MHz, $CDCl_3$):

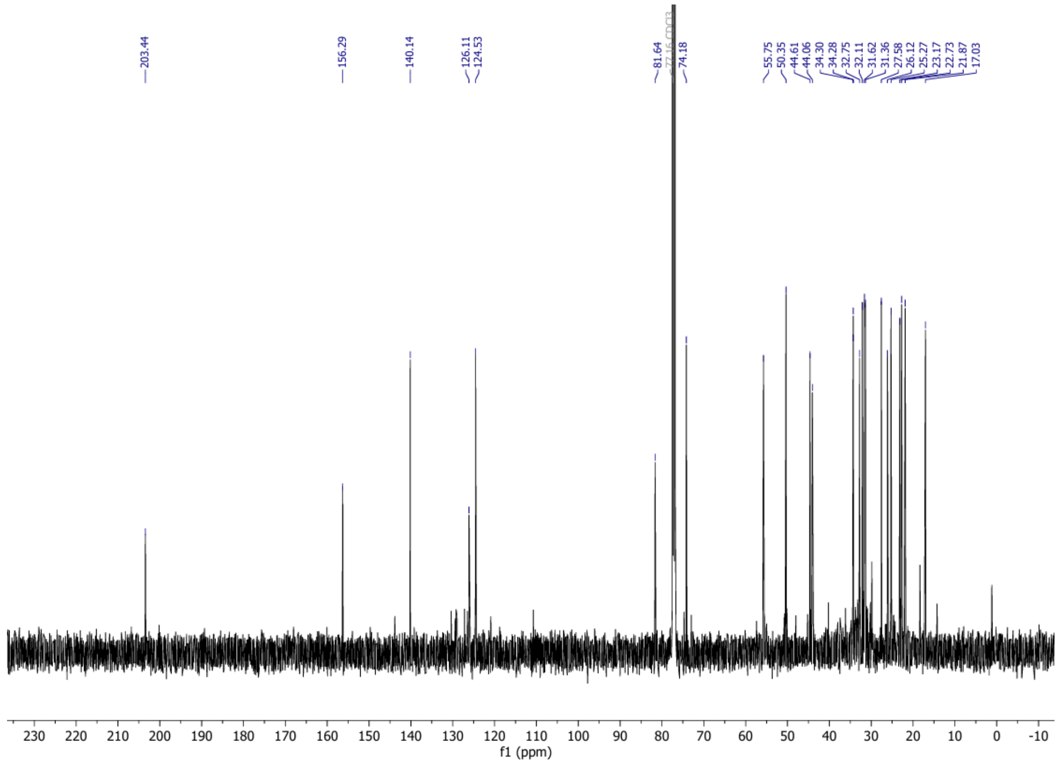

*10.2.8.(1S,5S)-2-Acetyl-3-methoxy-8,8-dimethylbicyclo[3.3.1]nona-2,6-dien-1-yl benzoate (**9**)*

^{1}H NMR (400 MHz, $CDCl_3$):

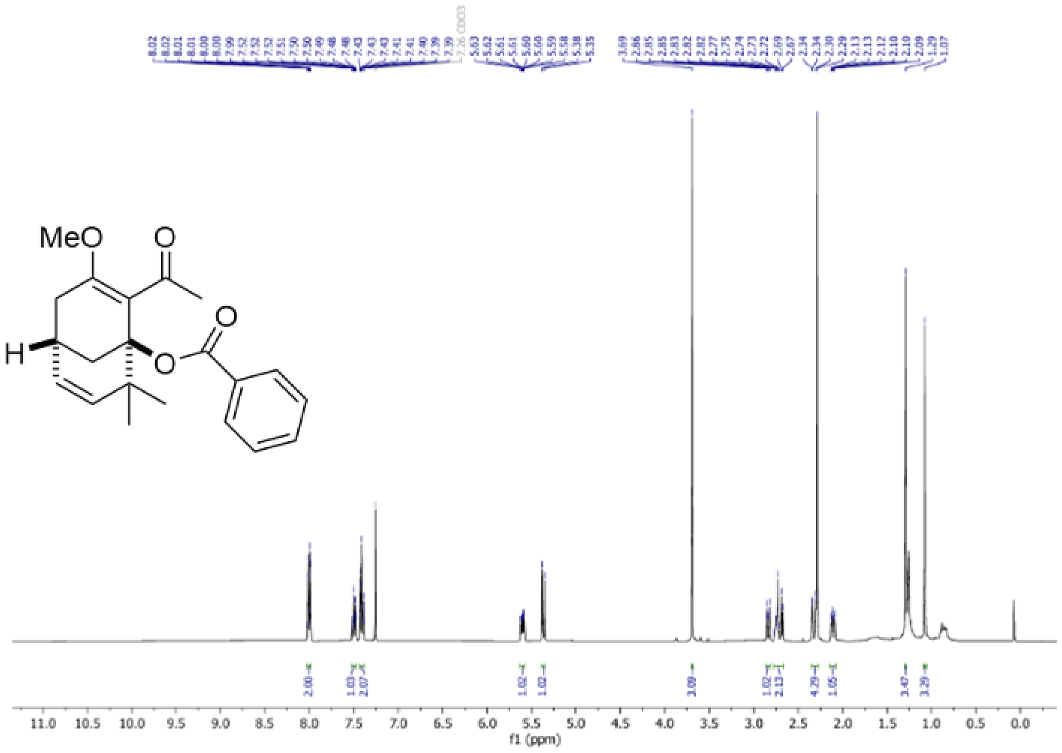

^{13}C NMR (101 MHz, $CDCl_3$):

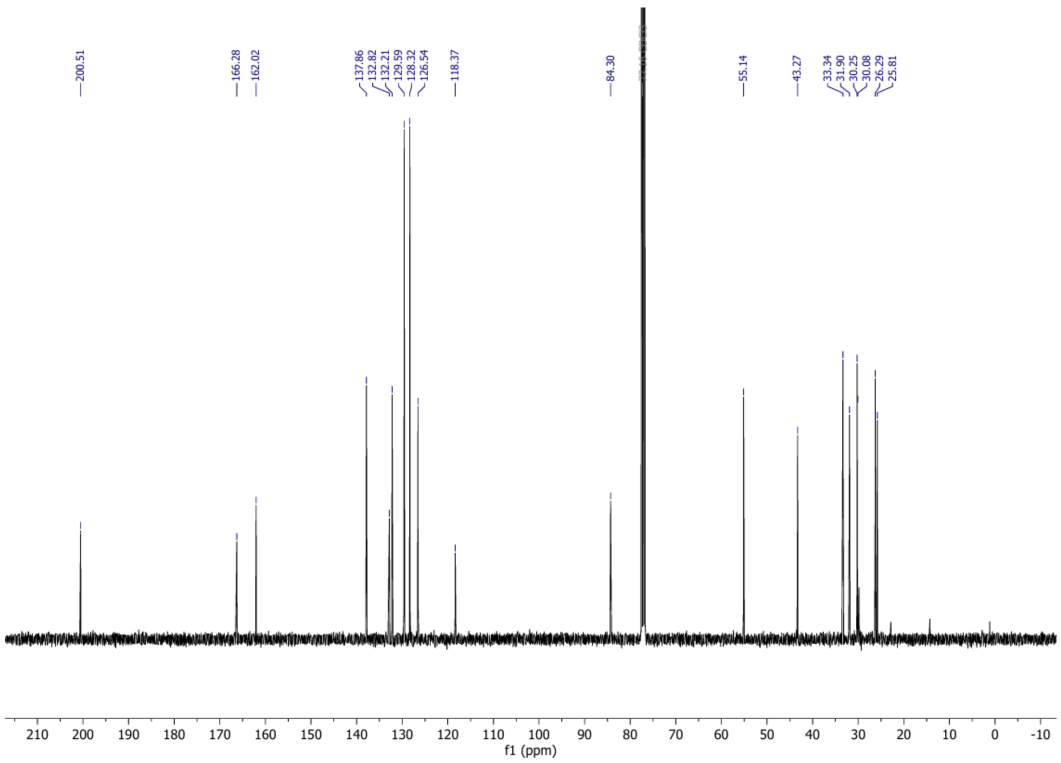

*10.2.9. 1-{(1S,5S)-1-[(Adamantan-1′-yl)thio]-3-methoxy-8,8-dimethylbicyclo[3.3.1]nona-2,6-dien-2-yl}ethan-1-one (**10**)*

^{1}H NMR (400 MHz, $CDCl_3$):

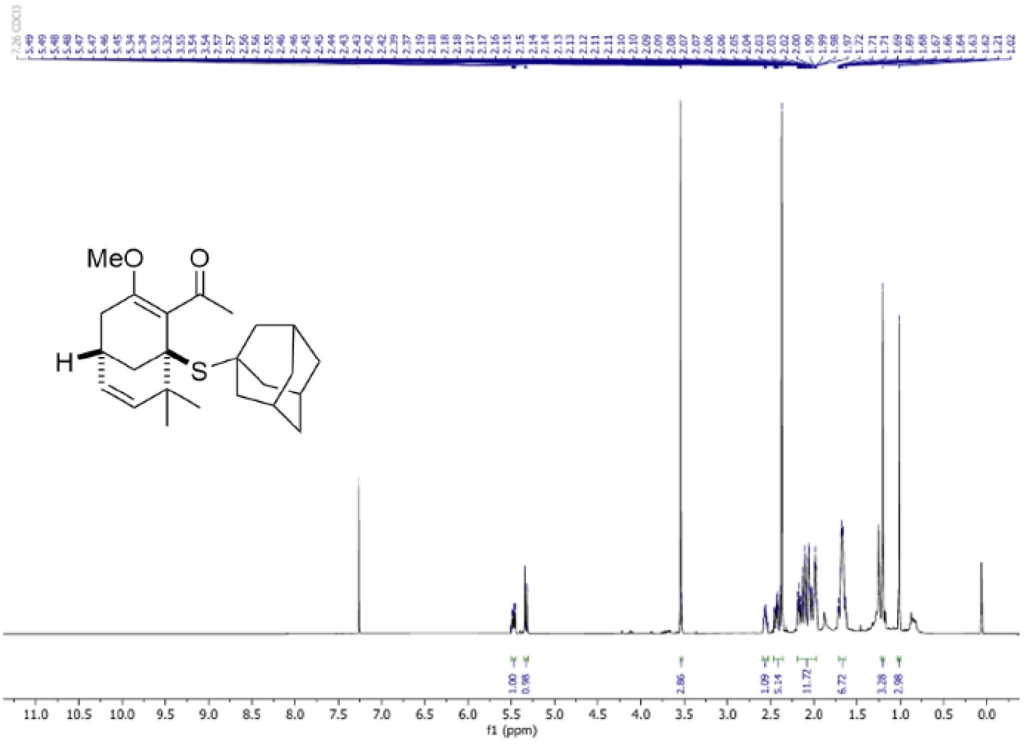

^{13}C NMR (101 MHz, $CDCl_3$):

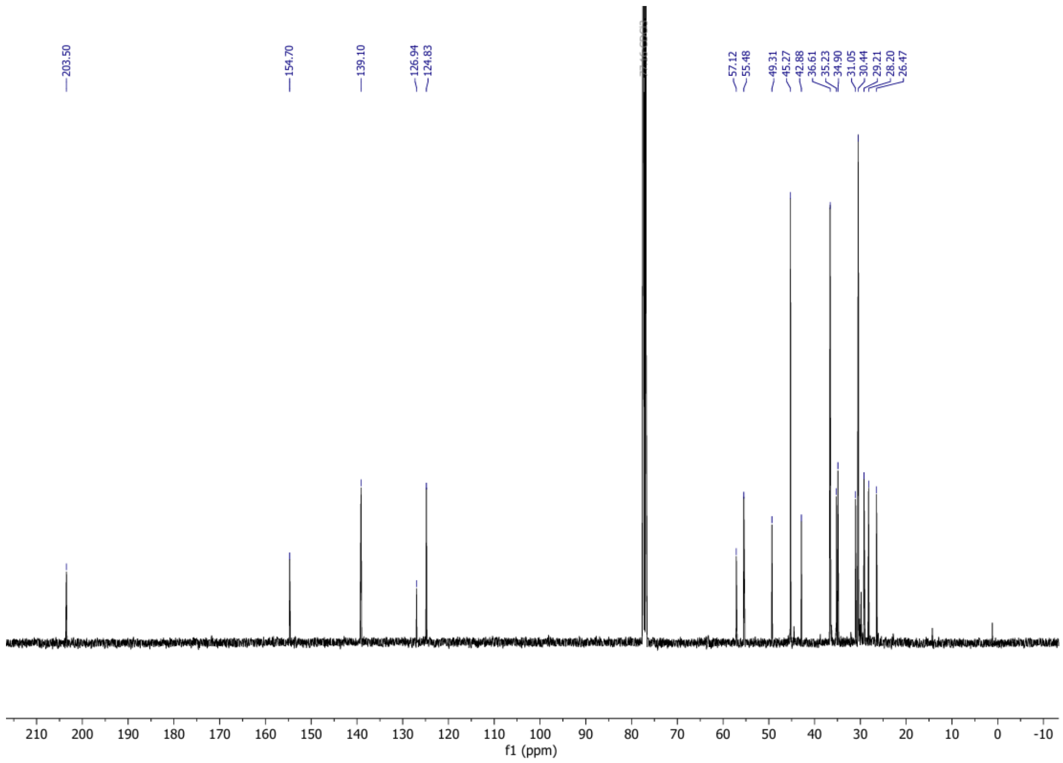

*10.2.10. 1-[(1S,5R)-3-Methoxy-8,8-dimethyl-1-(neopentylamino)bicyclo[3.3.1]nona-2,6-dien-2-yl]ethan-1-one (**12**)*

^{1}H NMR (400 MHz, $CDCl_3$):

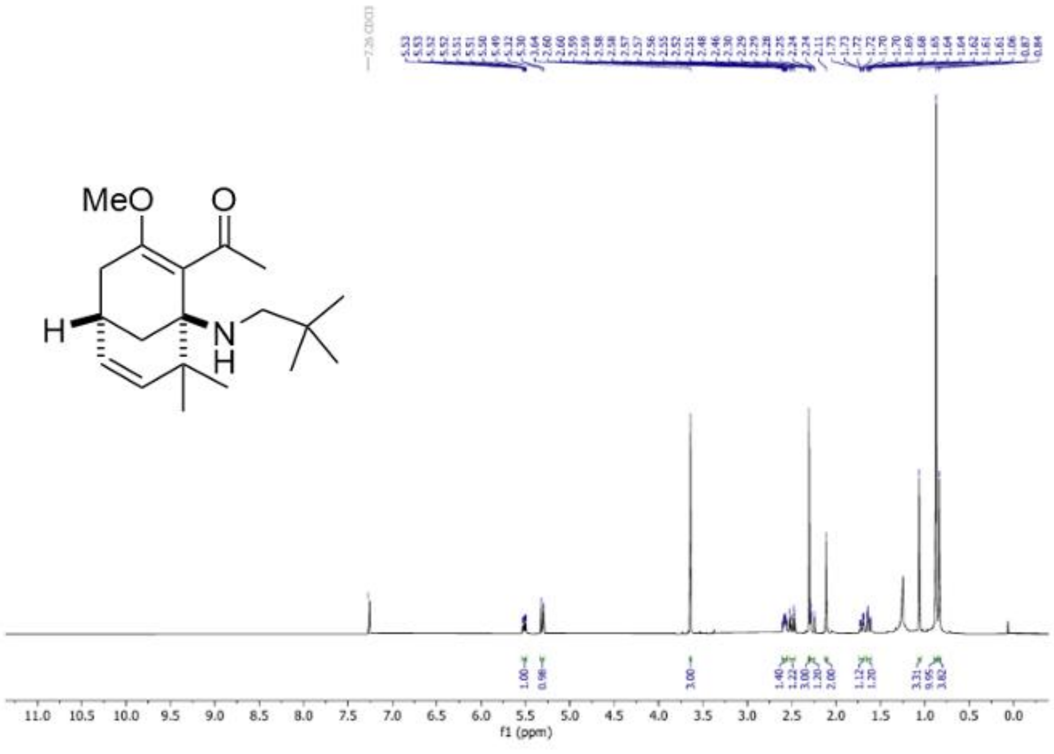

^{13}C NMR (101 MHz, $CDCl_3$):

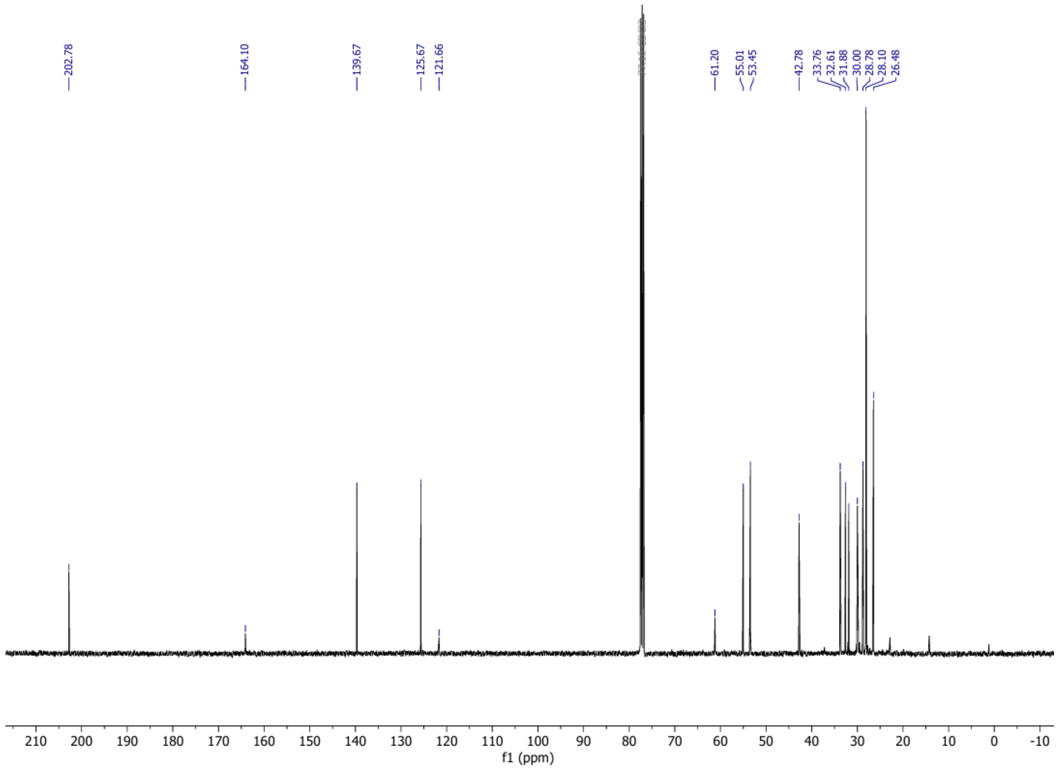

10.3. Photoproducts Resulting from the Irradiation of 2-Methoxybenzoic Acid Methyl Ester

*10.3.1. 1-[4-Methoxy-7-(propan-2-ylidene)bicyclo[2.2.2]octa-2,5-dien-1-yl]ethan-1-one (**13**)*

^1H NMR (400 MHz, $CDCl_3$):

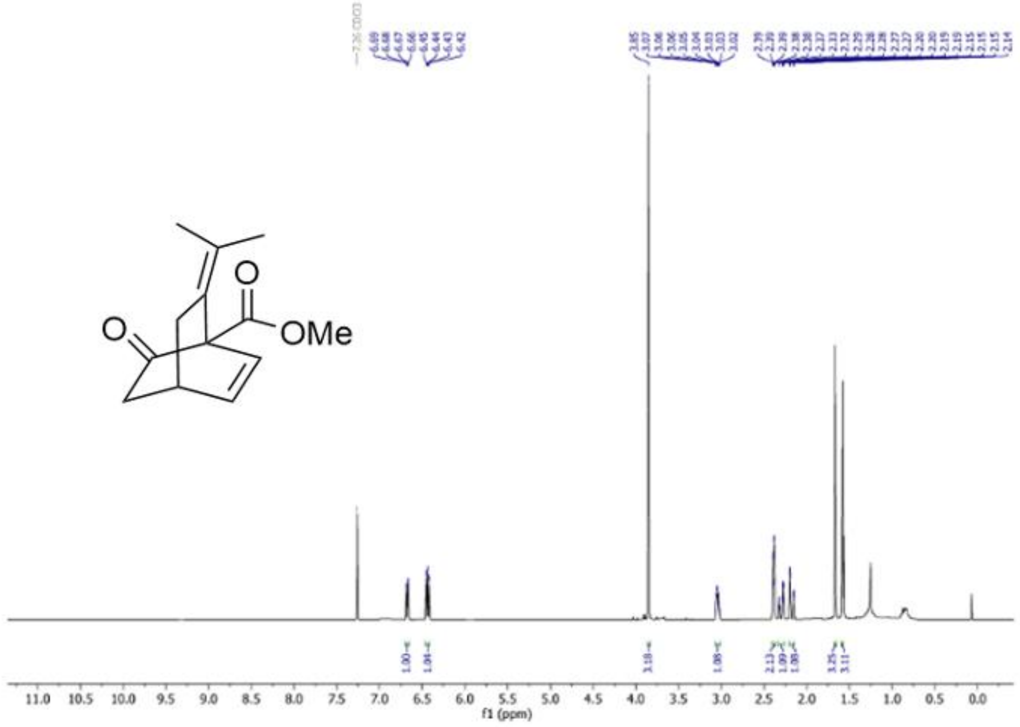

^{13}C NMR (101 MHz, $CDCl_3$):

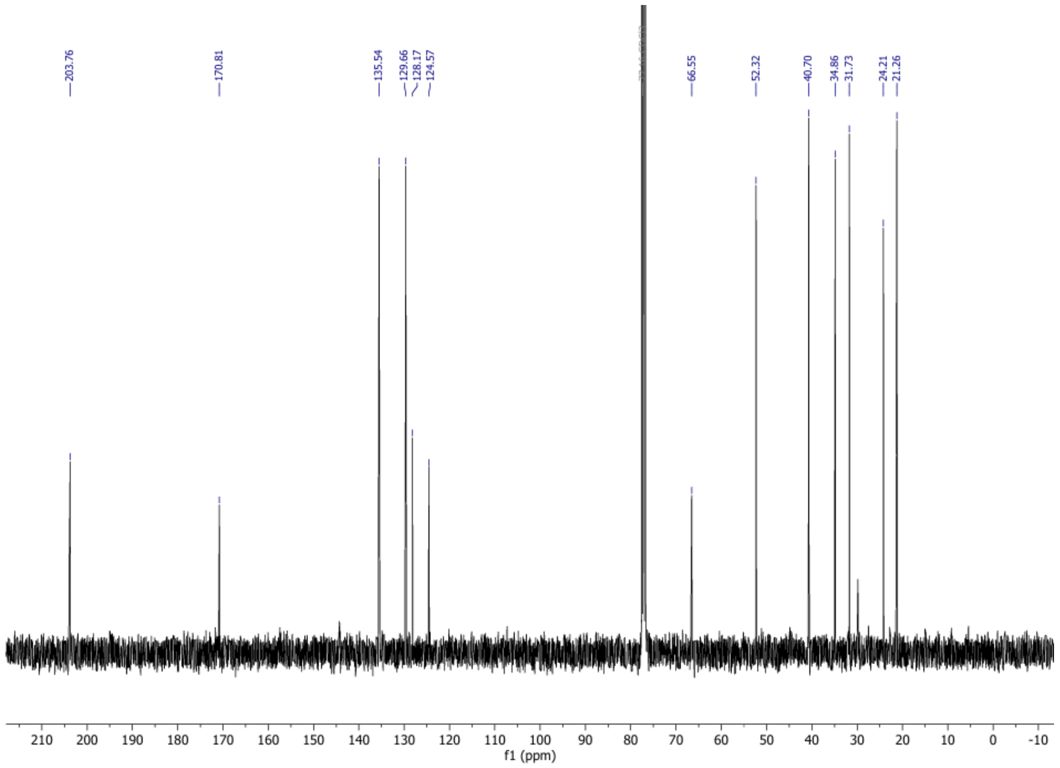

COSY spectrum:

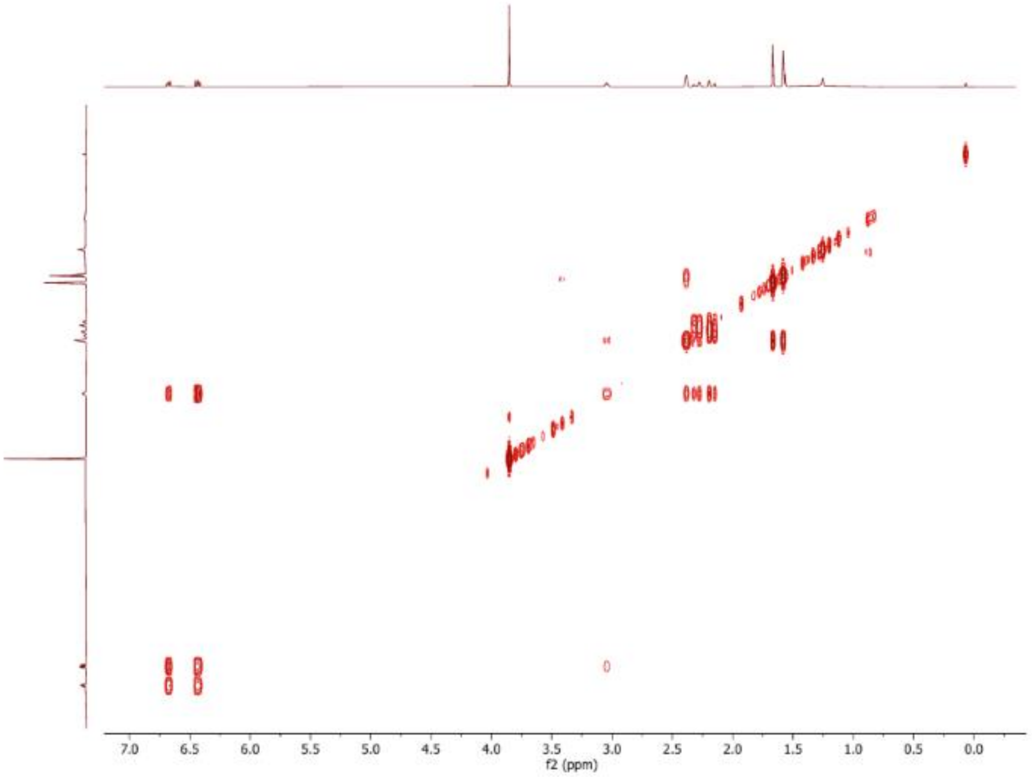

HSQC spectrum:

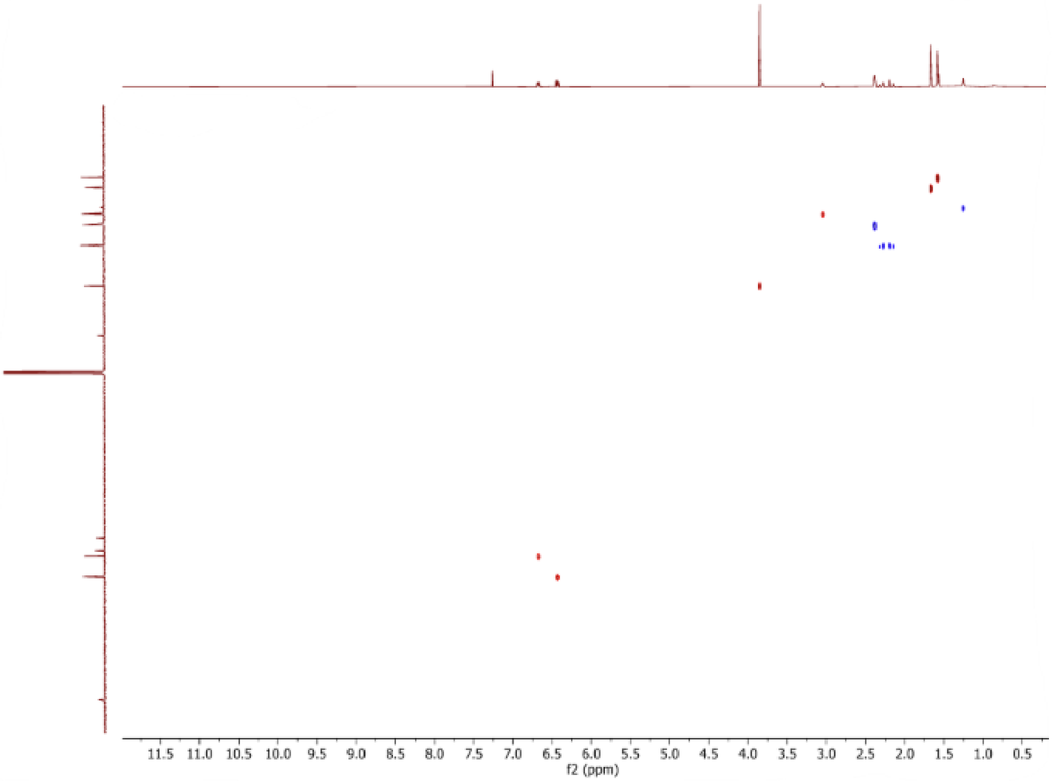

HMBC spectrum:

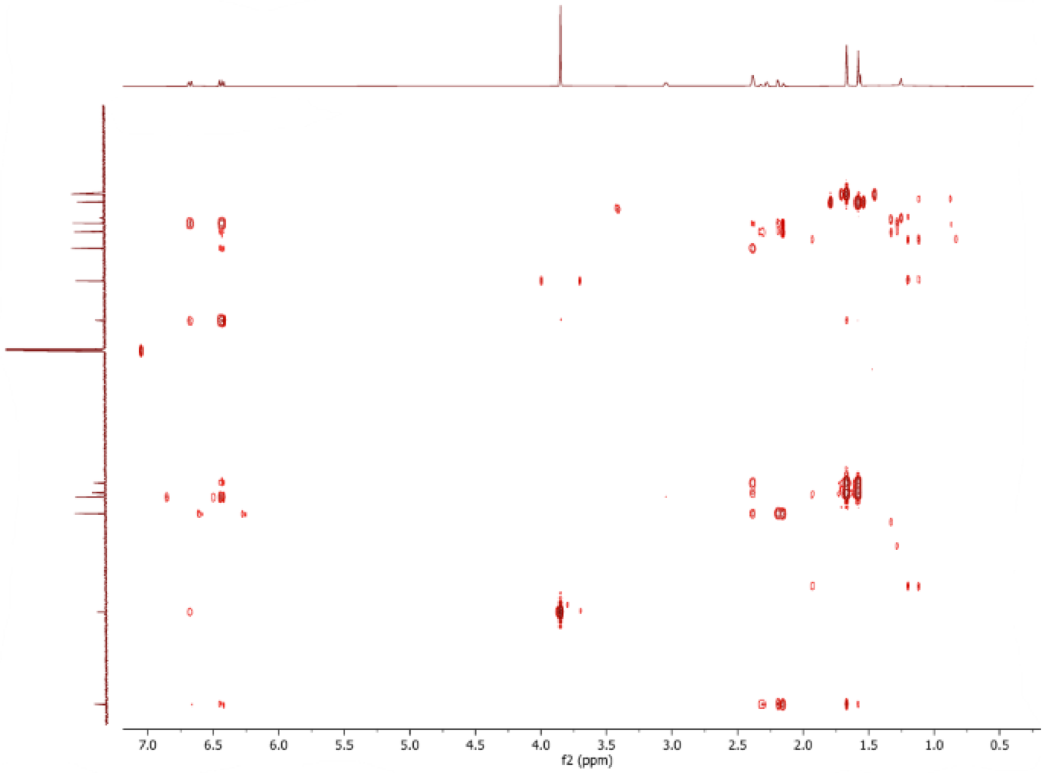

*10.3.2. Methyl (2a^{1}R,4aS)-2a^1-methoxy-2,2-dimethyl-1-methylene-2,2a,2a^1,2b-tetrahydrocyclopropa[cd]pentalene-4a(1H)-carboxylate (**14**)*

^{1}H NMR (400 MHz, $CDCl_3$):

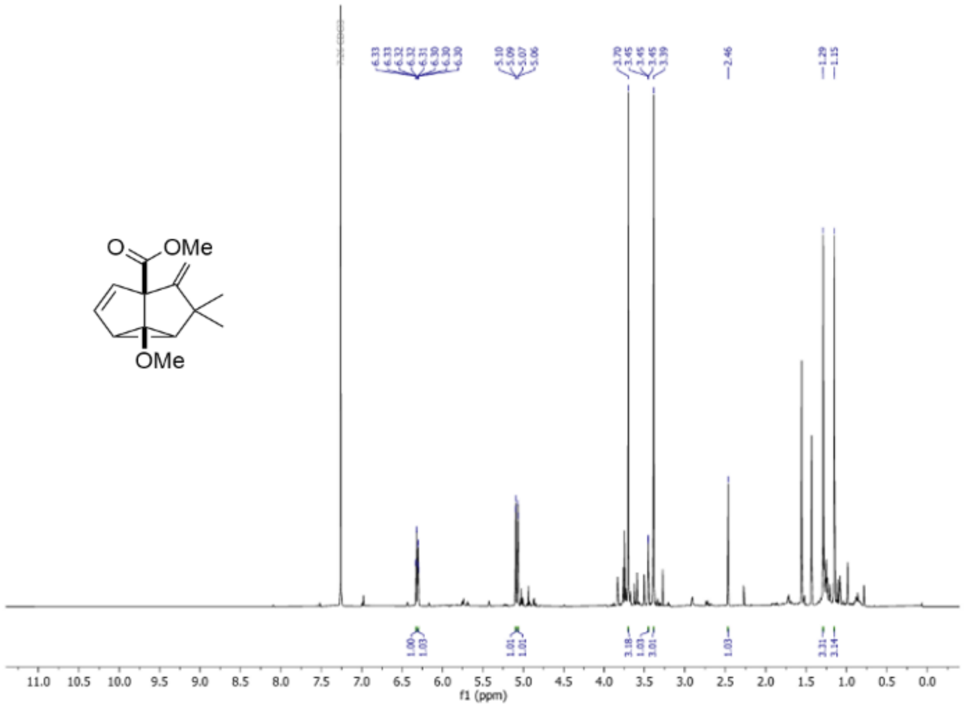

^{13}C NMR (126 MHz, $CDCl_3$):

Product was prone to decomposition before measurement of the ^{13}C NMR spectrum.

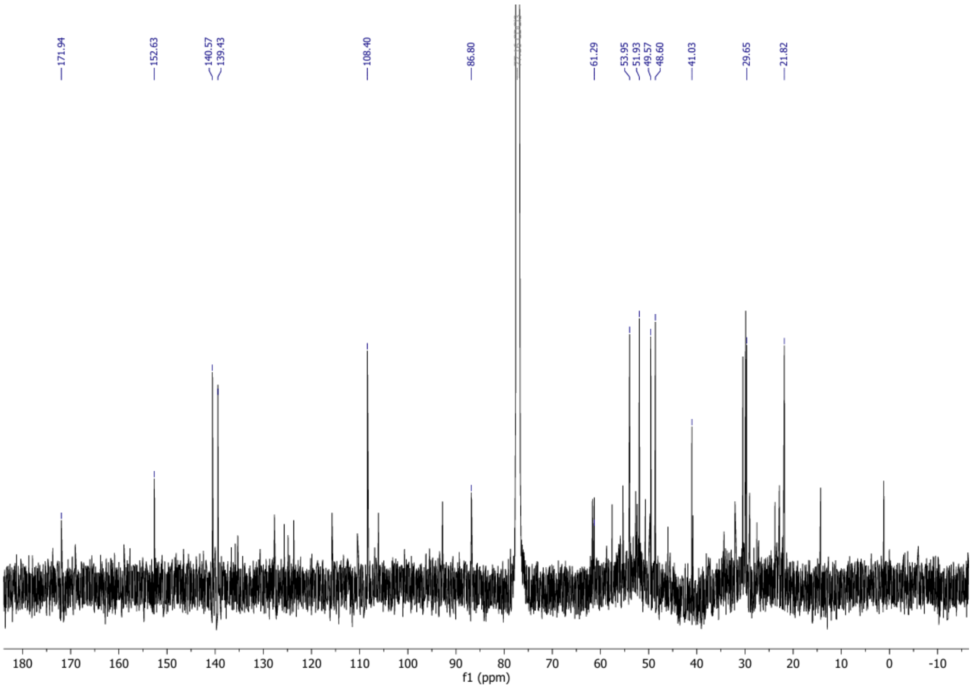

COSY spectrum:

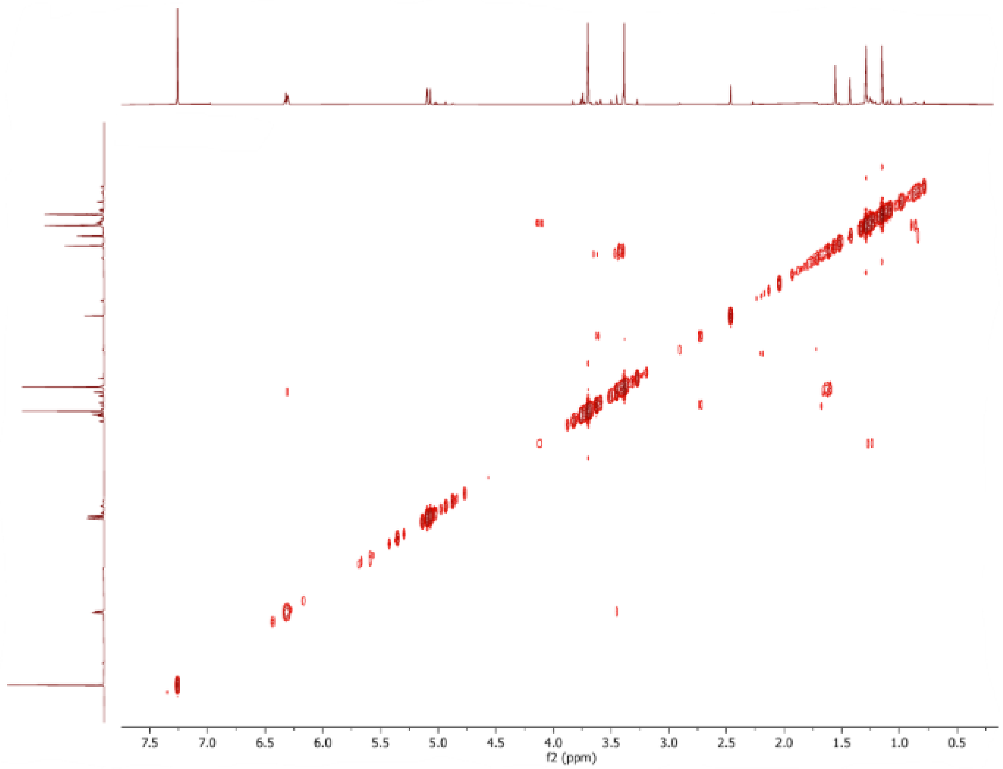

HSQC spectrum:

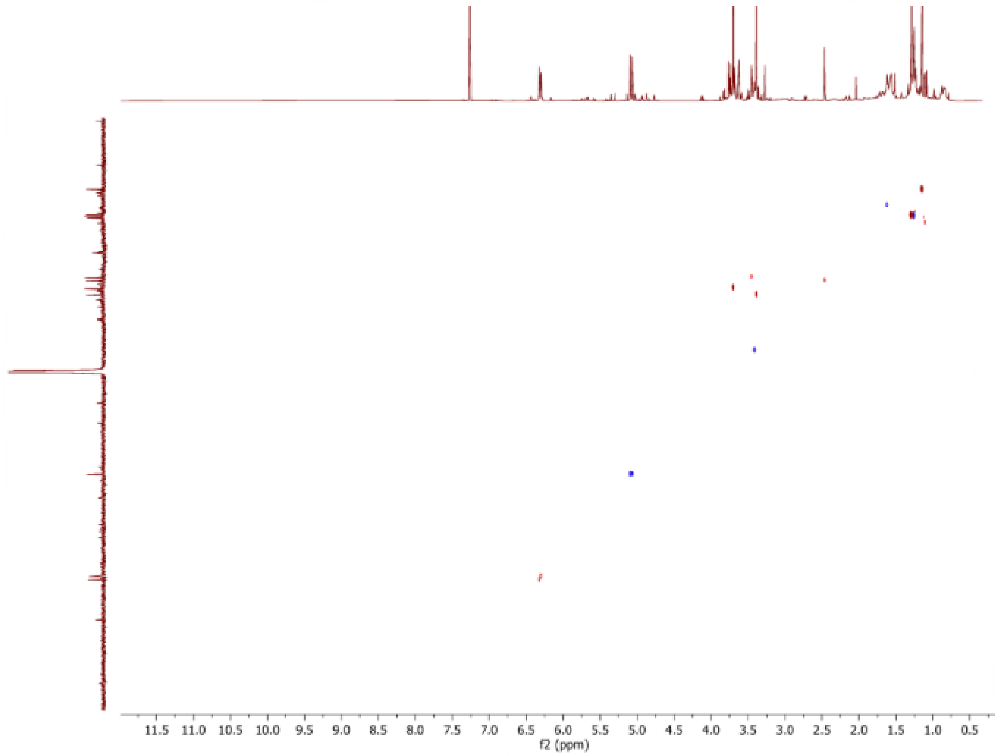

HMBC spectrum:

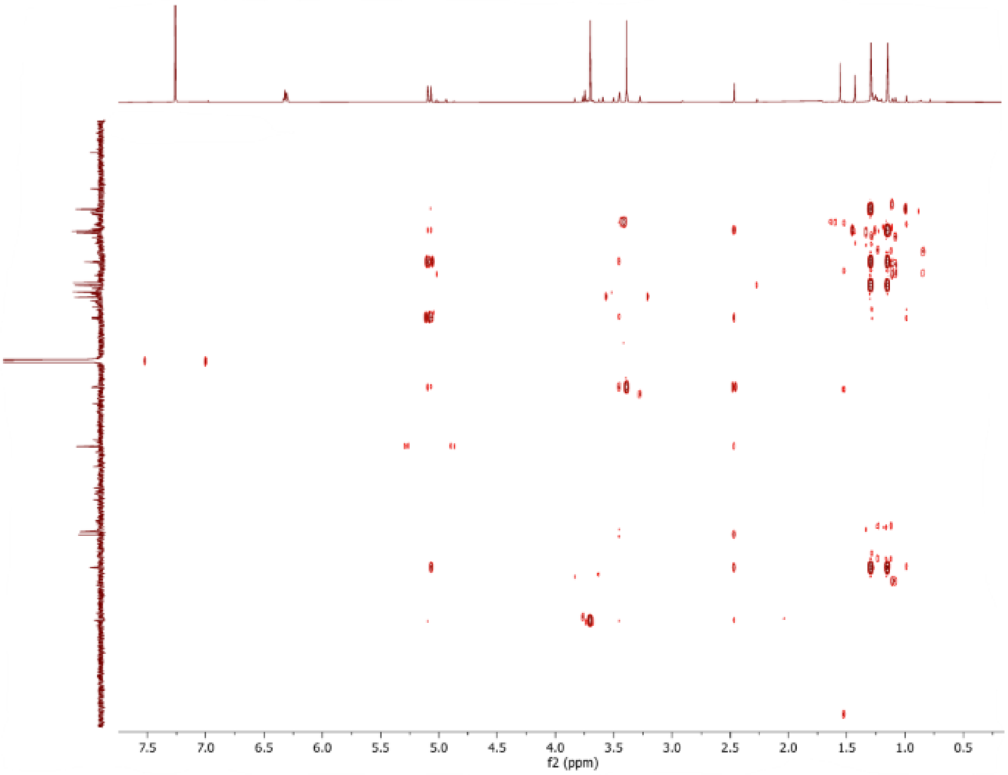

*For better illustration of the corresponding ^{13}C NMR signals, a spectrum of a mixture together with ortho-photocycloaddition compound **16** is given, which was only barely prone to decomposition (only signals of compound **14** are marked):*

^{13}C NMR (101 MHz, $CDCl_3$):

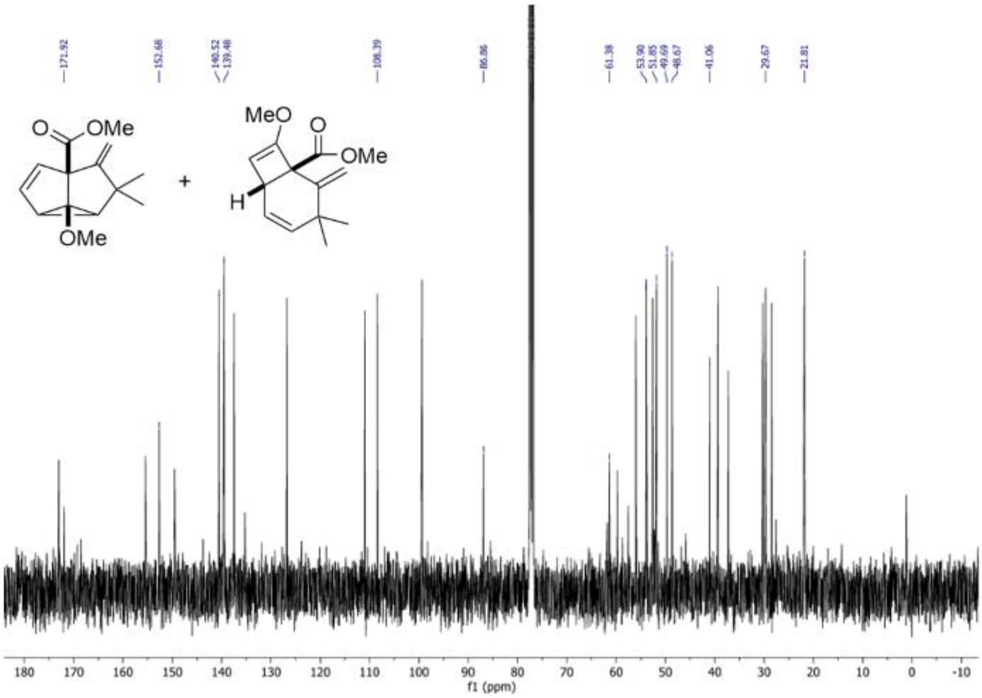

*10.3.3. Methyl (1R,6S)-2-methoxy-5,5-dimethyl-4-methylenebicyclo[4.2.0]octa-2,7-diene-3-carboxylate (**15**)*

^{1}H NMR (400 MHz, $CDCl_3$):

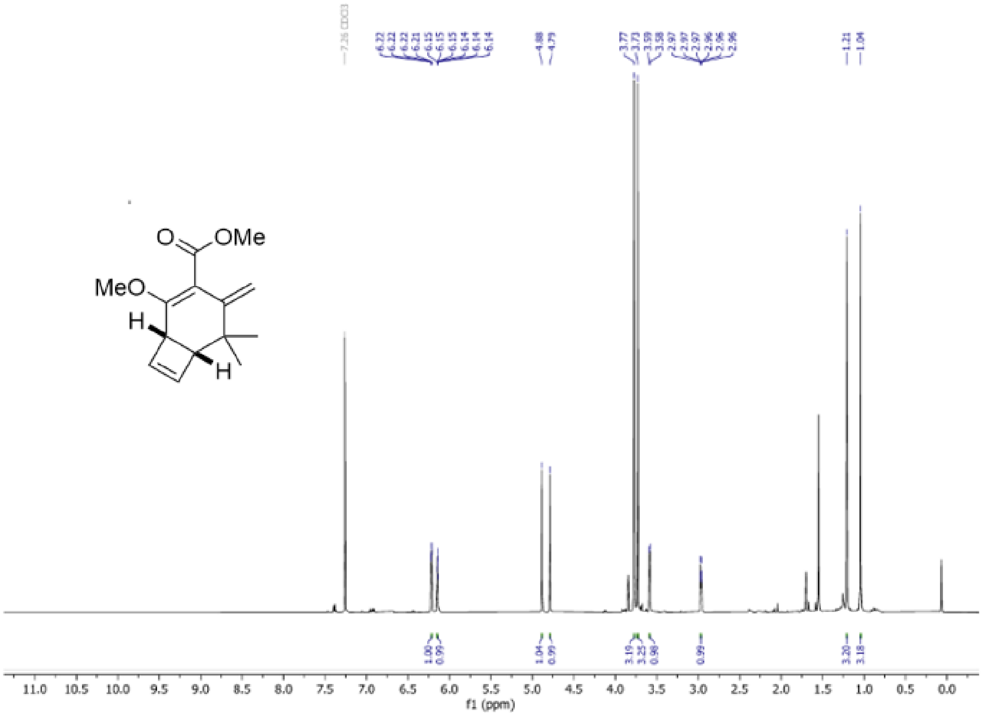

^{13}C NMR (126 MHz, $CDCl_3$):

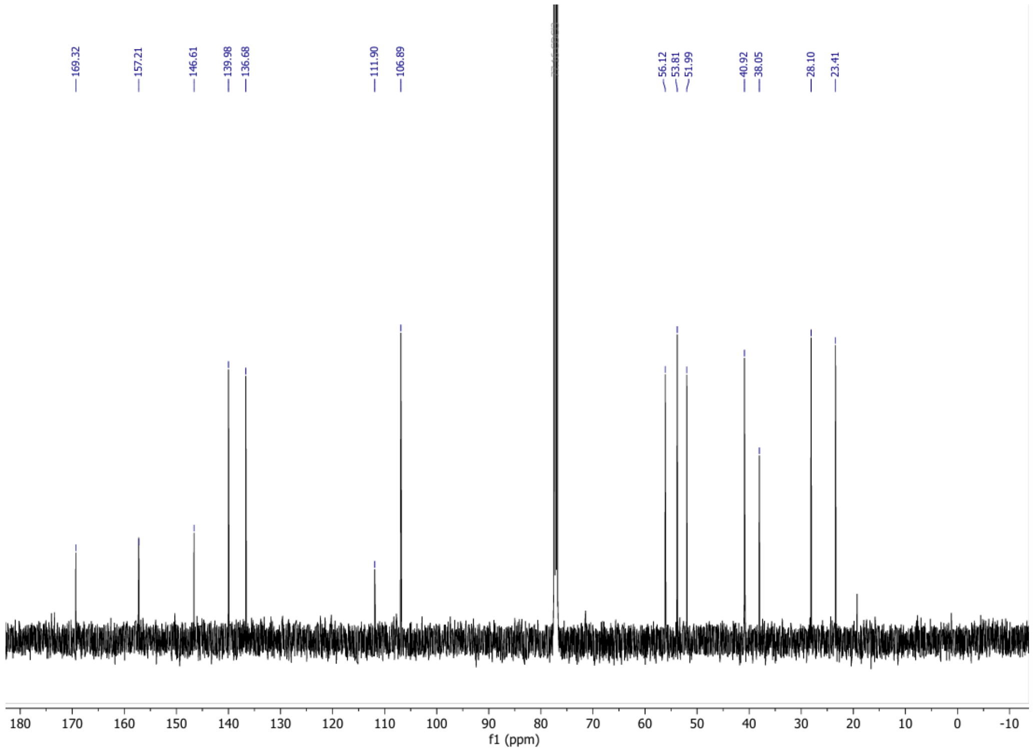

COSY spectrum:

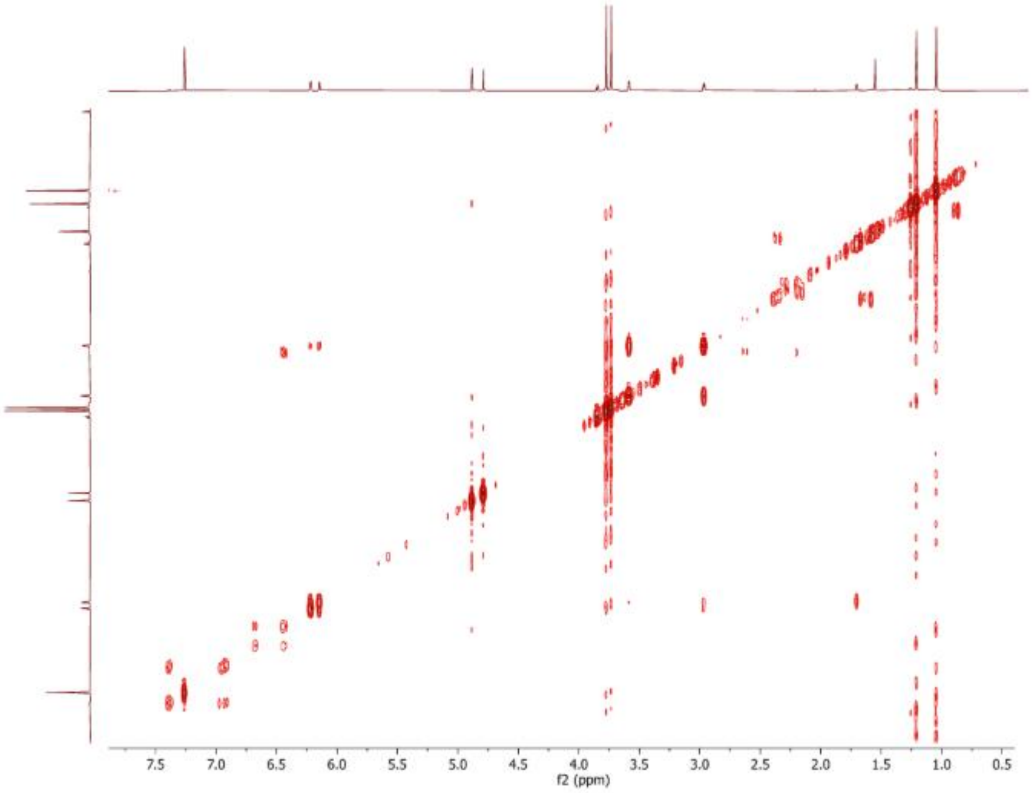

HSQC spectrum:

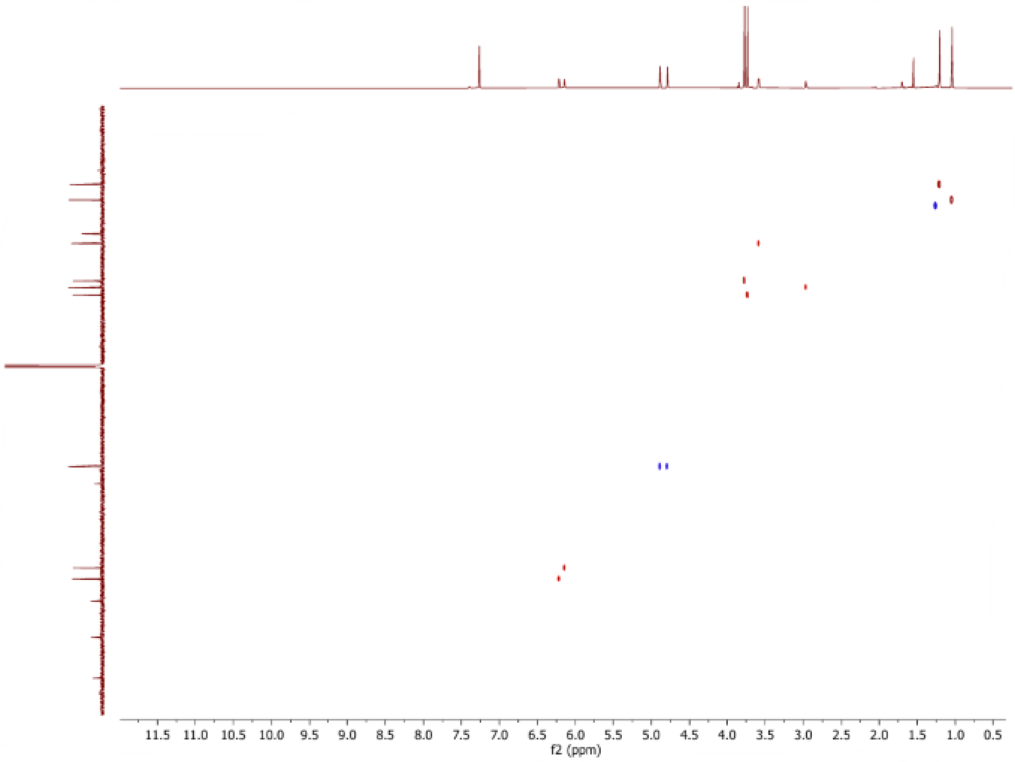

HMBC spectrum:

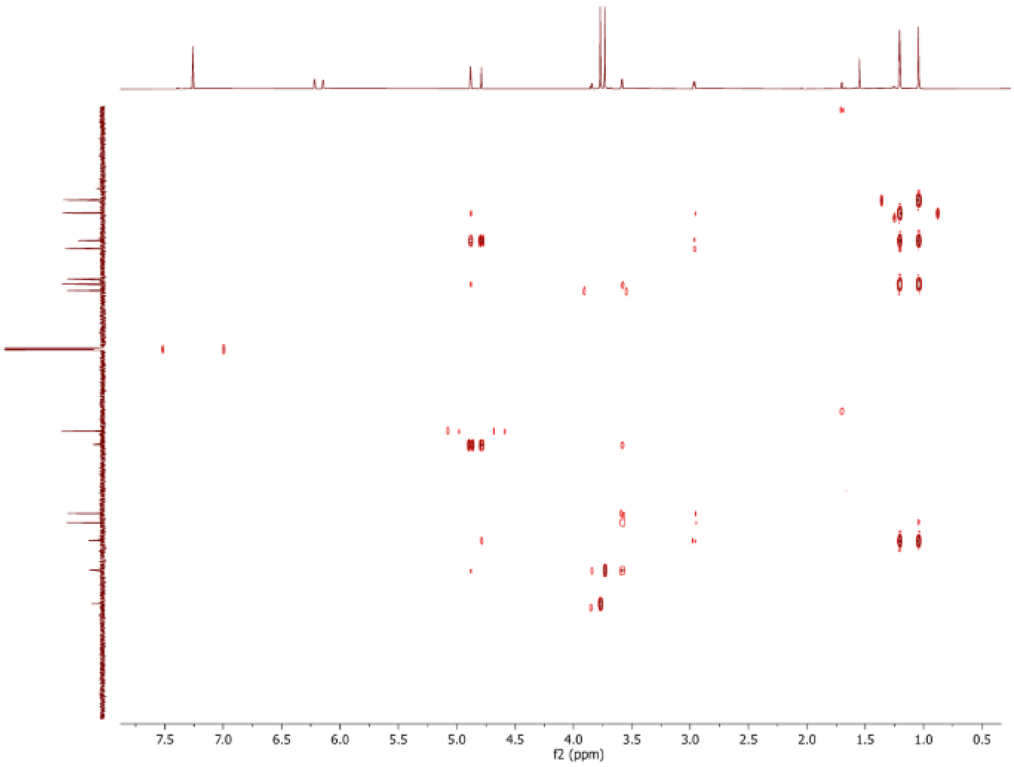

*10.3.4. Methyl (1S,6S)-8-methoxy-3,3-dimethyl-2-methylenebicyclo[4.2.0]octa-4,7-diene-1-carboxylate (**16**)*

^{1}H NMR (400 MHz, $CDCl_3$):

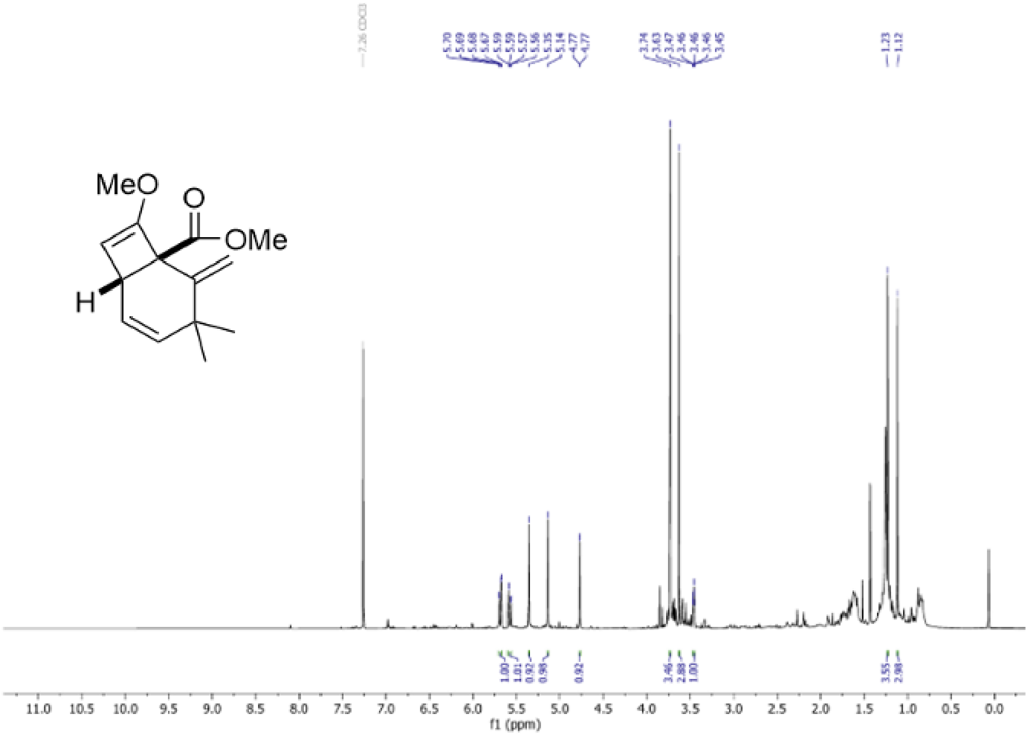

^{13}C NMR (101 MHz, $CDCl_3$):

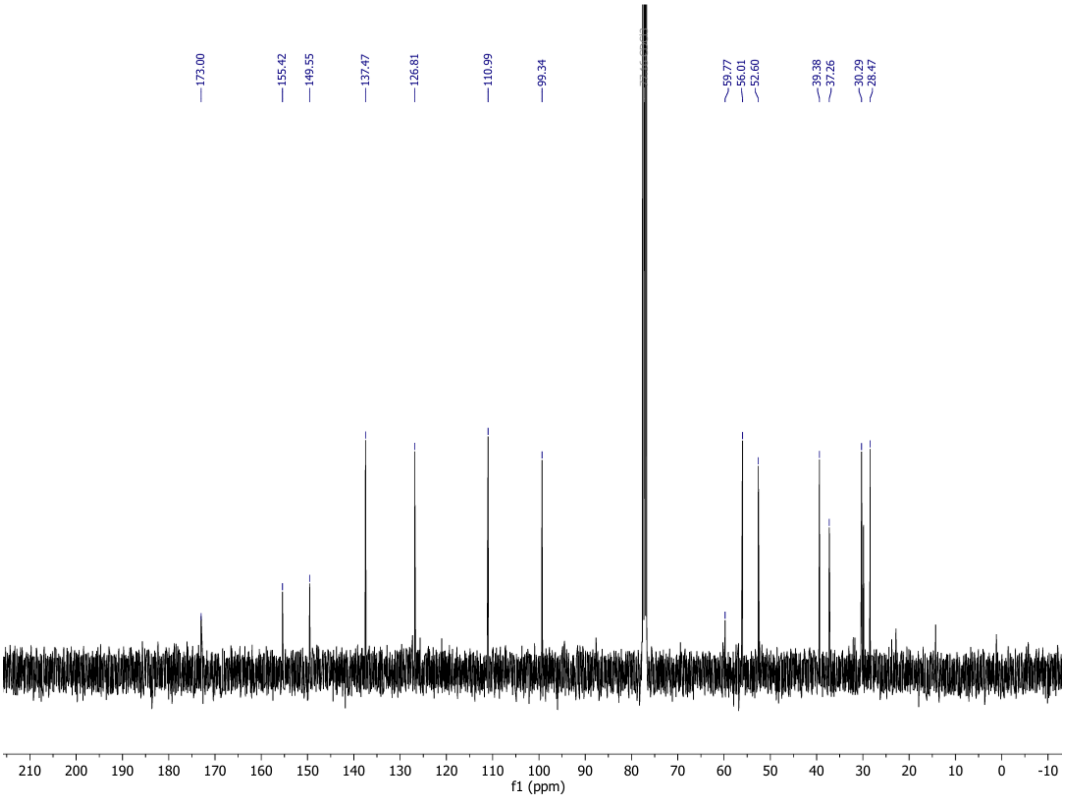

*Unfortunately, compound **16** was not stable enough for 2D analysis, which is why the spectra from the more stable mixture with compound **14** was used for structure analysis. However, the ^{1}H and ^{13}C NMR spectra from the pure compound were taken as 1D traces for better comparison.*

COSY spectrum:

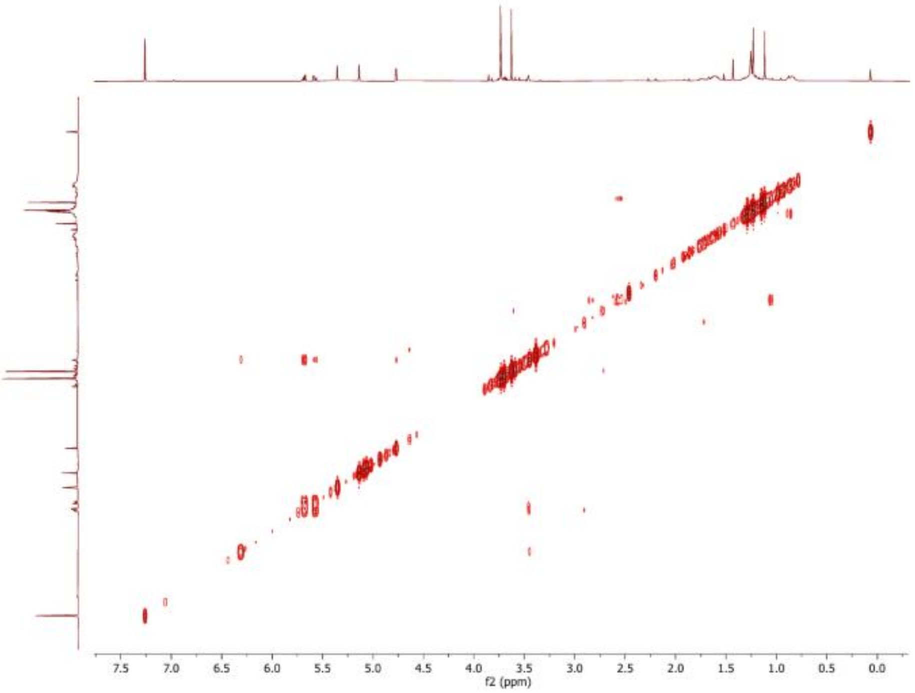

HSQC spectrum:

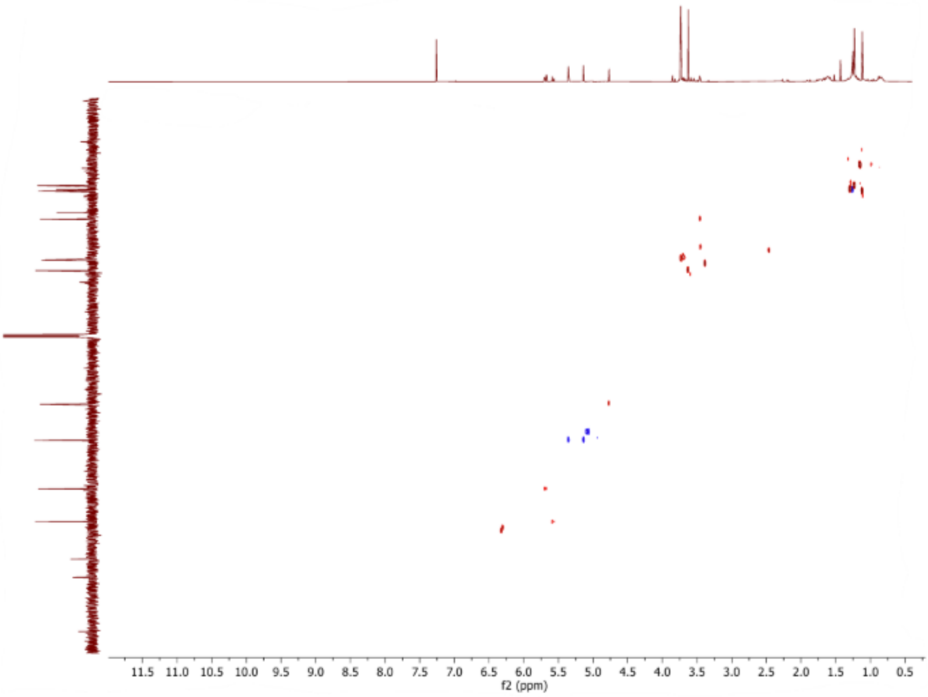

HMBC spectrum:

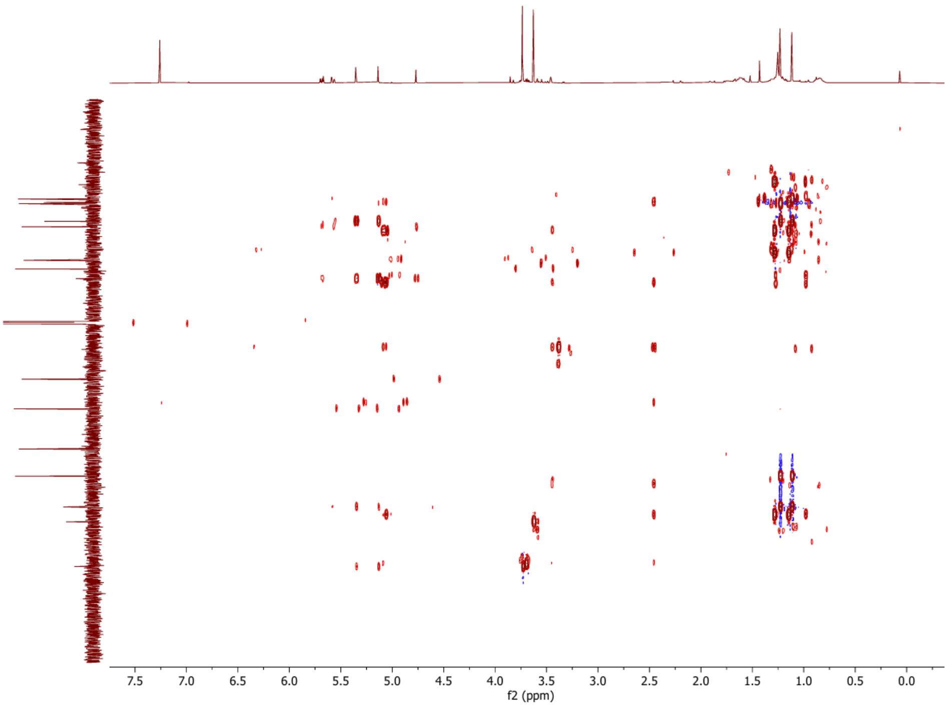

11. References

[1] P. Bracht, F. Bohle, S. Grimme, *Phys. Chem. Chem. Phys.* **2020**, *22*, 7169–7192.

[2] M. J. Frisch *et al.*, *Gaussian 16, Revision C.02*, Gaussian, Wallingford, CT, **2016**.

[3] J. D. Chai, M. Head-Gordon, *Phys. Chem. Chem. Phys.* **2008**, *10*, 6615–6620.

[4] F. Weigend, R. Ahlrichs, *Phys. Chem. Chem. Phys.* **2005**, *7*, 3297–3305.

[5] V. Barone, M. Cossi, *Phys. Chem. A* **1998**, *102*, 1995–2001.

[6] G. Luchini, J. V. Alegre Requena, I. Funez-Ardois, R. S. Paton, *F1000Res.* **2020**, *9*, 291.

[7] Schrodinger, LLC, *The PyMOL Molecular Graphics System*, Version 2.4.1., **2015**.

[8] S. Poplata, T. Bach, *J. Am. Chem. Soc.* **2018**, *140*, 3228.

[9] B. M. Trost, Y. Hu, D. B. Horne, *J. Am. Chem. Soc.* **2007**, *129*, 11781–11790.

[10] J. Meijer, K. Ruitenberg, H. Westmijze, P. Vermeer, *Synthesis* **1981**, 551–554.

[11] *APEX4 Suite of Crystallographic Software, Version 2021-10.0,* Bruker AXS Inc., Madison, Wisconsin, USA, **2021**.

[12] Bruker, *SAINT*, *V8.41*, Bruker AXS Inc., Madison, Wisconsin, USA.

[13] L. Krause, R. Herbst-Irmer, G. M. Sheldrick, D. Stalke, *J. Appl. Cryst.* **2015**, *48*, 3–10.

[14] G. M. Sheldrick, *Acta Cryst.* **2015**, *A71*, 3–8.

[15] G. M. Sheldrick, *Acta Cryst.* **2015**, *C71*, 3–8.

[16] C. B. Huebschle, G. M. Sheldrick, B. Dittrich, *J. Appl. Cryst.* **2011**, *44*, 1281–1284.

[17] Ed. E. Prince, *International Tables for Crystallography Volume C, Mathematical, Physical and Chemical Tables*, International Union of Crystallography, Chester, England, **2006**, 500–502; 219–222; 193–199.

[18] C. R. Groom, I. J. Bruno, M. P. Lightfoot, S. C. Ward, *Acta Cryst.* **2016**, *B72*, 171–179.

[19] D. Kratzert, *FinalCif*, *V153*, https://dkratzert.de/finalcif.html.

12. XYZ Coordinates

65

4f' lowest energy conformer Eopt -1163.179554

C	-0.739686	-0.599149	-1.740520
C	-0.664262	-0.773176	-0.219820
C	-1.098334	-2.244452	0.125825
C	-2.370273	-2.583850	-0.614071
C	-2.857864	-1.900971	-1.639417
C	-2.193546	-0.665931	-2.179589
C	-2.906290	0.594698	-1.687474
C	-2.564012	0.881724	-0.258007
C	-1.610597	0.250170	0.443826
C	-0.020956	-3.260340	-0.290382
C	-1.339216	-2.432202	1.628376
C	-1.487129	0.584155	1.901080
O	-0.431989	0.835662	2.441202
O	-3.202562	1.919333	0.343018
C	-4.530177	2.237955	-0.038721
C	-2.745463	0.566808	2.752168
H	-0.410288	-4.271801	-0.155516
H	0.267933	-3.157779	-1.335598
H	0.870632	-3.151576	0.323982
H	-2.229742	-1.904191	1.968192
H	-1.490821	-3.491477	1.845404
H	-0.479961	-2.086857	2.203870
H	-2.969685	1.585592	3.069158
H	-3.610829	0.158221	2.235769
H	-2.534458	-0.022842	3.645140
H	-5.190231	1.372323	0.057154
H	-4.858407	3.013473	0.649124
H	-4.576504	2.624234	-1.058681
H	-3.986824	0.473369	-1.789992
H	-2.628835	1.458642	-2.301038
H	-2.246287	-0.681363	-3.270078
H	-3.786714	-2.211573	-2.106911
H	-2.893260	-3.468830	-0.261613
H	-0.149979	-1.370052	-2.234943
H	-0.326307	0.364264	-2.037389
O	0.652635	-0.643665	0.279863
C	1.576830	0.259782	-0.303665
C	1.179386	1.728681	-0.177141
C	2.933182	0.011625	0.364585
H	1.684930	0.025152	-1.370971
C	2.213778	2.650656	-0.817372
H	1.067396	1.968214	0.882771
H	0.204945	1.899806	-0.638251
C	3.979753	0.929819	-0.274474
H	2.824595	0.315214	1.413735
C	3.337467	-1.475022	0.380222
C	3.583484	2.399421	-0.195254
H	2.280116	2.397861	-1.883838
C	1.797244	4.110792	-0.700683
H	4.944837	0.786267	0.213943
H	4.116697	0.653420	-1.325490
H	2.542526	-1.991437	0.921162
C	4.631519	-1.704042	1.158739

C	3.428334	-2.104905	-1.007526
H	3.553748	2.711066	0.855751
H	4.338604	3.019134	-0.686912
H	2.527686	4.769548	-1.175782
H	0.827471	4.284071	-1.172528
H	1.714762	4.402852	0.349921
H	5.502294	-1.323607	0.619728
H	4.599026	-1.212788	2.134257
H	4.792228	-2.771338	1.325091
H	2.484370	-2.028165	-1.548841
H	4.207900	-1.634917	-1.612143
H	3.672724	-3.166245	-0.924531
